# Expanding the Palette of Phenanthridinium Cations

**DOI:** 10.1002/chem.201304241

**Published:** 2014-02-19

**Authors:** Andrew G Cairns, Hans Martin Senn, Michael P Murphy, Richard C Hartley

**Affiliations:** [a] WestCHEM School of Chemistry, University of GlasgowGlasgow, G12 8QQ (UK) E-mail: hans.senn@glasgow.ac.ukrichard.hartley@glasgow.ac.uk; [b] MRC Mitochondrial Biology Unit, Wellcome Trust/MRC BuildingCambridge, CB2 0XY (UK)

**Keywords:** density functional calculations, nucleophilic substitution, phenanthridinium, solvation, synthetic methods

## Abstract

5,6-Disubstituted phenanthridinium cations have a range of redox, fluorescence and biological properties. Some properties rely on phenanthridiniums intercalating into DNA, but the use of these cations as exomarkers for the reactive oxygen species (ROS), superoxide, and as inhibitors of acetylcholine esterase (AChE) do not require intercalation. A versatile modular synthesis of 5,6-disubstituted phenanthridiniums that introduces diversity by Suzuki–Miyaura coupling, imine formation and microwave-assisted cyclisation is presented. Computational modelling at the density functional theory (DFT) level reveals that the novel displacement of the aryl halide by an acyclic *N*-alkylimine proceeds by an S_N_Ar mechanism rather than electrocyclisation. It is found that the displacement of halide is concerted and there is no stable Meisenheimer intermediate, provided the calculations consistently use a polarisable solvent model and a diffuse basis set.

## Introduction

**Figure 1:**
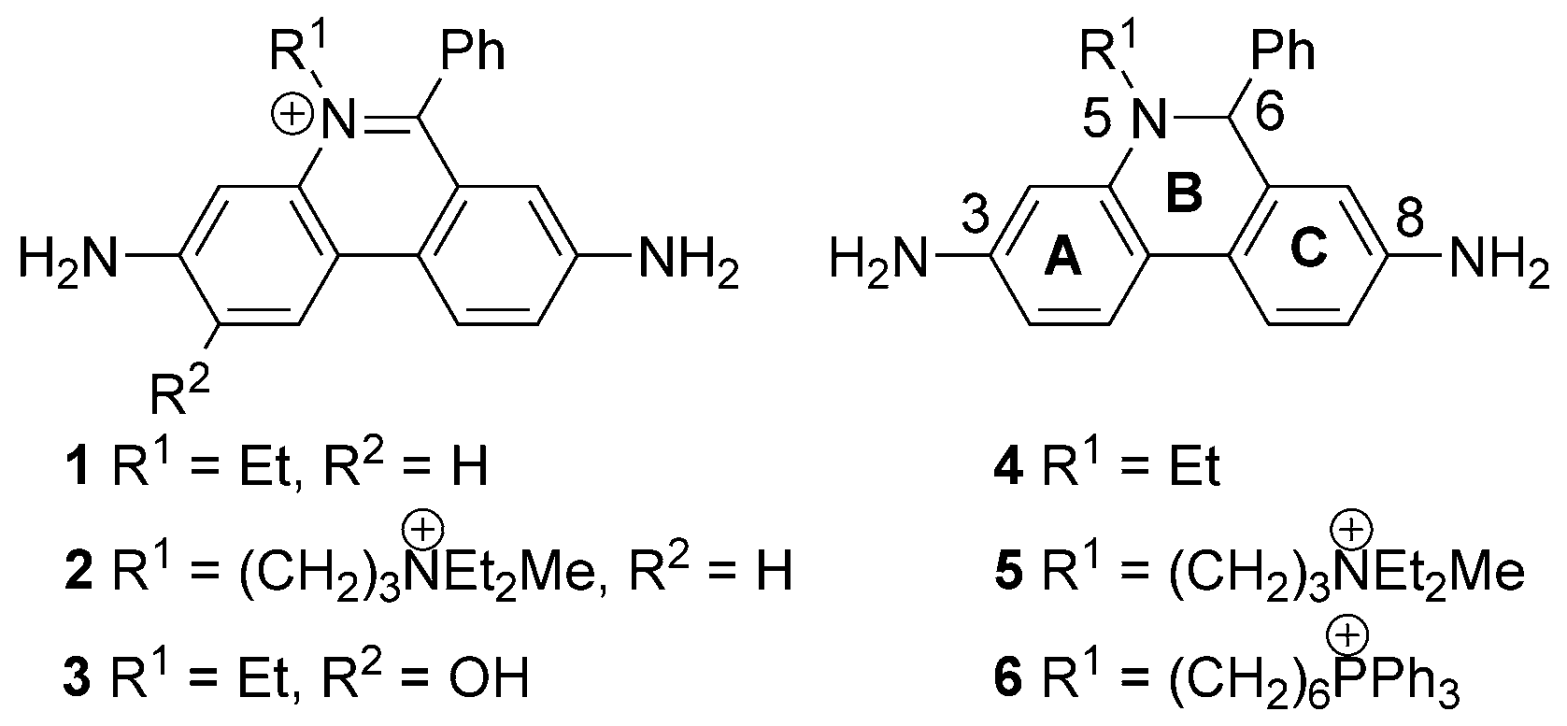
Important phenanthridinium cations 1–3 (counterion unimportant) and phenanthridines 4–6 (substituent numbering and our designation of rings are shown).

5,6-Disubstituted phenanthridinium salts have found wide utility as fluorescent dyes for DNA,[1] as sensors[2] and as potential therapeutics,[3] including for the treatment of trypanosomal infections.[4] The planar phenanthridinium cation can intercalate into duplex nucleic acids,[1, 5, 6] particularly if it has 3- and/or 8-amino groups.[7] The fluorescence of the 3,8-diamino-6-aryl derivatives, such as ethidium bromide (**1**) and propidium iodide (**2**), is hugely enhanced upon intercalation, and both the amino groups and the 6-aryl substituent contribute significantly to this effect (Figure 1).[8] As a result, in spite of its toxicity, **1** is widely used in molecular biology laboratories to detect DNA on agarose gels following electrophoresis,[9] and its displacement from DNA is often used to assess the binding of novel ligands to DNA.[10] Propidium iodide is also a valuable DNA stain employed in flow cytometry and histochemistry.[11] More efficient visualisation of RNA can be achieved by linking a second fluorophore to the 5,6-disubstituted phenanthridinium.[12] Incorporation of phenanthridinium cations into DNA has enabled the study of its redox properties and electron-transfer processes within its double helix.[13]

Phenanthridiniums can inhibit enzymes in ways that are not related to their intercalation into DNA, which would represent an undesirable off-target effect. The most important example is the allosteric inhibition of acetylcholine esterase (AChE) by **2** and other 5,6-disubstituted phenanthridiniums.[14, 15] This allosteric interaction has been exploited to demonstrate the enzyme-induced assembly of AChE inhibitors using in situ Click chemistry,[15, 16] and as a means of detecting AChE.[17]

5,6-Disubstituted dihydrophenanthridines, which are easily prepared by reduction of the corresponding phenanthridiniums, also have useful properties. They can be oxidised in vivo to the corresponding phenanthridiniums by reactive oxygen species (ROS).[2] Such ROS are predominantly produced by reduction of oxygen to superoxide in the mitochondria,[18] and underlie much of the damage in cardiovascular disease, stroke and neurodegeneration. They are also implicated in the process of ageing. The specificity of hydroethidine (**4**) oxidation by superoxide to give the hydroxyethidium cation (**3**) makes this reaction a relatively robust method for assessment of superoxide production in biological systems,[2, 19] and hydropropidine (**5**) provides a useful cell-impermeant analogue.[20] A mitochondria-targeted version, MitoSOX (**6**), allows specific assessment of this ROS in mitochondria.[21] Unfortunately, the detection of the hydroxyethidium (**3**) to infer superoxide concentrations in biological experiments often relies on the enhanced fluorescence when the cation **3** binds to DNA, and this is complicated by the presence of other ethidium derivatives formed by different oxidative processes that produce similar emissions. More accurate quantification requires isolation and HPLC separation of the various phenanthridiniums[2] as so-called exomarkers.[22] Here, the interaction with DNA can be problematic because it adversely affects extraction of the phenanthridiniums from the biological medium and so makes quantification less reliable. Therefore, structures that allow the independent modulation of the redox, DNA intercalating and enzyme interaction properties of this class of compounds would facilitate the rational design of biologically useful probe molecules.

Herein, we present a highly versatile route to 5,6-disubstituted phenanthridiniums so as to allow their properties to be tuned to a desired function, be it redox reactivity or interaction with DNA or enzymes. The key step is a novel cyclisation in which an acyclic *N*-alkylimine displaces an aryl halide intramolecularly. We use DFT calculations to determine the mechanism of this reaction, distinguishing between electrocyclisation and S_N_Ar pathways.

## Results and Discussion

**Scheme 1:**
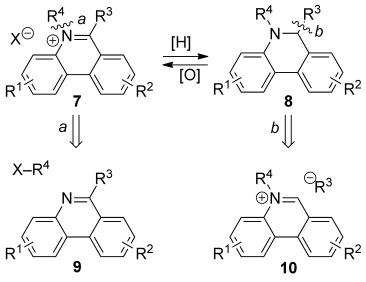
Retrosynthetic analysis of standard approaches to 5,6-disubstituted phenanthridinium salts 7 and phenanthridines 8.

*N-*Alkylphenanthridinium cations (**7**) and *N-*alkyl-dihydrophenanthridines (**8**) bearing an alkyl or aryl substituent at C-6 can be interconverted by simple redox reactions. Two methods have almost invariably been used to construct these structures (retrosynthetic Scheme 1). The first is N-alkylation of the phenanthridine **9**, an often inefficient reaction suffering from the poor solubility of phenanthridines in many organic solvents and requiring powerful alkylating agents, such as alkyl triflates.[8, 13, 17] The 6-substituted phenanthridine precursors needed for N-alkylation have been accessed by a wide range of methods.[23] The second approach involves nucleophilic attack on a phenanthridinium salt (**10**) with a carbanion.[24] The phenanthridinium precursor **10**, which lacks the C-6 substituent, is also generally prepared by N-alkylation. Addition of stabilised carbanions to C-6 can be reversible, allowing interconversion of non-fluorescent and fluorescent compounds **8** and **10**, respectively,[25, 26] a property used by Cronin and co-workers in their design of pH sensors.[26, 27] Nucleophilic additions with Grignard reagents have worked well in the preparation of similar dihydrobenzophenanthridines, which are used to make benzophenanthridinium salts.[28]

There are some promising methods that give direct access to 5,6-disubstituted dihydrophenanthridines and do not involve the processes shown in Scheme 1. In particular, examples of C–H olefination–cyclisation[29] Dötz benzannulation,[30] and a Pictet–Spengler reaction of an *N*-alkylaniline[31] have been reported. A few benzophenanthridiniums have been prepared by cyclisation of *N*-aryl-*N*-alkyl-acetamides using POCl_3_,[32] but the direct synthesis of 5,6-disubstituted phenanthridinium salts by this method is not know.

**Scheme 2:**
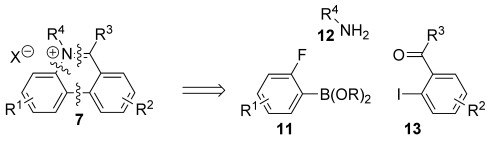
Retrosynthetic analysis of our convergent approach to 5,6-disubstituted phenanthridinium salts 7 beginning from three components 11–13.

We now present a general modular approach to 5,6-disubstituted phenanthridiniums that does not involve N-alkylation. We reasoned that the central ring of the phenanthridinium cation could be assembled in a convergent way from an *ortho*-fluoroarylboronate (**11**), a primary amine (**12**) and an aromatic ketone (**13**) by Suzuki–Miyaura cross-coupling,[33] imine formation and intramolecular nucleophilic aromatic substitution (Scheme 2). Aryl fluorides seemed the most suitable precursors as they react more quickly than other aryl halides in S_N_Ar reactions.[34]

**Scheme 3:**
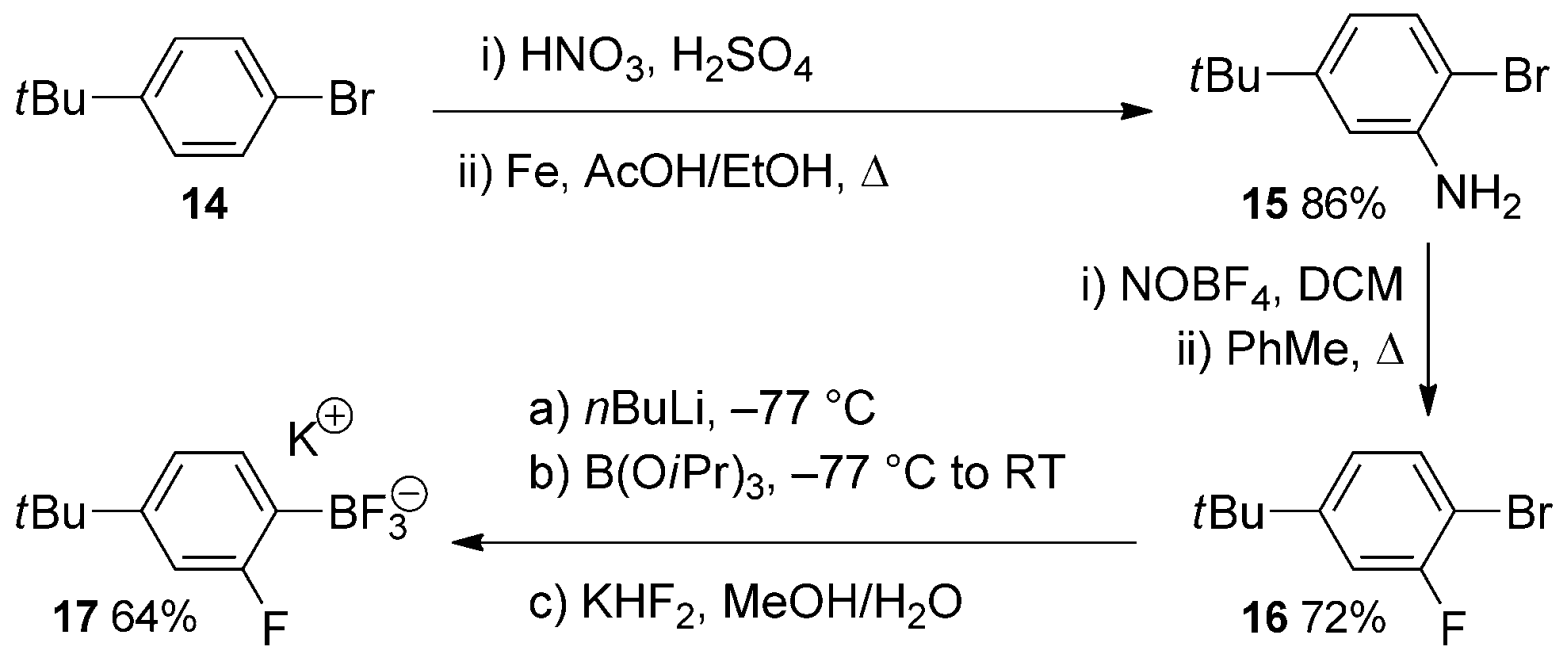
Synthesis of an *ortho*-fluoroborate 17.

Many *ortho*-fluoroarylboron compounds are commercially available and they are also typically straightforward to assemble. For example, aryltrifluoroborate **17** was easily prepared from 4-*tert*-butyl-bromobenzene (**14**; Scheme 3).

**Scheme 4:**
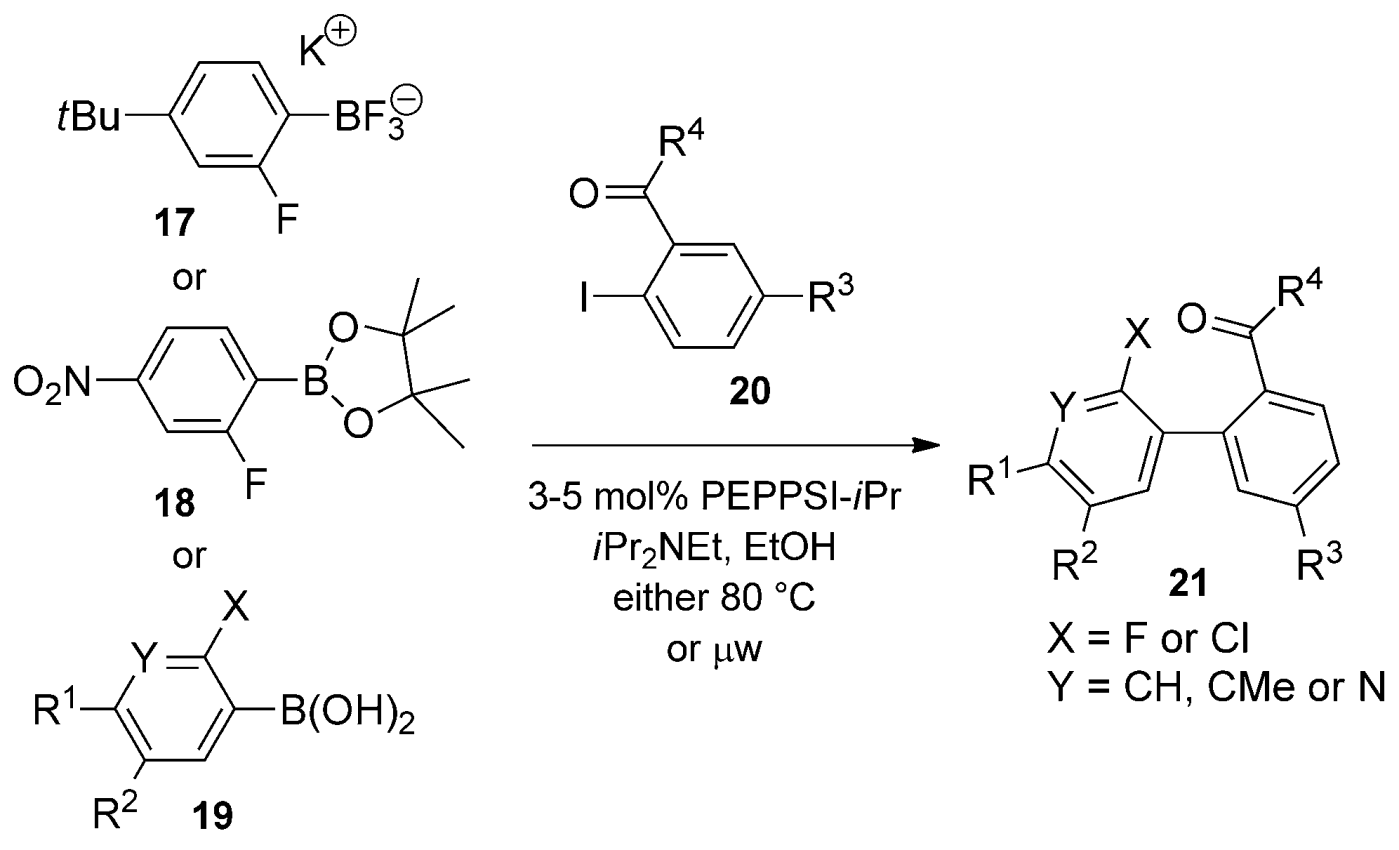
Suzuki cross-coupling to form ketones 21. See Figure 2 for yields and conditions.

**Figure 2:**
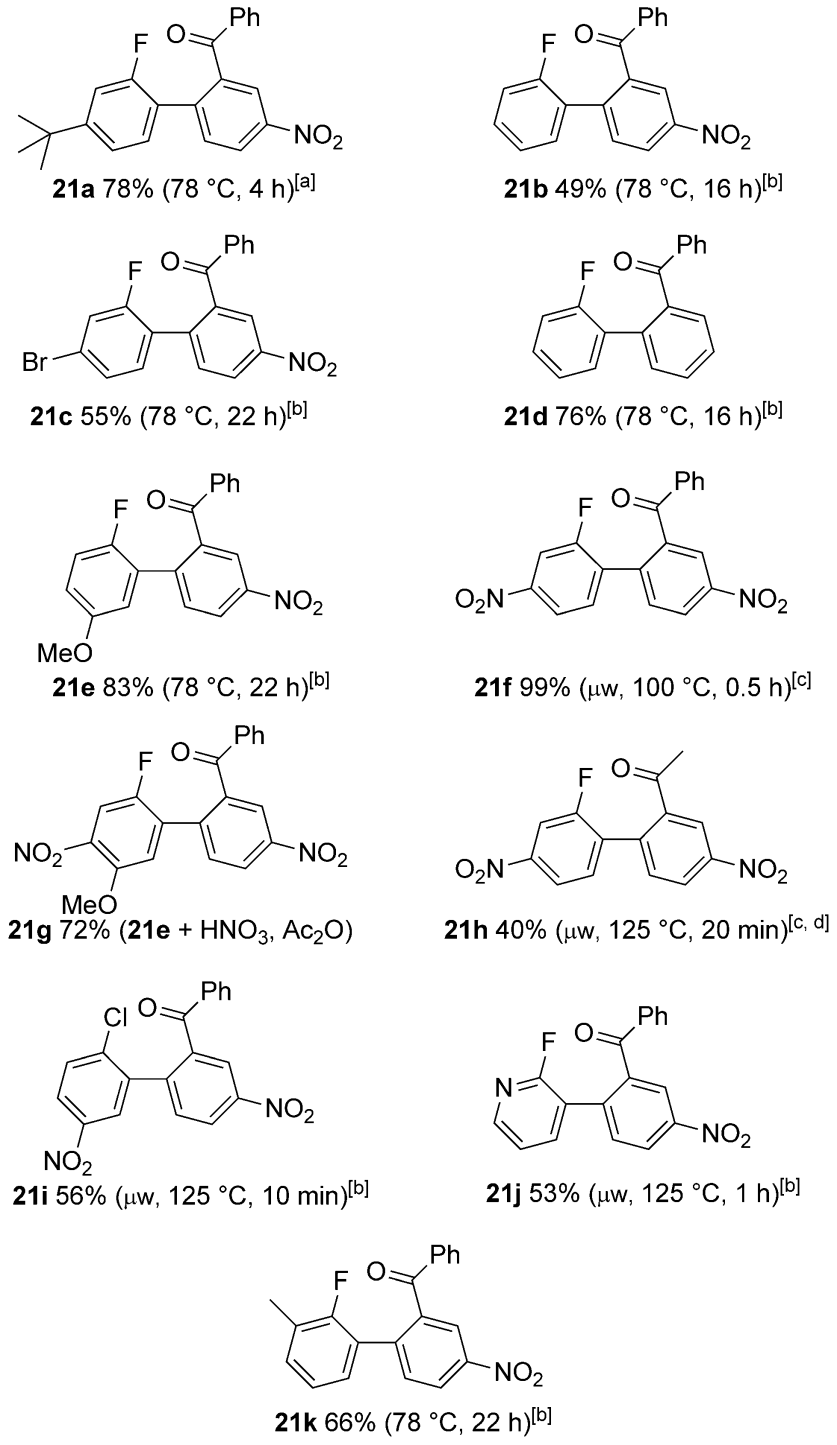
Ketones 21 were prepared from: [a] aryltrifluoroborate 17, [b] the corresponding aryboronic acids 19, or [c] arylboronate ester 18 (yields and conditions in parenthesis). [d] Aryl bromide rather than aryl iodide was used as reactant.

Nitration of aryl bromide **14** and reduction gave aniline derivative **15**. Diazotisation in DCM and solvent exchange gave a toluene suspension of the aryltetrafluoroborate without isolating the potentially explosive intermediate. This underwent the Balz–Schiemann reaction[35] to give aryl fluoride **16** upon heating. Bromine–lithium exchange and borylation was followed by conversion to the aryltrifluoroborate **17**,[36] which is easy to isolate by crystallisation. Arylboron compounds **17**–**19** were coupled with iodoarenes **20** to give biaryl compounds **21** using the air- and moisture-stable palladium N-heterocyclic carbene catalyst PEPPSI-*i*Pr,[37] either under thermal or microwave conditions (Scheme 4 and Figure 2). Microwave conditions were preferred for the more electron-poor arylboron compounds because side-products occurred under thermal conditions. Differences in yields mostly reflected the varying efficiency of the purifications involved. The dinitro compound **21 g** was obtained by nitration of biaryl **21 e**.

**Scheme 5:**
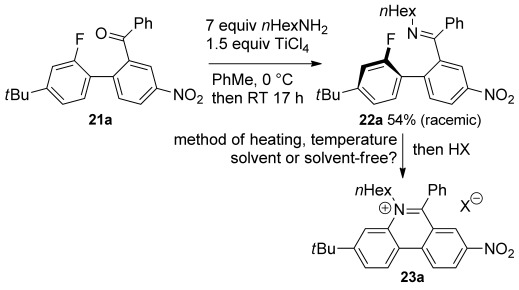
Method development for construction of the B-ring.

Treating 2-arylbenzophenone (**21 a**) with hexylamine and titanium tetrachloride produced imine **22 a**, which was isolated by chromatography in modest yield and was used to investigate the conditions for the proposed cyclisation (Scheme 5). Interestingly, the CH_2_N exhibited diastereotopicity indicating that the biaryl unit is not planar and there is restricted rotation about the C–C bond that links the two rings. Heating to 200 °C in nitrobenzene led to the formation of a 6-phenylphenanthridine, presumably by way of phenanthridinium formation and fluoride-induced elimination. Transferring the imine **22 a** to a short-path distillation (Kugelrohr) apparatus in chloroform, distilling off the chloroform so that the neat sample could then be heated to 100 °C for 1 h gave complete conversion from the imine **22 a**. This resulted in a mixture of compounds in which the A-ring fluoride had been substituted. Treatment with TFA then gave the desired phenanthridinium salt **23 a** in 79 % overall yield from the imine **22 a**. Similar use of acid to prepare phenanthridinium salts from pseudo-bases (the neutral adduct of hydroxide attack on the *N*-alkylimminium group) is well-known.[38] Although this solventless procedure for the preparation of *N*-alkylphenanthridinium salt **23 a** from imine **22 a** was high yielding, it led to charring for other imines that solidified upon removal of the chloroform. A better procedure was to heat a solution of the imine in THF in the microwave at 100 °C for 2 h. The reaction was faster in acetonitrile, but this is more expensive and gave minor impurities so it was only used for less reactive imines.

**Scheme 6:**
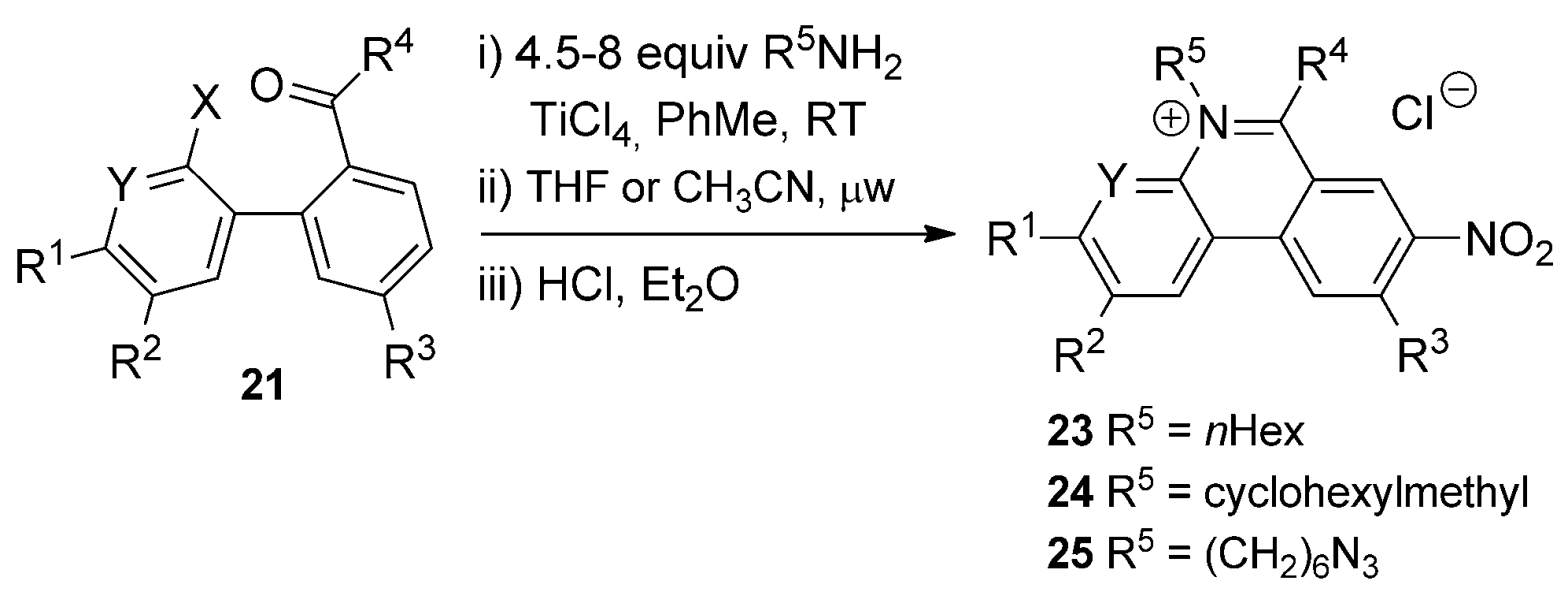
Exemplification of *N*-alkylphenanthridinium synthesis. See Figure 3 for yields and conditions.

**Figure 3:**
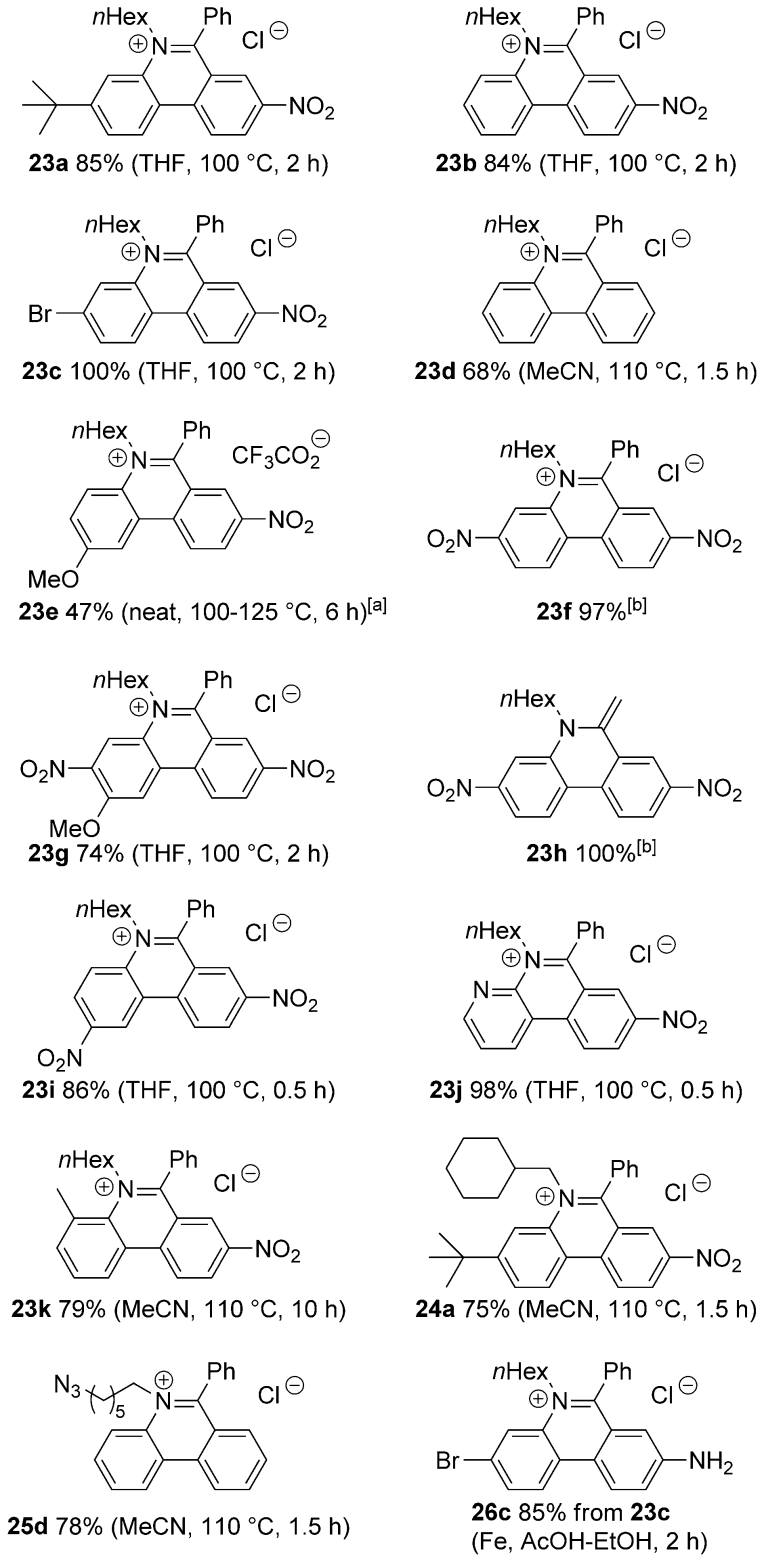
Phenanthridinium salts 23–25 prepared from ketones 21 by imine formation and microwave irradiation according to Scheme 6 (yields and conditions in parenthesis) and phenanthridinium salt 26 c prepared by reduction of nitro compound 23 c. [a] Prepared under thermal conditions [b] Second step unnecessary as spontaneous cyclisation occurred.

We then explored the range of substrates that could be used in an optimised three-step procedure in which the crude imine is heated in the microwave after an aqueous work-up, and the product mixture is treated with ethereal HCl to form the closed phenanthridinium salts **23**–**25**, which in many cases were isolated by simple precipitation (Scheme 6 and Figure 3).

8-Nitrophenanthridiniums **23 a–c** were produced in good yields under these conditions. An electron-withdrawing group is not required for reaction and the simple 5,6-disubstituted phenanthridinium **23 d** could be prepared in good yield using acetonitrile as solvent and a slightly higher temperature. However, placing an electron-donating methoxy group *para* to the fluoride significantly impeded reaction even when the 8-nitro group was present, so that phenanthridinium **23 e** was produced in lower yield under all conditions (the best yield was obtained under the original solventless conditions with extended heating). Introduction of a second electron-withdrawing nitro group gave rise to a dramatic acceleration and the second step was unnecessary for the preparation of 3,8-dinitrophenanthridinium (**23 f**) in very high yield, because the cyclisation occurred spontaneously during removal of the toluene on the rotary evaporator with modest heating. Unsurprisingly, the synthesis of phenanthridinium **23 g**, which has an electron-donating methoxy group *para* to the fluoride, required heating. However, unlike the methoxyphenanthridinium **23 e** without a 3-nitro group, reaction proceeded in good yield under the standard conditions in THF. The acetophenone derivative **21 h** also cyclised spontaneously and was isolated in quantitative yield as the enamine **23 h** following an alkaline wash. The 2,8-dinitrophenanthridinium **23 i** and 4-aza-8-nitrophenanthridinium **23 j** were also produced in high yield. Both formed more easily than phenanthridiniums **23 a–c** bearing only one nitro group, even though conversion of ketone **21 i** to phenanthridinium **23 i** involved displacement of a chloride rather than a fluoride.

A 4-methyl group greatly impedes reaction, presumably because of steric interactions, so that extensive microwave heating in acetonitrile was required to prepare 2-methyl-8-nitrophenanthridinium **23 k**. Steric interactions were further investigated with the use of a more sterically hindered amine, cyclohexylmethylamine. The phenanthridinium **24 a** was isolated in good yield, but unlike phenanthridinium **23 a** required the higher temperature conditions in acetonitrile.

An azido group provides a way of introducing diversity through Click chemistry[39–41] and Staudinger ligation;[39] as such it is a useful tag in materials chemistry[40] and because it combines with alkynes under bioorthogonal conditions has found wide application in chemical biology.[39, 41] Indeed, a library of 5,6-disubstituted phenanthridiniums bearing azido tags was used for the in situ generation of AChE inhibitors.[16] Therefore, we demonstrated that the azido tag was straightforwardly introduced by our method. Thus, combining 6-azidohex-1-ylamine with ketone **21 d** gave phenanthridinium **25 d** in good overall yield.

The bromo group in phenanthridinium **23 c** also gives a potential site for the introduction of diversity through cross-coupling reactions, and so was chosen to exemplify access to the important 8-amino compounds. Reduction by iron in AcOH/EtOH at 40 °C gave phenanthridinium **26 c** in good yield.

### Computational mechanistic study

**Scheme 7:**
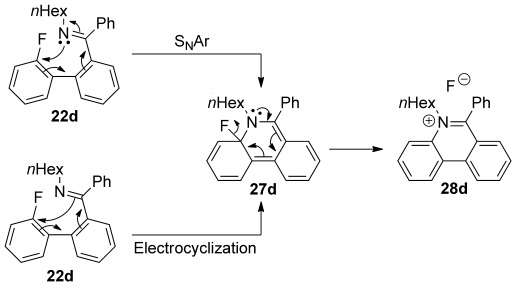
Two putative mechanisms for the formation of phenanthridinium salt 28 d from *N*-alkylimine 22 d differing in the mode of cyclisation to give Meisenheimer complex 27 d.

The nucleophilic substitution of aryl halides by pyridine derivatives is well known,[42, 43] particularly for the preparation of Zincke salts[42] (2,4-dinitrophenyl derivatives that have a variety of uses). However, similar reactions by cyclic *N*-alkylimines are extremely rare[44] and such reactions by acyclic *N*-alkylimines almost unprecedented (we found one example[45]). The reaction is mechanistically interesting because cyclisation of, for example, imine **22 d** to give the corresponding Meisenheimer complex **27 d** could occur either by electrocyclisation involving the C=N π bond,[46] or by the traditional S_N_Ar mechanism through nucleophilic attack on the aromatic ring by the nitrogen lone pair (Scheme 7). The putative Meisenheimer complex **27 d** would then fragment to give the phenanthridinium fluoride **28 d**, which would be a precursor to the chloride salt **23 d**. Since the line of attack in the first step is different for the two mechanisms, we reasoned that they could be distinguished by calculating the structure of the transition state.

**Scheme 8:**
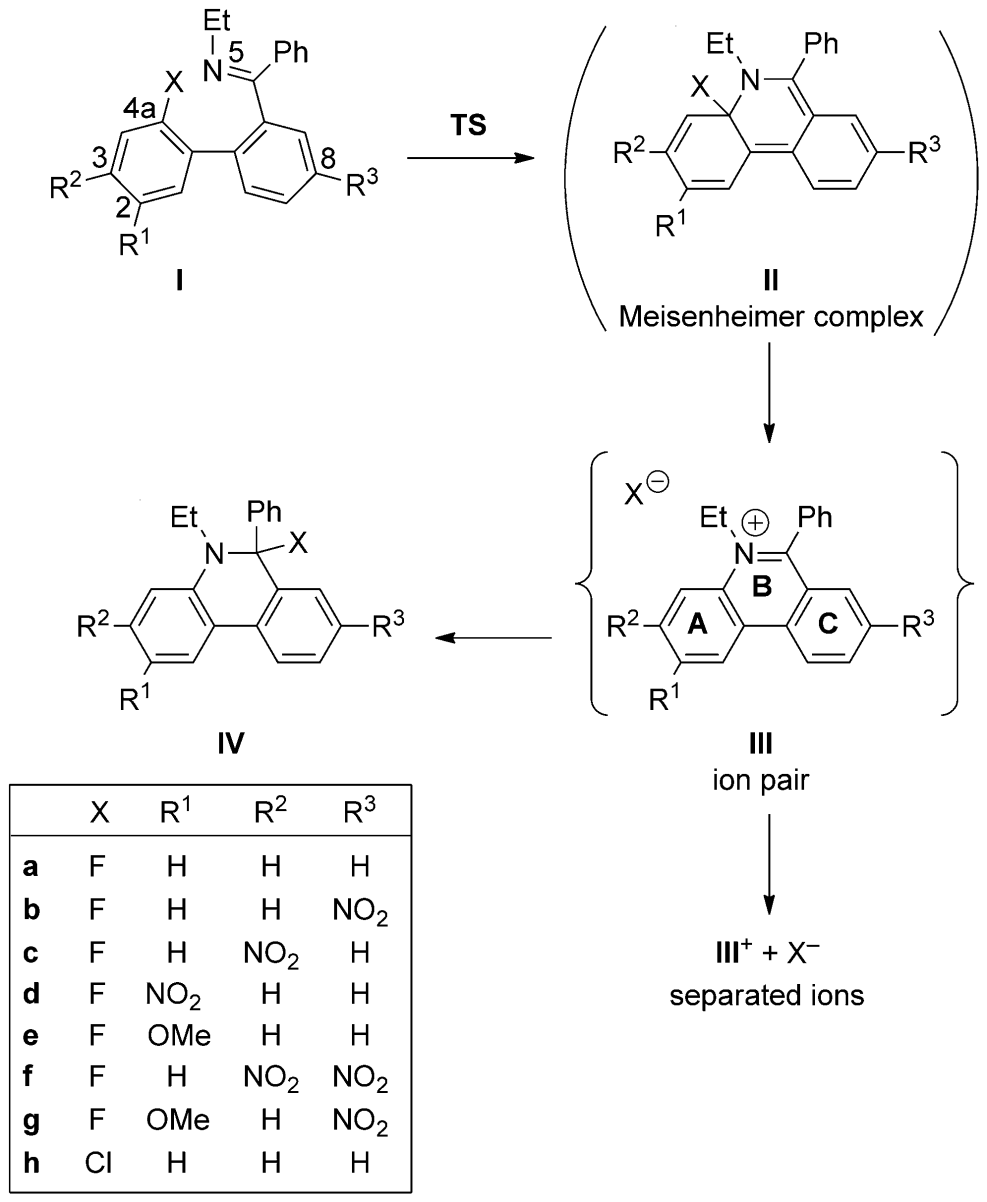
Cyclisation reaction with transition state and intermediates as identified computationally. The Meisenheimer complex II is not a stable minimum in MeCN solution.

We, therefore, embarked on a computational study at the DFT level (M06-2X/def2-TZVP+) to elucidate the mechanism of the cyclisation reaction, including structures and energies of possible intermediates and transition states. Staying close to the experimentally studied compounds, we considered the imines **Ia–h** as reactants (Scheme 8). The series covers a range of substitution patterns, which will allow us to analyse the influence of substituent effects on the reaction. All computational results refer to reaction conditions of *T*=383 K and *P*=320 kPa, corresponding to the conditions used in the cyclisation of **22 d** to **23 d**. Unless noted otherwise, all calculations used a polarisable continuum solvent model of acetonitrile, which was calibrated for the reaction conditions. For comparison, we also considered the reaction of **Ia** in vacuum.

**Figure 4:**
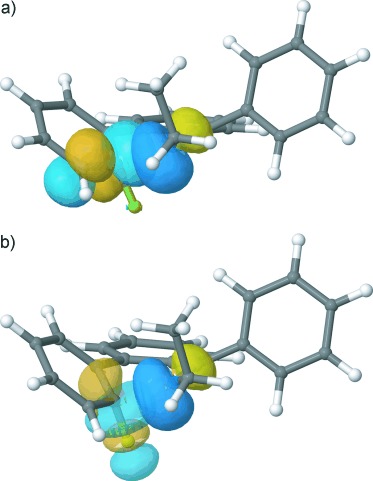
Optimised structure of the transition state TSa with NBOs involved in important donor–acceptor interactions. a) Donation of imine lone pair n_N_ into aryl π*(C4a=C4); b) Donation of n_N_ into the C–F antibonding orbital, σ*(C4a–F).

The cyclisation transition state, **TS**, could readily be located for all cases. Its structure is characteristic of an S_N_Ar transition state (Figure 4). As substituent and environment effects on the structure of **TS** are minor, we are using **TSa** as the type specimen in the following discussion. The sp^2^ lone pair of the attacking imine nitrogen (N5) points straight at the halogen-bearing aryl carbon (C4a), at an angle of 125° to the aryl plane. The coordination geometry at C4a is clearly pyramidal, with the C–F bond out of the aryl plane by 36°. The C–F bond is lengthened from 1.35 Å in the reactant **Ia** to 1.40 Å in **TSa**. The C=C bonds adjacent to C4a are also lengthened slightly (from 1.39 to 1.42 Å), while the imine C=N bond remains practically unchanged (1.27 to 1.28 Å).

The characterisation of the cyclisation reaction as a nucleophilic attack, rather than an electrocyclic process, was further corroborated through a natural bond orbital (NBO) analysis.[47] In essence, NBOs are constructed such as to correspond optimally to a Lewis picture of localised (anti-)bonding orbitals and lone pairs (in contrast to the often highly delocalised canonical molecular orbitals).

Figure 4 shows the imine lone pair together with the two principal empty orbitals it is interacting with. The lone pair donates substantial electron density (0.4 e) into the aryl antibonding orbital π*(C4a=C4) and the C–F antibonding orbital σ*(C4a–F). Both interactions are energetically highly favourable, Δ*E*_deloc_=−519 and −126 kJ mol^−1^, respectively; the former exceeds the next smaller donor–acceptor interaction more than threefold. In contrast, the imine C=N π and π* orbitals have no significant interaction with any orbital on the halogen-bearing A-ring. The incipient C–N bond therefore arises principally from the interaction of the imine lone pair with the aryl π* adjacent to the carbon, which is the signature of a classic S_N_Ar attack.

The free-energy barrier for the formation of the C–N bond is 103 kJ mol^−1^ in the parent **TSa** (Table 1). The barriers show the substituent effects expected for a nucleophilic mechanism, which is promoted by stabilising the developing partial negative charge on the attacked moiety: electron-withdrawing substituents lower the barrier (**TSb**, **c**, **d**, **f**), while donors increase it (**TSe**). The stabilising effect of the nitro substituent is strongest when it is *para* to the halogen (**TSd**, R^1^=NO_2_) because of direct conjugation to C4a. However, it is important to note the still significant stabilisation by the strong inductive effect of a *meta* nitro group (**TSc**, R^2^=NO_2_).[48] In contrast, a nitro group on the C-ring provides less stabilisation even when it is formally in conjugation with the same carbon atom (**TSb**, R^3^=NO_2_), because the A- and C-rings are not co-planar (the biphenyl torsion is −54° in the reactant **Ia** and reduces to −37° in **TSa**). For the cases considered, substituent effects on the barrier height are additive: the barriers for the disubstituted **TSf** and **TSg** are within 2 kJ mol^−1^ of the values predicted from the increments derived from the monosubstituted cases.

**Table 1:** Gibbs free energies^[a]^ relative to I along the reaction sequence shown in Scheme 8. Also given are the lengths of the forming (N5–C4a) and breaking (C4a–X) bonds in the transition state

	Δ*G* [kJ mol^−1^]			*d*^≠^ [Å]		
	**TS**	**III**	**III**^+^+X^−^	**IV**	N5–C4a	C4a–X
**a**	103	−65	−81	−79	1.85	1.40
**b**	98	−57	−74	−84	1.88	1.39
**c**	93	−60	−72	−93	1.87	1.39
**d**	72	−52	−71	−89	1.96	1.37
**e**	115	−70	−90	−78	1.84	1.41
**f**	90	−51	−59	−88	1.88	1.39
**g**	112	−61	−77	−76	1.87	1.40
**h**	120	−126	−133	^[b]^	1.88	1.87
**a_vac_**^[c]^	112	80^[d]^	440	−80	1.81	1.39

[a] Calculated at M06-2X/def2-TZVP+ level in polarisable continuum MeCN (*ε*_r_=22.5) for *T*=383 K, *P*=320 kPa. [b] The 6-chlorophenanthridine **IVh** is not a stable minimum, but dissociates into the ion pair **IIIh** during structure optimisation. [c] Calculated in vacuum. [d] Energy of **IIa_vac_**. In vacuum, the ion pair **IIIa_vac_** is not stable, but collapses to **IIa_vac_**.

The reactivity towards nucleophilic attack is significantly reduced in the chloro compound (**TSh**) compared to the fluoro congener (**TSa**), again as expected for an S_N_Ar reaction. The more electronegative the leaving group, the more positive is the partial charge on the attacked carbon and the lower in energy is the acceptor σ*_CX_ orbital, which translates into higher reactivity. In **Ia** and **Ih**, the Hirshfeld charges on the halogen are −0.094 e (F) and −0.075 e (Cl), while the charges on the carbon are 0.080 and 0.031 e, respectively. Similarly, Δ*E*_deloc_(n_N_→σ*_CCl_)=−60 kJ mol^−1^ in **TSh**, compared to −126 kJ mol^−1^ in **TSa**, indicating the reduced stabilisation of the transition state by the less electronegative halogen.

The effect of solvation on the barrier is remarkably small: **TSa** is only 9 kJ mol^−1^ lower than **TSa_vac_**. The vacuum transition state is slightly later, judging from the shorter N–C distance (1.81 vs. 1.85 Å); however, there is hardly any difference in the degree of activation of the C–F bond (1.39 vs. 1.40 Å). This is another clear indication that the transition state corresponds to the formation of the C–N bond, while the C–X bond, although weakened, is essentially maintained. The transition state can therefore be described as Meisenheimer-like in terms of its geometric and electronic structure.

Although we have not collected any experimental kinetic data in this study, we may still use the yields and reaction conditions as indicators for the required activation energies. Comparing the unsubstituted **23 d** (corresponding to the cyclisation product of **Ia**) with **23 b** (**Ib**), **23 e** (**Ig**), and **23 f** (**If**; Figure 3), we find that the yields and required conditions agree with the trends in the computed free-energy barriers.

Energy-minimising the S_N_Ar transition states towards products, we expected to obtain the Meisenheimer complexes **II**. However, we found that **IIa–h** are not stable minima in MeCN solution. Rather, the C–X bond is spontaneously cleaved to form the ion pairs **III**, in which the halide anion is located at a distance of approximately 3 Å directly “beneath” the nitrogen N5 of the planar phenanthridinium cation. We are therefore dealing with a concerted, one-step S_N_Ar reaction, in which the formation of the Meisenheimer σ-complex constitutes the rate-limiting barrier, while its decomposition is barrier-free.

Energetically, these reactions are exergonic by 50–70 kJ mol^−1^ (Table 1). The driving force is mainly enthalpic, due to the aromatisation of the B-ring and the strong solvation of the anion. For X=Cl (**IIIh**), the reaction is even more exergonic, despite the weaker solvation of Cl^−^ compared to F^−^, because of the weaker C–Cl bond.[49] For all cases in solution, the complete separation of the ion pair into free ions **III^+^**+X^−^ is further exergonic, which is driven entropically; enthalpically, the ion pair is favoured over separated ions (see the Supporting Information). The relative stability of the phenanthridinium cations is governed by substituent effects, which act opposite to the situation in electron-rich **TS**: acceptor substituents destabilise the cation (**IIIb**, **c**, **d**, **f**), whereas donor substituents stabilise it (**IIIe**).

Instead of remaining as separated ions, the halide may reattach to the phenanthridinium to form the neutral 6-halophenanthridines **IV**. The thermodynamic viability of this step depends on the substituents; for X=F, the reaction free energies relative to separated ions are between −23 kJ mol^−1^ (**IVf**) and +12 kJ mol^−1^ (**IVe**). On enthalpic grounds, **IV** is always more stable than the ion pair or separated ions (see the Supporting Information). The relative stability of the 6-fluorophenanthridines **IV** follows the same pattern as in the **TS**, opposite to the substituent effects in the cations. In contrast to the fluoro compounds **IVa**–**g**, **IVh** with X=Cl is not a stable energy minimum, but spontaneously dissociates into the ion pair **IIIh**. This is in pleasing agreement with experiment, in which the original fluoride is exchanged for chloride during the work-up (step iii in Scheme 6) and phenanthridinium chlorides are obtained exclusively.

**Figure 5:**
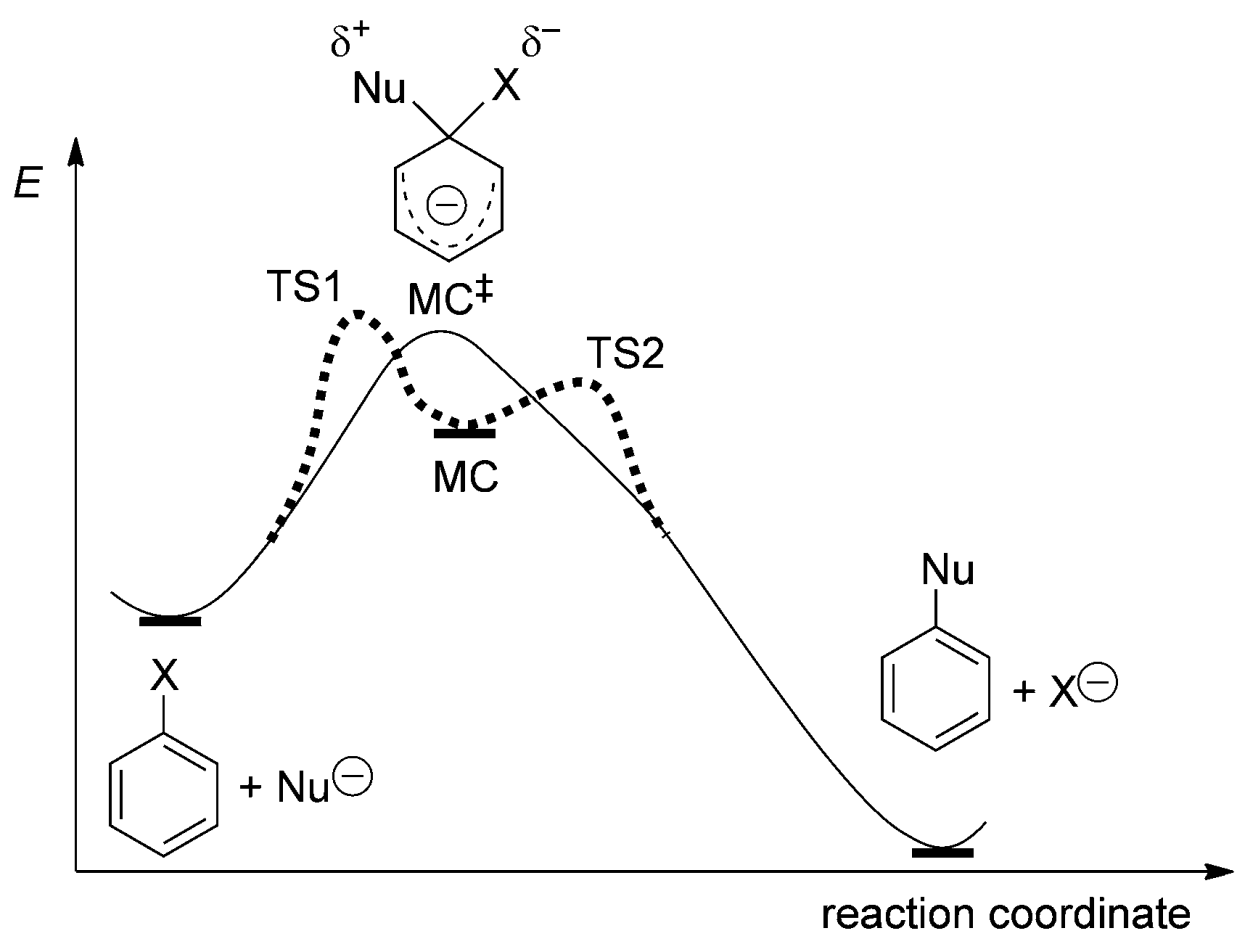
The Meisenheimer complex (MC) can be either a transition state (solid black line) or an intermediate (dashed grey line) along the S_N_Ar reaction coordinate.

The question of whether the Meisenheimer σ-complex is a stable minimum or a transition state along the reaction coordinate of the S_N_Ar reaction, that is, whether the reaction is stepwise or concerted, has been addressed in a number of previous computational studies (Figure 5).[50–56] The answer depends on the nature of the nucleophile (neutral/anionic, nucleophilicity), the leaving group (bond strength, stability of free leaving group), the arene core (substitution pattern), and the reaction medium (gas phase, solvent polarity). For reactions of fluoroarenes (mostly fluorobenzenes) with various nucleophiles, the σ-complex was in most cases found to be an intermediate,[50–53] only two studies reported it to be a transition state: in the reaction of *para*-NO_2_C_6_H_4_F with NH_3_ or NH_2_Me[50] and in the reaction of C_6_H_5_F with Me_2_NSiMe_3_ or Me_2_PSiMe_3_.[54] Both cases are special in that the departing fluoride can bind to the nucleophile (hydrogen bonding in the former, formation of a fluorosilane in the latter) and is therefore greatly stabilised, despite both reactions being in vacuum. Two recent studies have highlighted that the potential-energy surface in the vicinity of the Meisenheimer complex can be very flat, so that even small changes in the computational method can determine its nature. For instance, in the reaction of pentafluoropyridine with NH_3_, the Meisenheimer complex is only prevented from decomposing spontaneously by a vanishingly low barrier (0.1 kJ mol^−1^) at the B3LYP/6-31+G(d,p)/PCM(water) level,[51] but the barrier increases to a significant 19 kJ mol^−1^ when the M06-2X functional is used. For the substitution of a phenylamidate by an amine in polarisable continuum MeOH, the existence of a stable Meisenheimer intermediate was similarly found to depend on the choice of exchange-correlation functional.[55]

Together with the present results, these observations point to the key factor that determines the nature of the Meisenheimer complex in S_N_Ar reactions: it is the balance between the bond strength of the leaving group X and the stabilisation of free X^−^. For X=F in particular, the strong aryl–F bond, which in the Meisenheimer complex is already close to a weaker C(sp^3^)–F bond, must be compensated for by a stabilisation of the incipient F^−^ that acts early on along the reaction coordinate. Unless stabilisation is provided as soon as the C–F bond begins to break and significant negative charge to build up on the fluoride, the Meisenheimer complex will become a minimum, with a barrier to decomposition.

In general, solvation is the single most important factor stabilising the departing (anionic) leaving group. Accordingly, in the absence of solvent, we find that the Meisenheimer complex, **IIa_vac_**, is stable (Table 1), as separating charges is highly disfavoured in vacuum, which agrees with previous studies.[50, 52] While an explicit representation of the solvent combined with extensive configurational sampling (e.g., a Monte Carlo QM/MM approach[53]) may be required to model reliably the reaction in polar-protic solvents, we believe that a much simpler polarisable continuum model is adequate for dipolar-aprotic solvents, like MeCN. However, it is important that the model accurately reproduces the dominating solvation of the free anion. We have therefore taken great care to calibrate our solvation model to the best available experimental solvation data for F^−^ and Cl^−^. Moreover, it is crucial that structures are fully optimised in solution as the potential-energy surfaces differ qualitatively between vacuum and solution. Simply correcting the energies of vacuum structures for solvation effects will necessarily miss the possibility of spontaneous decomposition of the σ-complex.

The second requirement to model the reaction reliably is an adequate description of the electronic structure. Crucially, the localised negative charge of the free anion must be described sufficiently well otherwise its stability will be artificially underestimated. This is not possible without a reasonably large basis set that includes diffuse functions. We initially ran exploratory calculations with the def2-SVP basis set, which predicted stable Meisenheimer intermediates for all cases with X=F; it was only for X=Cl that we obtained the ion pair (see the Supporting Information). The def2-TZVP+ basis set, which includes diffuse functions on all atoms, provided a qualitatively different picture in which the σ-complexes are not stable. Again, structures need to be optimised at the higher level; single-point corrections will fail to capture the effect. Where both basis sets yield qualitatively the same structure (e.g., for the transition states **TS**), energies differ insignificantly (<5 kJ mol^−1^).

## Conclusion

In summary, we present a versatile, efficient and convergent route to 5,6-disubstituted phenanthridiniums using three easily accessible components: arylborons, *ortho*-iodoaryl ketones and primary amines. The key step in the synthesis is the novel microwave-assisted intramolecular displacement of an aryl halide by an acyclic imine. We demonstrate through DFT calculations that the reaction proceeds by an S_N_Ar rather than an electrocyclic mechanism, and that it is concerted and does not involve a stable Meisenheimer complex. Of significant importance to the computational work is the proper modelling of the solvated free anion. The concerted mechanism for the S_N_Ar that comprises our key step is very unusual: the vast majority of examples of this type of reaction appear to involve Meisenheimer complexes as stable intermediates. The use of our synthetic method provides access to a wide range of structural analogues and should enable the development of phenanthridinium probes to respond to redox, DNA intercalation and enzyme reactivity independently.

## Experimental Section

### General procedure for imine formation-cyclisation using conversion of ketone 21 j into 23 j as an example

A solution of titanium tetrachloride in dry toluene (1 m, 2.40 mL, 2.40 mmol, 1.49 equiv) was added over 5 min to a stirred solution of benzophenone **21 j** (520 mg, 1.61 mmol, 1.00 equiv) and dry hexylamine (1.50 mL, 11.4 mmol, 7.05 equiv) in dry toluene (5.0 mL) at 0 °C. The reaction was allowed to warm to RT and stirring was continued for 20 h. The mixture was poured into H_2_O, then filtered through Celite eluting with DCM. HCl_(aq.)_ (1 m) and more DCM were added to the filtrate, the mixture was shaken and the layers were separated. The aqueous layer was re-extracted (DCM×2). The combined organics were washed with 1 m HCl_(aq.)_ then NaHCO_3(aq.)_ and dried (MgSO_4_) then filtered and the solvent was removed under reduced pressure. From the crude yield of 631 mg of brown solid a portion (100 mg, 0.25 mmol, 1.00 equiv) was transferred into a microwave tube (10 mL) containing dry THF (5.0 mL). The tube was filled with argon and sealed, and then heated in a microwave to 100 °C for 30 min. It was then cooled and transferred to a round-bottomed flask in dry CHCl_3_. Then the solvent was removed under reduced pressure. The residue was dissolved in dry Et_2_O and then precipitated with 2 m ethereal HCl (1.00 mL, 2.00 mmol, 8.13 equiv). The solvent was removed under reduced pressure and then the solid triturated with pet. ether and dried under reduced pressure to give 4-aza-5-(hex-1′-yl)-6-phenyl-8-nitrophenanthridinium chloride **23 j** as a white amorphous solid (106 mg, 98 %).

Variations to reaction times, solvent and temperature are shown in Figure 3. The characterisation data and procedures for all compounds are provided in the Supporting Information. Assignment of the ^1^H NMR spectra of phenanthridiniums **23**–**25** was achieved with the assistance of COSY, HMQC and NOESY and agree well with those described by Luedtke et al.[5]

### Computational methods

All calculations were done with the Gaussian 09 program.[57] We chose the M06-2X[58] exchange-correlation functional for its good performance in organic thermochemistry and reaction barriers and its ability to describe accurately dispersive interactions.[59] Production calculations used the def2-TZVP+ basis set, which we derived from def2-TZVP[60] by adding one diffuse function per valence angular momentum. Gibbs energies were calculated using the standard rigid rotor/harmonic oscillator approximation for *T*=383 K, *P*=320 kPa. The SMD solvation model was used as implemented in Gaussian, which combines the IEF-PCM polarisable continuum model for electrostatic solvation with the SMD non-electrostatic terms. We specified non-default values for the following solvation parameters: dielectric constant of MeCN, *ε*_r_(383 K)=22.5; *R*_solv_(F)=1.63 Å, *R*_solv_(Cl)=2.48 Å. Structures were fully optimised in solvent. Minima and transition states were verified through the correct number of imaginary frequencies. The IRC was followed for ten steps to either side of transition states, followed by optimisation to the adjacent minimum. NBO analyses were done using the NBO 6.0 program.[61] Additional details, in particular on the calibration of the solvation model, can be found in the Supporting Information.

## Supporting Information

As a service to our authors and readers, this journal provides supporting information supplied by the authors. Such materials are peer reviewed and may be re-organized for online delivery, but are not copy-edited or typeset. Technical support issues arising from supporting information (other than missing files) should be addressed to the authors.

miscellaneous_information
