# Supplementary material for: Expanding the Palette of Phenanthridinium Cations
Source: Chemistry. 2014 Feb 19;20(13):3742–51. doi: 10.1002/chem.201304241 (PMC4164275; doi:10.1002/chem.201304241)
Supplement: Supplementary file 1 — miscellaneous_information [file chem0020-3742-sd1.pdf]

CHEMISTRY
A European Journal

Supporting Information

© Copyright Wiley-VCH Verlag GmbH & Co. KGaA, 69451 Weinheim, 2014

Expanding the Palette of Phenanthridinium Cations

Andrew G. Cairns,[a] Hans Martin Senn,*[a] Michael P. Murphy,[b] and Richard C. Hartley*[a]

chem_201304241_sm_miscellaneous_information.pdf

Expanding the palette of phenanthridinium cations

Andrew G. Cairns, Hans Martin Senn, Michael P. Murphy,
and Richard C. Hartley

Supplementary Information 1

Supplementary Synthetic Details

General Experimental:

^{1}H NMR and ^{13}C NMR spectra were obtained using Bucker-ACS 60 spectrometers operating at 500 and 400 MHz respectively. All coupling constants are recorded in Hz. DEPT and DEPTQ was were used to assign C, CH, CH_2 and CH_3. 2D techniques including COSY, HSQC, NOESY and, the ^{19}F equivalent of HSQCs and ^{19}F decoupled protons ^{1}H NMR spectra were used to aid assignment of the ^{1}H NMR spectra. EI and CI mass spectra were obtained using the (M Station) JEOL JMS-700 spectrometer. Some ESI spectra were collected on a Bruker MicroTOF-Q in Glasgow, others by an LTQ Orbitrap XL by the National Mass Spectrometry Service Centre in Swansea. IR Spectra were obtained a Shimadzu 8400S FTIR. Dry solvents Hexylamine was distilled from CaH_2 and stored over KOH. Pyridine was distilled from CaH_2. All glassware for reactions involving anhydrous conditions was flame dried under vacuum, with dry solvents collected from a Puresolv solvent purification system. A CEM Explorer PSL set to a maximum power of 50W, maximum ramp time of 20 min (never reached) and a 15 atm pressure cut off was used for the microwave reactions. Synthetic procedures for compounds **15-18**, **21a-k**, **22a**, **23a-k, 24a**, **25d** and **26c** and are described below. 1-Azido-6-aminohexane,[1] 2-Iodobenzophenone[2] **20** (R^3 = H, R^4 = Ph), 2-iodo-4-nitrobenzophenone[3] **20** (R^3 = NO_2, R^4 = Ph) are known compounds, which were prepared by variations of the literature methods and had corresponding spectroscopic data. Arylboronic acids **19** and all other reagents were purchased from commercial suppliers.

2-Bromo-5-*tert.*-butyl-aniline **15**

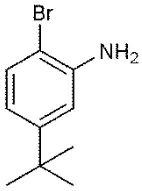

1-Bromo-4-*tert.*-butyl-2-nitrobenzene (3.5 g, 14 mmol, 1.0 eq., prepared in 95% yield from 4-*tert.*-butylbenzene **14** by the literature method[4]) was dissolved in a mixture of AcOH (60 mL) and EtOH (60 mL), and iron fillings (4.06 g, 72.6 mmol, 5.34 eq.) added, then the mixture stirred and heated to 100 ˚C for 2 h under argon. The mixture was cooled then basified with $NaOH_{(aq)}$ (7 M) and extracted with EtOAc (x 2). The combined organics were washed with brine then dried ($MgSO_4$), filtered and the solvent removed under reduced pressure. The brown oil was dried by azeotrope with PhMe then dissolved in Pet. ether and dry loaded onto SiO_2, washed with pet. ether and eluted with Et_2O, then dried ($MgSO_4$), before filtering and removal of the solvent under reduced pressure, giving aniline derivitive **15** as a red oil (2.80 g, 91%). ^{1}H NMR (400 MHz, $CDCl_3$) δ 7.31 (1H, d, *J* = 8.4 Hz, H3), 6.79 (1H, d, *J* = 2.3 Hz, H6), 6.66 (1H, dd, *J* = 8.4, 2.3 Hz, H4), 4.02 (2H, s, br, NH_2), 1.27 [9H, s, $C(CH_3)_3$]. ^{13}C NMR (101 MHz, $CDCl_3$) δ 151.97 (C), 143.59 (C), 132.10 (CH), 117.17 (CH), 113.27 (CH), 106.41 (C), 34.56 (C), 31.31 (CH_3). Consistent with literature data.[4]

1-Bromo-2-fluoro-4-*tert.*-butylbenzene **16**

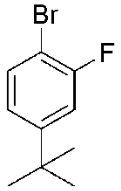

$NOBF_4$ (313 mg, 2.70 mmol, 1.32 eq.) was dissolved in stirring, dry DCM (10.0 mL), at 0 ˚C under argon. After 30 min aniline derivative **15** (471 mg, 2.04 mmol, 1.00 eq.) was added dropwise and the mixture allowed to warm to RT under argon for 13 h. PhMe (10.0 mL) was added then the DCM removed carefully under vacuum. The suspension was heated to reflux for 2 h stirring under argon, then cooled and the solvent removed under reduced pressure. Column chromatography (SiO_2, Pet. ether) gave aryl fluoride **16** as an oil (341

mg, 72%). ^{1}H NMR (500 MHz, $CDCl_3$) δ 7.46 (1H, apparent t, *J* = 7.9 Hz, H6), 7.16 (1H, dd, *J* = 10.9, 2.2 Hz, H3), 7.06 (1H, dd, *J* = 8.4, 2.1 Hz, H5), 1.32 (9H, s, CCH_3). ^{13}C NMR (126 MHz, $CDCl_3$) δ 159.01 (d, *J* = 245.9 Hz, CF), 153.49 (d, *J* = 5.9 Hz, C), 132.95 (CH), 122.49 (d, *J* = 3.2 Hz, CH), 113.94 (d, *J* = 22.4 Hz, CH), 105.51 (d, *J* = 20.8 Hz, C), 34.88 (d, *J* = 1.4 Hz, C) 31.21 (CH_3). ^{19}F NMR (470 MHz, $CDCl_3$) δ −108.22 (dd, 10.8, 7.4 Hz). ν_{Max}(ATR)cm^{-1}: 2963 (C-H), 2870, (C-H), 1566 (C_{Ar}=C_{Ar}), 1481, 1404 (C-F). MS (EI^{+}): 232 (^{81}BrM$^{+\cdot}$, 22%), 230 (^{79}BrM$^{+\cdot}$, 22%), 217 (^{81}BrM$^{+\cdot}$ – $^{\cdot}CH_3$, 77), 215 (^{79}BrM$^{+\cdot}$ – $^{\cdot}CH_3$, 77), 84 (100). HRMS: 232.0078 and 230.0110. $C_{10}H_{12}{}^{81}BrF$ requires 232.0086, $C_{10}H_{12}{}^{79}BrF$ requires 230.0106.

Potassium 2-Fluoro-4-*tert*-butylphenyltrifluoroboronate **17**

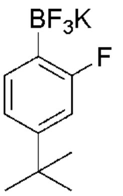

Aryl bromide **16** (5.88 g, 25.5 mmol, 1.00 eq.) was dissolved in dry THF (100 mL) and cooled to –77 ˚C. 1.6 M ^{n}Butyllithium (21.0 mL, 33.6 mmol, 1.32 eq.) was added to the stirring mixture dropwise over 50 min, then the mixture allowed to stir for 90 min. Triisopropylborate (12.0 mL, 50.5 mmol, 1.98 eq.) was added dropwise over 10 min and the mixture allowed to warm to RT and stir for 14 h. The mixture was quenched into a KHF_2 (10.0 g, 128 mmol, 5.0 eq.) solution [MeOH/H_2O (1:1)] and allowed to stir for 55 h then the solvent removed under reduced pressure. The residue was dissolved in acetone and filtered, then the filtrate concentrated. Crystallisation from acetone gave Potassium trifluoroborate salt **17** (4.22 g, 64%). ^{1}H NMR (500 MHz, Acetone) δ 7.45 (1H, apparent t, *J* = 7.3 Hz, H6), 7.03 (1H, ddd, *J* = 7.7, 1.7, 0.9 Hz, H5), 6.87 (1H, dd, *J* = 11.8, 1.6 Hz, H3), 1.31 [9H, s, $C(CH_3)_3$]. ^{13}C NMR (126 MHz, Acetone) δ 168.23 (d, *J* = 235.0 Hz, CF), 152.83 (d, *J* = 7.2 Hz, C), 135.87 (dq, *J* = 13.2, 2.6 Hz, CH), 121.19 (CH), 112.49 (d, *J* = 27.3 Hz, CH), 35.86 (d, *J* = 1.8 Hz, C), 32.71 (CH_3). ^{19}F NMR [377 MHz, $(CD_3)_2CO$] δ −107.63 - −107.79 (m), −137.84 - −138.49 (m). ν_{Max}(ATR)cm^{-1}: 2965 (C-H), 1624, 1551 (C_{Ar}=C_{Ar}), 1398. MS (NSI^{-}): 219 [M^{-} (trifluoroborate anion),

100%], 149 (59). HRMS: 219.0971. $C_{10}H_{12}BF_4$ requires 219.0974. MP: > 250 ˚C.

2-Fluoro-4-nitrophenyl-4,4,5,5-tetramethyl-1,3,2-dioxoborolane **18**

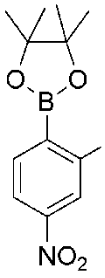

1-Bromo-2-fluoro-4-nitrobenzene (1.88 g, 8.56 mmol, 1.00 eq.), bis(pinacolato)diboron (2.24 g, 8.82 mmol, 1.03 eq.), anhydrous potassium acetate (2.71 g, 27.6 mmol, 3.23 eq.) and (dppf)$PdCl_2$.DCM (150 mg, 0.18 mmol, 2 mol%) were combined and opened to vacuum, then refilled with argon. The cycle was repeated (x 2) then dry 1,4-dioxane (65 mL) added and the reaction heated to 100 ˚C and stirred under argon for 3 h. The mixture was filtered through celite, dried ($MgSO_4$), filtered and the solvent removed under reduced pressure. Column chromatography [SiO_2, gradient from Pet. ether to Pet. ether/EtOAc (6.7:1)] gave impure boronate ester (2.01 g), of which a portion (1.81 g) was chromatographed again [SiO_2, gradient from Pet. ether to Pet. ether/EtOAc (6.7:1)] to give boronate ester **18** as a solid (1.27 g, 61%). ^{1}H NMR (500 MHz, $CDCl_3$) δ 8.00 (1H, dd, *J* = 8.2, 2.0 Hz, H5), 7.92 (1H, dd, *J* = 8.2, 5.8 Hz, H6), 7.88 (1H, dd, *J* = 8.7, 2.0 Hz, H3), 1.38 (12H, s, CCH_3 x 4). ^{13}C NMR (101 MHz, $CDCl_3$) δ 166.69 (d, *J* = 255.5 Hz, CF), 150.99 (d, *J* = 9.2 Hz, C), 137.82 (d, *J* = 8.5 Hz, CH), 118.42 (d, *J* = 3.6 Hz, CH), 110.93 (d, *J* = 29.4 Hz, CH), 84.84 (C), 24.88 (CH_3). ^{19}F NMR (377 MHz, $CDCl_3$) δ −98.70 (dd, *J* = 8.6, 5.8 Hz). ν_{Max}(ATR)cm^{-1}: 2978 (C-H), 2932 (C-H), 1611 (C_{Ar}=C_{Ar}), 1524 (NO_2), 1341 (NO_2). MS (APCI$^+$): 268 [$(M+H)^+$, 100%]. HRMS: 268.1149. $C_{12}H_{16}BFNO_4$ requires 268.1151. MP: 73 - 77 ˚C.

2-Bromo-5-nitroacetophenone **20** (R^3 = NO_2, R^4 = Me) , but bromo rather than iodo

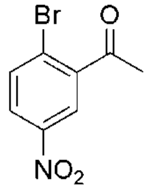

2-Bromoacetophenone (2.00 g, 10.1 mmol, 1.00 eq.) was added to a solution of KNO_3 (1.26 g, 12.5 mmol, 1.23 eq.) in $H_2SO_{4(aq)}$ (10 mL) stirring at 0 ˚C. The mixture was allowed to stir warming to RT for 70 min, and was then quenched (H_2O) and extracted with DCM (x 2). The combined organics were dried ($MgSO_4$), filtered and the solvent removed under reduced pressure. Column chromatography [SiO_2, Pet. ether/EtOAc (7:1)] gave 2-bromo-5-nitroacetophenone as a solid (1.34 g, 55%). R_f = 0.34 in Pet. ether/EtOAc (7:1). ^1H NMR (500 MHz, $CDCl_3$) δ 8.28 (1H, d, *J* = 2.7 Hz, H6), 8.12 (1H, dd, *J* = 8.7, 2.7 Hz, H4), 7.82 (1H, d, *J* = 8.7 Hz, H3), 2.67 (3H, s, $COCH_3$). ^{13}C NMR (101 MHz, $CDCl_3$) δ 198.87 (C), 142.39 (C), 135.27 (CH), 126.22 (C), 125.98 (CH), 123.83 (CH), 30.19 (CH_3). ν_{Max}(ATR)cm^{-1}: 3095 (C_{Ar}-H), 3073 (C_{Ar}-H), 1709 (C=O), 1604 (C_{Ar}=C_{Ar}), 1568 (C_{Ar}=C_{Ar}), 1523 (NO_2), 1355 (NO_2). MS (EI^+): 245 ($^{81}BrM^{+\cdot}$, 22%), 243 ($^{79}BrM^{+\cdot}$, 26), 230 ($^{81}BrM^{+\cdot}$ – $^{\cdot}CH_3$, 82), 228 ($^{79}BrM^{+\cdot}$ – $^{\cdot}CH_3$, 100). HRMS: 244.9514. $C_8H_6{}^{81}BrNO_3$ requires 244.9511. MP: 85 - 87 ˚C. ^1H NMR data agreed with lit.[5]

2-(2''-Fluoro-4''-*tert*-butylphenyl)-5-nitrobenzophenone **21a**

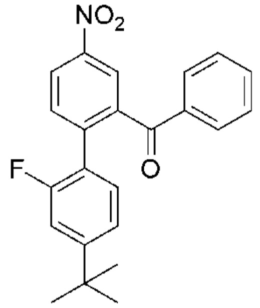

2-Iodo-5-nitrobenzophenone (431 mg, 1.22 mmol, 1.00 eq.), PEPPSI-iPr catalyst (51 mg, 0.08 mmol, 0.06 eq.) and potassium trifluoroborate salt **17** (353 mg, 1.37 mmol, 1.12 eq.) were combined under argon then the reaction vessel was opened to vacuum, then refilled with argon. This process was repeated twice then *N,N*-diisopropylethylamine (0.74 mL, 4.2 mmol, 3.4 eq.)

and degassed ethanol (10.0 mL) added. The mixture was heated to reflux stirring for 4 h. After cooling the mixture was partitioned between Et_2O and H_2O, then the aqueous re-extracted with Et_2O. The combined organics were washed with brine, then dried ($MgSO_4$), filtered through celite eluting with Et_2O and the solvent removed under reduced pressure. Column chromatography [SiO_2, Pet. Ether/EtOAc (8:1), loaded in $CHCl_3$] gave ketone **21a** as a pale straw coloured oil (357 mg, 78%). R_f = 0.26 in Pet. ether/EtOAc (5:1). ^{1}H NMR (500 MHz, $CDCl_3$) δ 8.44 - 8.39 (2H, m, H4, H6), 7.67 (3H, d, br, *J* = 7.8 Hz, H3, H2', H6'), 7.49 (1H, apparent t, *J* = 7.4 Hz, H4'), 7.35 (2H, apparent t, *J* = 7.7 Hz, H3', H5'), 7.18 (1H, apparent t, *J* = 8.1 Hz, H6''), 7.11 (1H, dd, *J* = 8.1, 1.8 Hz, H5''), 6.92 (1H, dd, *J* = 12.4, 1.7 Hz, H3''), 1.23 [9H, s, $C(CH_3)_3$]. ^{13}C NMR (126 MHz, $CDCl_3$) δ 195.21 (d, *J* = 1.1 Hz, C), 158.66 (d, *J* = 246.7 Hz, CF), 155.27 (d, *J* = 6.9 Hz, C), 146.85 (C), 141.83 (C), 140.31 (C), 136.24 (C), 133.31 (CH), 132.33 (d, *J* = 1.2 Hz, CH), 130.55 (d, *J* = 3.1 Hz, CH), 129.79 (CH), 128.33 (CH), 124.98 (CH), 124.11 (CH), 122.73 (d, J = 15.5 Hz, C), 121.43 (d, *J* = 3.0 Hz, CH), 112.80 (d, *J* = 22.4 Hz, CH), 34.82 (d, *J* = 1.4 Hz, C), 30.94 (CH_3). ^{19}F NMR (470 MHz, $CDCl_3$) δ −115.57 - −115.66 (m). ν_{Max}(ATR)cm^{-1}: 2964 (CH), 1670 (C=O), 1606 (C_{Ar}=C_{Ar}), 1582 (C_{Ar}=C_{Ar}), 1523 (NO_2), 1349 (NO_2). MS (EI$^+$): 377 ($M^{+\cdot}$, 64%), 362 ($M^{+\cdot}$ – $^\cdot CH_3$, 100). HRMS (EI$^+$): 377.1426. $C_{23}H_{20}FNO_3$ requires $M^{+\cdot}$, 377.1427.

2-(2''-Fluorophenyl)-5-nitrobenzophenone **21b**

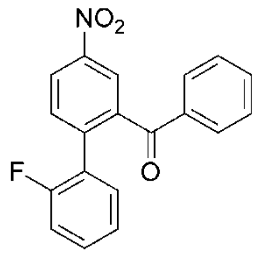

2-Iodo-5-nitrobenzophenone (1.29 g, 3.65 mmol, 1.00 eq.), PEPPSI-iPr catalyst (72 mg, 0.11 mmol, 0.03 eq.) and 2-fluorophenylboronic acid (450 mg, 3.65 mmol, 1.00 eq.) were combined under argon and the reaction vessel was opened to vacuum, then refilled with argon. This process was repeated twice then *N,N*-diisopropylethylamine (1.90 mL, 10.9 mmol, 2.99 eq.) and degassed ethanol (18.0 mL) added. The mixture was heated to reflux stirring for 16 h. After cooling the mixture was filtered through celite eluting with DCM

and the solvent removed under reduced pressure. After column chromatography [SiO_2, Pet. ether/Et_2O gradient (99:1) to (9:1)]. The solvent was removed under reduced pressure and the material suspended in Pet. ether/Et_2O (9:1) then sonicated. Ketone **21b** precipitated as an amorphous solid (570 mg, 49%). ^{1}H NMR (400 MHz, $CDCl_3$) δ 8.44 (1H, dd, *J* = 8.4, 2.4 Hz, H4), 8.41 (1H, dd, *J* = 2.4, 0.5 Hz, H6), 7.71 - 7.67 (3H, m, H3, H2', H6'), 7.52 (1H, ddt, *J* = 8.0, 6.9, 1.3 Hz, H4'), 7.40 - 7.35 (2H, m, H3', H5'), 7.30 - 7.23 (2H, m, H4'', H6''), 7.11 (1H, td, *J* = 7,8, 1.1 Hz, H5''), 6.95 (1H, ddd, J = 10.2, 8.9, 1.0 Hz, m, H3''). ^{13}C NMR (101 MHz, $CDCl_3$) δ 195.11 (d, *J* = 1.1 Hz, C), 158.91 (d, *J* = 247.8 Hz, CF), 147.18 (C), 141.69 (C), 140.57 (d, *J* = 1.0 Hz, C), 136.19 (C), 133.68 (CH), 132.53 (d, *J* = 1.4 Hz, CH), 131.18 (d, *J* = 2.6 Hz, CH), 130.99 (d, *J* = 8.3 Hz, CH), 129.99 (CH), 128.58 (CH), 126.05 (d, *J* = 14.8 Hz, C), 125.09 (CH), 124.60 (d, *J* = 3.7 Hz, CH), 124.15 (CH), 115.90 (d, *J* = 21.8 Hz, CH). ^{19}F NMR (377 MHz, $CDCl_3$) δ −115.11 - −115.20 (m). ν_{Max}(ATR)cm^{-1}: 3067 (C_{Ar}-H), 1667 (C=O), 1597 (C_{Ar}=C_{Ar}), 1582 (C_{Ar}=C_{Ar}), 1522 (NO_2), 1348 (NO_2). MS (EI$^+$): 321 ($M^{+\cdot}$, 39), 227 ($M^{+\cdot}$ – C_6H_5F, 37), 105 ($PhCO^+$, 100), 77 (Ph^+, 39). MP: 108 - 110 ˚C. HRMS: 321.0807. $C_{19}H_{12}NO_3F$ requires $M^{+\cdot}$, 321.0801.

2-(2''-Fluoro-4''-bromophenyl)-5-nitrobenzophenone **21c**

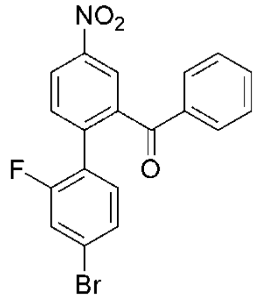

2-Iodo-5-nitrobenzophenone (1.45 g, 4.11 mmol, 1.00 eq.), PEPPSI-iPr catalyst (94 mg, 0.14 mmol, 0.03 eq.) and 4-bromo-2-fluorophenylboronic acid (1.00 g, 4.57 mmol, 1.11 eq.) were combined under argon and the reaction vessel was opened to vacuum, then refilled with argon. This process was repeated twice then *N,N*-diisopropylethylamine (2.4 mL, 14 mmol, 3.4 eq.) and degassed ethanol (40 mL) added. The mixture was heated to reflux stirring for 22 h. After cooling the mixture was filtered through celite eluting with DCM and the solvent removed under reduced pressure. The residue was

partitioned between DCM and 1 M $HCl_{(aq)}$ then the aqueous layer further extracted (DCM x 2). The combined organics were dried ($MgSO_4$), filtered and the solvent removed under reduced pressure. Column chromatography [SiO_2, Pet. ether/EtOAc (15:1)]. Gave ketone **21c** as a yellow solid (896 mg, 55%). R_f = 0.32 in Pet. ether/EtOAc (5:1). ^{1}H NMR (400 MHz, $CDCl_3$) δ 8.43 (1H, dd, *J* = 8.4, 2.4 Hz, H4), 8.39 (1H, d, *J* = 2.3 Hz, H6), 7.75 - 7.68 (2H, m, H2', H6'), 7.65 (1H, d, *J* = 8.4 Hz, H3), 7.57 (1H, ddt, *J* = 8.0, 7.0, 1.3 Hz, H4'), 7.41 (2H, t, *J* = 7.7 Hz, H3', H5'), 7.28 (1H, dd, *J* = 8.3, 1.6 Hz, H5''), 7.19 - 7.12 (2H, m, H3'', H6''). ^{13}C NMR (101 MHz, $CDCl_3$) δ 194.82 (d, *J* = 0.7 Hz, C), 158.56 (d, *J* = 252.3 Hz, CF), 147.20 (C), 140.69 (C), 140.36 (C), 135.94 (C), 133.96 (CH), 132.42 (d, *J* = 0.6 Hz, CH), 132.02 (d, *J* = 3.3 Hz, CH), 130.06 (CH), 128.74 (CH), 128.07 (d, *J* = 3.7 Hz, CH), 125.29 (CH), 125.21 (d, *J* = 15.1 Hz, C), 124.21 (CH), 123.64 (d, *J* = 9.5 Hz, C), 119.62 (d, *J* = 25.1 Hz, CH). ^{19}F NMR (470 MHz, $CDCl_3$) δ −112.25 (broad apparent t, *J* = 8.7 Hz). ν_{Max}(ATR)cm^{-1}: 3088 (C_{Ar}-H), 3071 (C_{Ar}-H), 1668 (C=O), 1605 (C_{Ar}=C_{Ar}), 1582 (C_{Ar}=C_{Ar}), 1524 (NO_2), 1350 (NO_2). MS (EI$^+$): 401 (^{81}BrM$^{+\cdot}$, 26%) 399 (^{79}BrM$^{+\cdot}$, 30), 353 (M$^{+\cdot}$ – $NO_2^{\cdot}$, 30), 105 (PhCO$^+$, 100), 77 (Ph$^+$, 34). HRMS: 398.9902 and 400.9877. $C_{19}H_{11}{}^{79}BrFNO_3$ requires M$^{+\cdot}$, 398.9906 and $C_{19}H_{11}{}^{81}BrFNO_3$ requires M$^{+\cdot}$, 400.9888. MP: 101 -105 ˚C.

<u>2-(2''-Fluorophenyl)benzophenone **21d**</u>

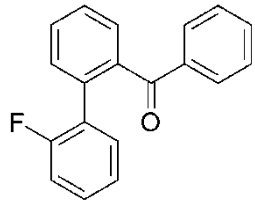

2-Iodobenzophenone (1.41 g, 4.57 mmol, 1.00 eq.), PEPPSI-iPr catalyst (76 mg, 0.11 mmol, 0.03 eq.) and 2-fluorophenylboronic acid (563 mg, 4.55 mmol, 1.00 eq.) were combined under argon and the reaction vessel was opened to vacuum, then refilled with argon. This process was repeated twice then *N,N*-diisopropylethylamine (1.91 mL, 11.0 mmol, 2.75 eq.) and degassed ethanol (18.0 mL) added. The mixture was heated to reflux stirring for 16 h. After cooling the mixture was filtered through celite eluting with DCM and the solvent removed under reduced pressure. The residue was taken up in Et_2O then dried ($MgSO_4$), filtered and the solvent removed under reduced pressure.

Column chromatography [SiO_2, Pet. ether/EtOAc (12:1)] gave ketone **21d** as a clear oil (958 mg, 76%). R_f = 0.24 Pet. ether/EtOAc (12:1). ^{1}H NMR (500 MHz, $CDCl_3$) δ 7.71 - 7.67 (2H, m, H2', H6'), 7.62 - 7.55 (2H, m, H6, H4), 7.51 - 7.47 (2H, m, H3, H5), 7.43 (1H, ddt, *J* 7.9, 6.8, 1.3 Hz, H4'), 7.35 - 7.29 (2H, t, *J* = 7.7 Hz, H3', H5'), 7.24 (apparent td, *J* = 7.6, 1.8 Hz, H6''), 7.17 - 7.13 (1H, m, H4''), 7.05 (1H, apparent td, *J* = 7.5, 1.3 Hz, H5''), 6.88 (1H, ddd, *J* = 10.1, 8.4, 1.2 Hz, H3''). ^{13}C NMR (101 MHz, $CDCl_3$) δ 197.70 (d, *J* = 1.1 Hz, C), 159.20 (d, *J* = 246.3 Hz, CF), 139.34 (d, *J* = 0.8 Hz, C), 137.49 (C), 135.39 (C), 132.83 (CH), 131.60 (d, *J* = 3.2 Hz, CH), 131.24 (d, *J* = 1.2 Hz, CH), 130.65 (CH), 130.06 (CH), 129.59 (d, *J* = 8.1 Hz, CH), 129.32 (CH), 128.17 (CH), 128.10 (d, *J* = 15.1 Hz, C), 127.66 (CH), 124.19 (d, *J* = 3.7 Hz, CH), 115.53 (d, *J* = 22.3 Hz, CH). ^{19}F NMR (470 MHz, $CDCl_3$) δ −115.75 (ddd, *J* = 10.0, 7.7, 5.2 Hz). ν_{Max}(ATR)cm^{-1}: 3061 (C_{Ar}-H), 3030 (C_{Ar}-H), 1663 (C=O), 1595 (C_{Ar}=C_{Ar}), 1582 (C_{Ar}=C_{Ar}), 1501 (C_{Ar}=C_{Ar}). MS (EI$^+$): 276 ($M^{+\cdot}$, 89%), 257 (M^+ – $F^\cdot$, 38), 199 ($M^{+\cdot}$ – $Ph^\cdot$, 100), 170 ($C_{12}H_{10}O^{+\cdot}$, 62). HRMS: 276.0946. $C_{19}H_{13}FO$ requires $M^{+\cdot}$, 276.0950.

<u>2-(2''-Fluoro-5''-methoxyphenyl)-5-nitrobenzophenone **21e**</u>

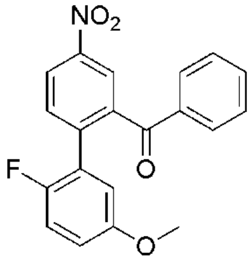

2-Iodo-5-nitrobenzophenone (625 mg, 1.77 mmol, 1.00 eq.), PEPPSI-iPr catalyst (36 mg, 0.05 mmol, 0.03 eq.) and 2-fluoro-5-methoxyphenylboronic acid (301 mg, 1.77 mmol, 1.00 eq.) were combined under argon then the reaction vessel was opened to vacuum, then refilled with argon. This process was repeated twice then *N,N*-diisopropylethylamine (0.93 mL, 5.3 mmol, 3.0 eq.) and degassed ethanol (12.0 mL) added. The mixture was heated to reflux stirring for 22 h. After cooling the mixture was filtered through celite eluting with DCM and the solvent removed under reduced pressure. The material was the redissolved in Et_2O, dried with $MgSO_4$ and ethereal HCl (2 M) added to precipitate the diisopropylethylamine as a salt, which was then removed by

filtration. The solvent was then removed from the filtrate under reduced pressure and the solid recrystallised from $CHCl_3$ then washed with $CHCl_3$ to give ketone **21e** as a brown solid (517 mg, 83%). R_f = 0.20 [Pet. ether/EtOAc (9:1)]. ^{1}H NMR (500 MHz, $CDCl_3$) δ 8.43 (1H, dd, *J* = 8.4, 2.4 Hz, H4), 8.40 (1H, d, *J* = 2.3 Hz, H6), 7.70 (3H, apparent t, *J* = 8.7 Hz, H3, H2', H6'), 7.53 (1H, tt, *J* = 7.4, 1.3 Hz, H4'), 7.39 (2H, apparent t, *J* = 7.8 Hz, H3', H5'), 6.89 - 6.84 (1H, m, H3''), 6.78 - 6.74 (2H, m, H4'', H6''), 3.72 (s, 3H). ^{13}C NMR (101 MHz, $CDCl_3$) δ 195.13 (C), 155.80 (d, *J* = 2.0 Hz, C), 153.25 (d, *J* = 240.3 Hz, CF), 147.13 (C), 141.66 (C), 140.54 (C), 136.14 (C), 133.74 (CH), 132.46 (d, *J* = 1.0 Hz, CH), 130.00 (CH), 128.60 (CH), 126.32 (d, *J* = 16.5 Hz, C),125.12 (CH), 124.10 (CH), 116.58 (d, *J* = 23.9 Hz, CH), 116.13 (d, *J* = 8.0 Hz, CH), 115.70 (d, *J* = 2.5 Hz, CH), 55.93 (CH_3). ^{9}F NMR (377 MHz, $CDCl_3$) δ −126.33 (dt, *J* = 9.6, 4.8 Hz). ν_{Max}(ATR)cm^{-1}: 2951 (CH), 2934 (CH), 2845 (CH), 1672 (C=O), 1595 ($C=C_{Ar}$), 1578 ($C=C_{Ar}$), 1518 ($C=C_{Ar}$), 1499 (NO_2), 1346 (NO_2). MS (EI^+): 351 ($M^{+\cdot}$, 90%), 279 ($M^{+\cdot}$ – NO_2 – C_2H_2, 30), 274 ($M^{+\cdot}$ – $Ph^{\cdot}$, 25), 227 ($PhCOC_6H_4NO_2^{+\cdot}$, 25), 167,149 ($O_2NC_6H_3CO^{+\cdot}$, 100) 105 ($PhCO^+$, 97), 77 (Ph^+, 45). HRMS: 351.0902. $C_{20}H_{14}FNO_4$ requires $M^{+\cdot}$, 351.0907. MP: 100 - 102 ˚C.

<u>2-(2''-Fluoro-4''-nitrophenyl)-5-nitrobenzophenone **21f**</u>

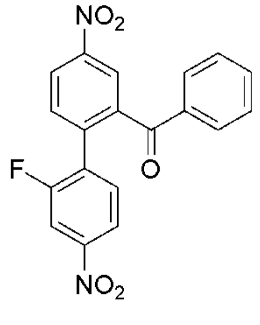

2-Iodo-5-nitrobenzophenone (995 mg, 2.82 mmol, 1.07 eq.), PEPPSI-iPr catalyst (105 mg, 0.15 mmol, 0.05 eq.) and arylboronate **18** (706 mg, 2.64 mmol, 1.00 eq.) were combined, then divided into three microwave tubes (10 mL) and each reaction vessel was opened to vacuum, then refilled with argon. This process was repeated twice then *N,N*-diisopropylethylamine (1.56 mL, 8.96 mmol, 3.18 eq.) and degassed ethanol (15.0 mL) divided between them. Each tube was sealed and heated to 100 ˚C for 30 min in a microwave. After cooling the material was combined in DCM and filtered through a celite/K_2CO_3

mixture and the solvent removed from the filtrate under reduced pressure. The material was dry loaded onto celite in DCM then column chromatography [SiO_2, Pet. Ether/DCM, (1:1)] gave ketone **21f** as a solid (962 mg, 99%). R_f = 0.14 DCM/Pet. ether (6:4). ^{1}H NMR (500 MHz, $CDCl_3$) δ 8.50 (1H, dd, *J* = 8.4, 2.4 Hz, H4), 8.44 (1H, d, *J* = 2.3 Hz, H6), 8.07 (1H, dd, *J* = 8.4, 2.1 Hz, H5''), 7.87 (1H, dd, *J* = 9.4, 2.2 Hz, H3''), 7.74 (2H, d, *J* = 8.5 Hz, H2', H6'), 7.69 (d, *J* = 8.4 Hz, H3), 7.63 - 7.58 (1H, tt, *J* = 7.5, 1.2 Hz, H4'), 7.52 (1H, dd, *J* = 8.4, 7.4 Hz, H6''), 7.46 (2H, m, H3', H5'). ^{13}C NMR (126 MHz, $CDCl_3$) δ 194.07 (d, *J* = 1.1 Hz, C), 158.42 (d, *J* = 252.3 Hz, CF), 148.95 (d, *J* = 8.6 Hz, C), 147.76 (C), 140.49 (d, *J* = 0.9 Hz, C), 139.37 (C), 135.77 (C), 134.02 (CH), 132.86 (d, *J* = 15.6 Hz, C), 132.22 (d, *J* = 1.3 Hz, CH), 131.74 (d, *J* = 3.2 Hz, CH), 129.92 (CH), 128.74 (CH), 125.30 (CH), 124.24 (CH), 119.47 (d, *J* = 3.8 Hz, CH), 111.62 (d, *J* = 27.4 Hz, CH). ^{19}F NMR (470 MHz, $CDCl_3$) δ −110.18 (dd, *J* = 9.3, 7.7 Hz). ν_{Max}(ATR)cm^{-1}: 3090 (C_{Ar}-H), 1667 (C=O), 1597 (C_{Ar}=C_{Ar}), 1522 (NO_2), 1346 (NO_2). MS (CI^+): 367 [$(M+H)^+$, 100%], 228 [$(PhCOC_6H_4NO_2+H)^+$, 92]. HRMS: 367.0722. $C_{19}H_{12}FN_2O_5$ requires $(M+H)^+$, 367.0730. MP: 143 -144 ˚C.

2-(2''-Fluoro-4''-nitro-5''-methoxyphenyl)-5-nitrobenzophenone **21g**

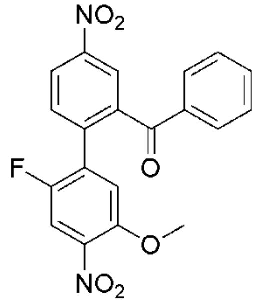

Benzophenone **21e** (749 mg, 2.13 mmol, 1.00 eq.), acetic anhydride (20 mL) and DCM (10.0 mL) were combined and cooled to 0 ˚C. Concentrated nitric acid (2.0 mL, 44 mmol, 21 eq.) was added and the mixture allowed to stir for 2 h then quenched into $NaOH_{(aq)}$ (1 M) and extracted (DCM, x 2). The organic layer was dried ($MgSO_4$), filtered and the solvent removed under reduced pressure. The residue was then dissolved in minimal hot EtOAc and precipitated by addition of pet. ether, then left to settle (12 h). The solvent was decanted off and the solid triturated with hot EtOAc. Nitrated benzophenone **21g** was isolated as a brown solid (604 mg, 72%). ^{1}H NMR (400 MHz, $CDCl_3$)

δ 8.48 (1H, dd, *J* = 8.4, 2.4 Hz, H4), 8.42 (1H, d, *J* = 2.3 Hz, H6), 7.76 - 7.68 (3H, m, H3, H2', H6'), 7.60 (1H, ddt, *J* = 8.0, 7.0, 1.3 Hz, H4'), 7.55 (1H, d, *J* = 8.8 Hz, H3''), 7.49 - 7.41 (2H, m, H3', H5'), 7.00 (1H, d, *J* = 5.9 Hz, H6''), 3.89 (s, 3H). ^{13}C NMR (101 MHz, $CDCl_3$) δ 194.56 (C), 151.45 (d, *J* = 244.8 Hz, CF), 149.60 (d, *J* = 2.7 Hz, C), 147.75 (C), 140.75 (C), 139.48 (d, *J* = 0.9 Hz, C), 139.06 (d, *J* = 8.1 Hz, C), 135.78 (C), 134.37 (CH), 132.33 (d, *J* = 1.3 Hz, CH), 132.01 (d, *J* = 16.7 Hz, C), 130.09 (CH), 128.98 (CH), 125.40 (CH), 124.18 (CH), 116.20 (d, *J* = 2.5 Hz, CH), 113.66 (d, *J* = 28.3 Hz, CH), 57.29 (CH_3). ^{19}F NMR (377 MHz, $CDCl_3$) δ −122.91 (dd, *J* = 8.8, 6.0 Hz). ν_{Max}(ATR)cm^{-1}: 3069 (C_{Ar}-H), 1668 (C=O), 1595 (C_{Ar}=C_{Ar}), 1522 (NO_2), 1346 (NO_2). MS (EI$^+$): 396 ($M^{+\cdot}$, 100%), 105 ($PhCO^+$, 59), 77 (Ph^+, 31). HRMS: 396.0751. $C_{20}H_{13}FN_2O_6$ requires $M^{+\cdot}$, 396.0758. MP: 143 - 144 ˚C.

2-(2'-Fluoro-4'-nitrophenyl)-5-nitroacetophenone **21h**

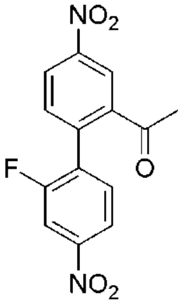

2-Bromo-5-nitroacetophenone (1.00 g, 4.10 mmol, 1.00 eq.), PEPPSI-iPr catalyst (140 mg, 0.21 mmol, 0.05 eq.) and arylboronic acid **18** (1.13 mg, 4.22 mmol, 1.03 eq.) were divided evenly between two microwave tubes (10 mL) under argon. The tubes were opened to vacuum, then refilled with argon. This process was repeated twice then *N,N*-diisopropylethylamine (2.00 mL, 11.4 mmol, 2.79 eq.) and degassed ethanol (10.0 mL) were split between the tubes. Each tube was heated to 125 ˚C for 10 min. After cooling the mixtures were filtered through celite eluting with DCM and the solvent removed under reduced pressure. The residue was redissolved then partitioned (DCM/1 M $HCl_{(aq)}$) then the aqueous layer extracted (DCM x 2). The combined organics were dried ($MgSO_4$), filtered and the solvent removed under reduced pressure. Additional boronic acid (200 mg, 0.75 mmol, 0.18 eq.), *N,N*-diisopropylethylamine (1.00 mL, 5.74 mmol, 1.40 eq.), PEPPSI-iPr catalyst (50 mg, 0.07 mmol, 0.02 eq.) and ethanol (10.0 mL) was added and the

mixture divided then the previous processes repeated. Column chromatography [SiO_2, Pet. Ether/ EtOAc (5:1)] gave ketone **21h** as a solid (499 mg, 40%). R_f = 0.23 in Pet. Ether/ EtOAc (5:1). ^{1}H NMR (500 MHz, $CDCl_3$) δ 8.65 (1H, d, *J* = 2.3 Hz, H6), 8.46 (1H, dd, *J* = 8.4, 2.3 Hz, H4), 8.17 (1H, dd, *J* = 8.4, 1.9 Hz, H5'), 7.99 (1H, dd, *J* = 9.5, 2.2 Hz, H3'), 7.56 (1H, d, *J* = 8.4 Hz, H3), 7.51 (dd, *J* = 8.2, 7.4 Hz, H6'), 2.62 (s, 3H). ^{13}C NMR (126 MHz, $CDCl_3$) δ 198.04 (d, *J* = 0.6 Hz, C), 158.38 (d, *J* = 251.0 Hz, CF), 148.90 (d, *J* = 8.7 Hz, C), 148.12 (C), 139.95 (C), 138.97 (C), 134.19 (d, *J* = 16.0 Hz, C), 132.89 (CH), 131.01 (d, *J* = 3.3 Hz, CH), 126.41 (CH), 123.64 (CH), 120.03 (d, *J* = 3.7 Hz, CH), 111.65 (d, *J* = 27.4 Hz, CH), 28.44 (CH_3). ^{19}F NMR (470 MHz, $CDCl_3$) δ −111.05 (dd, *J* = 9.6, 7.2 Hz). ν_{Max}(ATR)cm^{-1}: 3111 (C-H), 3078, (C-H), 3057 (C-H), 1699 (C=O), 1609 (C_{Ar}=C_{Ar}), 1580 (C_{Ar}=C_{Ar}), 1510 (NO_2), 1346 (NO_2). MS (EI^+): 304 ($M^{+\cdot}$, 62%), 289 ($M^{+\cdot}$ – $^{\cdot}CH_3$, 100), 285 ($M^{+\cdot}$ – $^{\cdot}F$, 9), 243 ($M^{+\cdot}$ – $F^{\cdot}$ – CH_2CO, 22) 213 ($C_{14}H_{10}FO^+$, 15). HRMS: 304.0491. $C_{14}H_9FN_2O_5$ requires $M^{+\cdot}$, 304.0495. MP: 123 - 124 °C.

2-(2''-Chloro-5''-nitrophenyl)-5-nitrobenzophenone **21i**

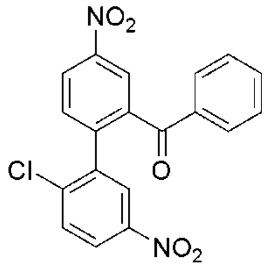

2-Iodo-5-nitrobenzophenone (1.00 g, 2.83 mmol, 1.12 eq.), PEPPSI-iPr catalyst (96 mg, 0.14 mmol, 0.05 eq.) and 2-chloro-5-nitrophenylboronic acid (510 mg, 2.53 mmol, 1.00 eq.) were divided evenly between two microwave tubes (10.0 mL) under argon. The tubes were opened to vacuum, then refilled with argon. This process was repeated twice then *N,N*-diisopropylethylamine (1.45 mL, 8.32 mmol, 3.29 eq.) and degassed ethanol (10.0 mL) were split between the tubes. Each tube was heated to 125 °C for 10 min. After cooling the mixtures were combined and partitioned between DCM and 1 M $HCl_{(aq)}$ then the aqueous layer extracted (DCM x 2). The combined organics were dried ($MgSO_4$), filtered and the solvent removed under reduced pressure. Column chromatography [SiO_2, Pet. Ether/EtOAc

(15:1), after drying loading onto celite]. Ketone **21i** was isolated as a solid (540 mg, 56%). ^{1}H NMR (500 MHz, $CDCl_3$) δ 8.50 (1H, dd, *J* = 8.4, 2.4 Hz, H4), 8.48 (1H, d, *J* = 2.3 Hz, H6), 8.16 (1H, d, *J* = 2.6 Hz, H6''), 8.13 (1H, dd, *J* = 8.7, 2.7 Hz, H4''), 7.72 (2H, dd, *J* = 8.5, 1.4 Hz, H2', H6'), 7.64 (1H, dd, *J* = 8.4, 0.5 Hz, H3), 7.59 (1H, ddt, *J* = 7.8, 7.1, 1.3 Hz, H4'), 7.52 (1H, d, *J* = 8.7 Hz, H3''), 7.37 (2H, t, br, *J* = 7.9 Hz, H3', H5'). ^{13}C NMR (126 MHz, $CDCl_3$) δ 194.03 (C), 147.63 (C), 146.45 (C), 143.25 (C), 140.09 (C), 139.32 (C), 138.79 (C), 135.84 (C), 134.11 (CH), 132.60 (CH), 130.80 (CH), 130.15 (CH), 128.89 (CH), 126.11 (CH), 125.66 (CH), 124.75 (CH), 124.62 (CH). ν_{Max}(ATR)cm^{-1}: 3057 (C_{Ar}-H), 1670 (C=O), 1597 (C_{Ar}=C_{Ar}), 1574 (C_{Ar}=C_{Ar}), 1530 (NO_2), 1348 (NO_2). MS (EI$^+$): 385 [(^{37}ClM+H)$^+$, 35%], 383 [(^{35}ClM+H)$^+$, 100]. HRMS: 385.0416 and 383.0432. $C_{19}H_{12}{}^{37}ClN_2O_5$ requires M+H$^+$, 385.0413 and $C_{19}H_{12}{}^{35}ClN_2O_5$ requires M+H$^+$, 383.0435. MP: 115 - 116 °C.

2-(2''-Fluoro-3''-pyridyl)-5-nitrobenzophenone **21j**

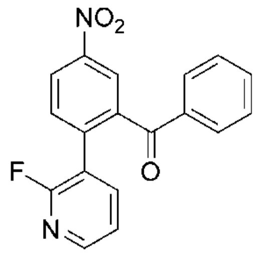

2-Iodo-5-nitrobenzophenone (1.00 g, 2.83 mmol, 1.00 eq.), PEPPSI-iPr catalyst (100 mg, 0.15 mmol, 0.05 eq.) and 2-fluoro-3-pyridylboronic acid (400 mg, 2.84 mmol, 1.00 eq.) were divided evenly between two microwave tubes (10 mL) under argon. The tubes were then opened to vacuum, then refilled with argon. This process was repeated twice then *N,N*-diisopropylethylamine (1.46 mL, 8.38 mmol, 2.96 eq.) and degassed ethanol (10 mL) were split between the tubes. Each tube was heated to 125 °C for 1 h in a microwave. After cooling the mixtures were combined and filtered through celite with DCM and the solvent removed under reduced pressure. The residue was partitioned between DCM and 1 M $HCl_{(aq)}$ then the aqueous layer extracted (DCM x 2). The combined organics were dried ($MgSO_4$), filtered and the solvent removed under reduced pressure. Column chromatography (SiO_2, Pet. Ether/EtOAc gradient from 7:1 to 5:1). The product **21j** was isolated as a solid (480 mg, 53%). R_f = 0.14 in Pet ether/ EtOAc (5:1). ^{1}H NMR (400 MHz,

$CDCl_3$) δ 8.46 (1H, dd, *J* = 8.4, 2.4 Hz, H4), 8.42 (1H, d, *J* = 2.2 Hz, H6), 8.15 (1H, ddd, *J* = 4.9, 1.9, 1.2 Hz, H4''), 7.77 - 7.65 (4H, m, H3, H6'', H2', H6'), 7.56 (1H, ddt, *J* = 8.0, 6.8, 1.3 Hz, H4'), 7.45 - 7.38 (2H, m, H3', H5'), 7.20 (1H, ddd, *J* = 7.4, 4.9, 1.8 Hz, H5''). ^{13}C NMR (101 MHz, $CDCl_3$) δ 194.67 (C), 159.30 (d, *J* = 238.7 Hz, CF), 148.26 (d, *J* = 14.8 Hz, CH), 147.43 (C), 141.47 (d, *J* = 3.5 Hz, CH), 140.45 (C), 139.85 (d, *J* = 4.5 Hz, C), 135.85 (C), 134.05 (CH), 132.44 (d, *J* = 0.8 Hz, CH), 129.99 (CH), 128.80 (CH), 125.35 (CH), 124.29 (CH), 121.73 (d, *J* = 4.4 Hz, CH), 121.01 (d, *J* = 30.4 Hz, C). ^{19}F NMR (377 MHz, $CDCl_3$) δ −68.76 (broad d, *J* = 9.5 Hz). ν_{Max}(ATR)cm^{-1}: 3069 (C_{Ar}-H), 3062 (C_{Ar}-H), 1668 (C=O), 1607 (C_{Ar}=C_{Ar}), 1583 (C_{Ar}=C_{Ar}), 1523 (NO_2), 1351 (NO_2). MS (EI^+): 322 ($M^{+\cdot}$, 100%), 245 (M^+ – $Ph^\cdot$, 20), 121 ($C_6H_3NO_2^{+\cdot}$, 40), 105 ($PhCO^+$, 87), 77 (Ph^+, 37). HRMS: 322.0750. $C_{18}H_{11}FN_2O_3$ requires $M^{+\cdot}$, 322.0754. MP: 125 -127 °C.

<u>2-(2''-Fluoro-3''-methylphenyl)-5-nitrobenzophenone **21k**</u>

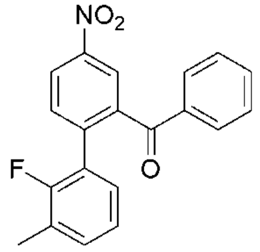

2-Iodo-5-nitrobenzophenone (688 mg, 1.95 mmol, 1.00 eq.), PEPPSI-iPr catalyst (41 mg, 0.06 mmol, 0.03 eq.) and 2-fluoro-3methylphenylboronic acid (300 mg, 1.95 mmol, 1.00 eq.) were combined under argon then the reaction vessel was opened to vacuum, then refilled with argon. This process was repeated twice then *N,N*-diisopropylethylamine (1.02 mL, 5.85 mmol, 3.00 eq.) and degassed ethanol (12.0 mL) added. The mixture was heated to reflux stirring for 22 h. After cooling the mixture was filtered through celite eluting with DCM and the solvent removed under reduced pressure. The material was the redissolved in Et_2O, dried with $MgSO_4$ and ethereal HCl (2 M) added to precipitate the diisopropylethylamine as a salt, which was then removed by filtration. The solvent was then removed from the filtrate under reduced pressure and the solid recrystallised from $CHCl_3$ and washed with $CHCl_3$ to give ketone **21k** as a brown solid (430 mg, 66%). R_f = 0.29 [Pet. ether/EtOAc (9:1)]. ^1H NMR (500 MHz, $CDCl_3$) δ 8.42 (1H, dd, *J* = 6.2, 2.4 Hz, H4), 8.41

(1H, d, *J* = 2.5 Hz, H6), 7.69 - 7.64 (3H, m, H3, H2', H6'), 7.50 (1H, ddt, *J* = 7.9, 7.0, 1.3 Hz, H4'), 7.35 (2H, apparent t, *J* = 8.0 Hz, H3', H5'), 7.10 - 7.02 (2H, m, H4'', H6''), 6.97 (1H apparent t, *J* = 7.5 Hz, H5''), 2.14 (3H, d, *J* = 2.2 Hz, CH_3). ^{13}C NMR (126 MHz, $CDCl_3$) δ 195.24 (C), 157.34 (d, *J* = 246.3 Hz, CF), 147.08 (C), 142.13 (C), 140.46 (C), 136.27 (C), 133.53 (CH), 132.49 (CH), 132.45 CH), 129.87 (CH), 128.58 (d, *J* = 2.5 Hz, CH), 128.44 (CH), 125.71 (d, *J* = 15.7 Hz, C), 125.40 (d, *J* = 17.7 Hz, C) , 125.03 (CH), 124.18 (CH), 124.07 (d, *J* = 4.3 Hz, CH), 14.56 (d, *J* = 4.3 Hz, CH_3). ^{19}F NMR (377 MHz, $CDCl_3$) δ −119.51 (apparent td, *J* = 6.9, 2.2 Hz). ν_{Max}(ATR)cm^{-1}: 1670 (C=O), 1597 ($C=C_{Ar}$), 1582 ($C=C_{Ar}$), 1524 (NO_2), 1348 (NO_2). MS (EI^+): 335 ($M^{+\cdot}$, 97%), 320 ($M^{+\cdot} - {}^{\cdot}CH_3$, 55), 258 ($M^{+\cdot} - Ph^{\cdot}$, 43), 183 ($C_{13}H_8F^+$, 42), 105 ($PhCO^+$, 85), 86 (100). HRMS ($EI^+$): 335.0954. $C_{20}H_{14}FNO_3$ requires $M^{+\cdot}$, 335.0958. MP: 140 - 145 ˚C.

N-hexyl imine of 2-(2''-Fluoro-4''-*tert*butylphenyl)-5-nitrophenyl(phenyl)benzophenone **22a**

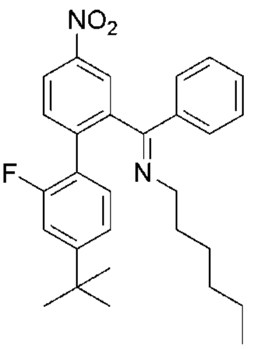

Benzophenone **21a** (1.01 g, 2.66 mmol, 1.00 eq.) and dry hexylamine (2.45 mL, 18.6 mmol, 6.97 eq.) were combined stirring under argon at 0 ˚C in dry PhMe (20.0 mL) then titanium tetrachloride (0.44 mL, 4.0 mmol, 1.5 eq.) was added over 5 min. The reaction was allowed to warm to RT and allowed to stir for 17 h. The mixture was quenched into H_2O, with the residue washed out with Et_2O. The layers were separated and the aqueous was then extracted (Et_2O, x 2) then the combined organics washed (brine) and dried ($MgSO_4$), then filtered and the solvent removed under reduced pressure. The imine was purified by chromatography [SiO_2, Pet. Ether/ Et_2O (20:1)] and isolated as a solid **22a** (665 mg, 54%). R_f = 0.26 Pet. Ether/Et_2O (20:1). ^{1}H NMR (500 MHz, $CDCl_3$) δ 8.34 (1H, dd, *J* = 8.5, 2.4 Hz, H4), 8.09 (1H, d, *J* = 2.4 Hz, H6), 7.67

(1H, dd, J = 8.5, 1.8 Hz, H3), 7.52 - 7.44 (2H, m, H2', H6'), 7.34 (1H, tt, J = 7.3, 1.3 Hz, H4'), 7.31 - 7.24 (2H, m, H3', H5'), 7.02 (1H, dd, J = 12.4, 1.7 Hz, H3''), 6.98 (1H, dd, J = 8.1, 1.8 Hz, H5''), 6.94 (1H, apparent t, J = 8.0 Hz, H6''), 3.26 - 3.18 (1H,m, $H1_A$'''), 3.08 (1H, ddd, J = 13.6, 7.8, 5.7 Hz, $H1_B$'''), 1.63 - 1.53 (1H, m, $H2_A$'''), 1.45 - 1.33 (1H, m, $H2_B$'''), 1.32 - 1.15 (15H, m, H3''', H4''', H5''', tBu), 0.85 (3H, t, J = 7.1 Hz, CH_3). ^{13}C NMR (126 MHz, $CDCl_3$) δ 164.11 (C), 159.13 (d, J = 247.1 Hz, CF), 155.28 (d, J = 6.8 Hz, C), 147.26 (C), 141.92 (C), 139.50 (C), 138.16 (C), 132.48 (d, J = 2.6 Hz, CH), 130.13 (CH), 129.94 (d, J = 2.9 Hz, CH), 128.29 (CH), 128.15 (CH), 124.05 (CH), 123.36 (CH), 122.72 (d, J = 14.6 Hz, C), 121.20 (d, J = 3.1 Hz, CH), 113.12 (d, J = 22.5 Hz, CH), 54.39 (CH_2), 34.97 (d, J = 1.4 Hz, C). 31.80 (CH_2), 31.14 (CH_3), 30.93 (CH_2), 27.29 (CH_2), 22.72 (CH_2), 14.19 (CH_3). ^{19}F NMR (377 MHz, $CDCl_3$) δ −116.01 (dd, J = 12.4, 7.8 Hz). ν_{Max}(ATR)cm^{-1}: 2958 (C-H), 2932 (C-H), 2859 (C-H), 1624 (C=N), 1578 (C_{Ar}=C_{Ar}), 1524 (NO_2), 1346 (NO_2). MS (EI^+): 460 ($M^{+\cdot}$, 7%), 441 ($C_{29}H_{33}N_2O_2^{+\cdot}$, 7), 403 ($C_{25}H_{24}FN_2O_2^{+}$, 22), 84 ($C_6H_{12}^{+\cdot}$, 100). HRMS: 460.2527. $C_{29}H_{33}FN_2O_2$ requires $M^{+\cdot}$, 460.2526. MP: decomp.

3-tert-Butyl-5-(hex-1'-yl)-6-phenyl-8-nitrophenanthridinium chloride **23a**

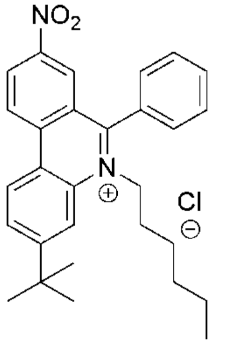

Benzophenone **21a** (500 mg, 1.33 mmol, 1.00 eq.) and dry hexylamine (1.05 mL, 9.31 mmol, 7.00 eq.) were combined stirring under argon at 0 ˚C in dry PhMe (5.0 mL) then a 1 M solution of titanium tetrachloride in dry PhMe (2.00 mL, 2.00 mmol, 1.50 eq.) was added over 5 min. The reaction was allowed to warm to RT and allowed to stir for 40 h. The mixture was quenched into 1 M $K_2CO_{3(aq)}$ then filtered through celite and extracted into DCM (x 3). The combined organics were acidified (0.55 M $HCl_{(aq)}$). The acidic aqueous extracts were further extracted (DCM, x 2), then the organic extracts

recombined and basified in 1 M $K_2CO_{3(aq)}$. The organic layers were dried ($MgSO_4$), then filtered and the solvent removed under reduced pressure. From the crude yield of 623 mg of extremely viscous brown oil, a portion (100 mg, 0.22 mmol, 1.00 eq.) was transferred into a microwave tube (10 mL) containing dry THF (5.0 mL). The tube was filled with argon and sealed then heated to 100 ˚C for 2 h. After cooling the material was transferred to a round bottom flask with DCM and the solvent removed under reduced pressure. The residue was dissolved in dry Et_2O then precipitated with 2 M ethereal HCl (1.00 mL, 2.00 mmol, 9.09 eq.). The precipitate was allowed to settle, then the bulk of the solvent syringed out and the remainder removed under reduced pressure, isolating the phenanthridinium chloride **23a** as a brown foam (87 mg, 85%). ^{1}H NMR (400 MHz, 0.75 mL CD_3CN + 0.05 mL CF_3CO_2D) δ 8.96 (1H, d, *J* = 9.2 Hz, H10), 8.86 (1H, d, *J* = 9.1 Hz, H1), 8.71 (1H, dd, *J* = 9.2, 2.3 Hz, H9), 8.16 - 8.05 (3H, m, H2, H4, H7), 7.72 - 7.67 (1H, m, H4''), 7.67 - 7.61 (2H, m, H3'', H5''), 7.51 - 7.46 (2H, m, H2'', H6''), 4.68 - 4.56 (2H, m, CH_2-1'), 1.85 – 1.76 (2H, m, CH_2-2''), 1.34 [s, 9H, $C(CH_3)_3$], 1.20 - 1.09 (2H, m, CH_2-3'), 1.00 (4H, m, CH_2-4', CH_2-5'), 0.63 (3H, t, *J* = 7.0 Hz, CH_3-6'). ^{13}C NMR (126 MHz, 0.75 mL CD_3CN + 0.05 mL CF_3CO_2D) δ 164.32 (C), 158.62 (C), 147.28 (C), 138.16 (C), 134.94 (C), 132.03 (CH), 129.95 (CH), 129.68 (CH), 129.62 (CH), 128.22 (CH), 127.91 (CH), 125.60 (CH), 125.38 (C), 125.19 (CH), 123.51 (C), 117.03 (CH), 116.30 (C), 55.26 (CH_2), 35.86 (C), 30.33 (CH_2), 29.89 (CH_3), 28.82 (CH_2), 25.66 (CH_2), 21.79 (CH_2), 12.84 (CH_3) . ν_{Max}(ATR)cm^{-1}: 2956 (C_{Ar}-H), 2927 (C_{Ar}-H), 2870 (C_{Ar}-H), 1620 (C=N), 1588 (C_{Ar}=C_{Ar}), 1539 (C_{Ar}=C_{Ar}), 1342 (NO_2). MS (ESI$^+$): 441 [M^+ (phenanthridinium cation), 100%]. HRMS: 441.2516. $C_{29}H_{33}N_2O_2$ requires M^+ (phenanthridinium cation), 441.2537.

<u>5-(Hex-1'-yl)-6-phenyl-8-nitrophenanthridinium chloride **23b**</u>

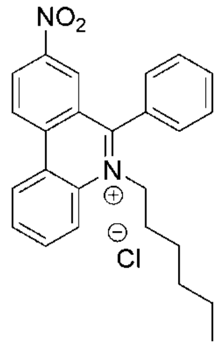

Benzophenone **21b** (253 mg, 0.79 mmol, 1.00 eq.) and dry hexylamine (0.73 mL, 5.5 mmol, 7.0 eq.) were combined stirring under argon at 0 ˚C in dry PhMe (3.0 mL) then 1 M titanium tetrachloride in dry PhMe (1.18 mL, 1.18 mmol, 1.49 eq.) was added over 5 min. The reaction was allowed to warm to RT and allowed to stir for 72 h. The mixture was quenched into brine then filtered and extracted into Et_2O (x 1) and DCM (x 2). The combined organics were dried ($MgSO_4$), then filtered and the solvent removed under reduced pressure. From the crude yield of 272 mg of extremely viscous brown oil, a portion (101 mg, 0.25 mmol, 1.00 eq.) was transferred into a microwave tube (10 mL) containing dry THF (5.0 mL). The tube was filled with argon and sealed then heated in a microwave to 100 ˚C for 2 h. The solution was filtered and the residue eluted with $CHCl_3$ and MeOH, then the solvent removed under reduced pressure, then the residue triturated with pet. ether, which was filtered. The residue and filter cake were recombined in $CHCl_3$ and the solvent removed under reduced pressure. The residue was then redissolved in dry Et_2O and precipitated with ethereal HCl (1.00 mL, 2.00 mmol, 7.69 eq.), then allowed to settle for 15 h. The solid was partitioned between Et_2O and 1 M $NaOH_{(aq)}$ then the aqueous re-extracted (Et_2O x 1, $CHCl_3$ x 2). The organic fractions were recombined, then ethereal HCl (1.00 mL, 2.00 mmol, 7.69 eq.) and PhMe were added and the solvent removed under reduced pressure, isolating the phenanthridinium chloride **23b** as brown solid (103 mg, 84%). ^1H NMR (500 MHz, CD_3CN + 0.05 mL CF_3CO_2D): 9.12 (1H, t, *J* = 9.0 Hz, H10), 9.07 (1H, d, *J* = 7.9 Hz, H1), 8.87 (1H, d, *J* = 8.6 Hz, H9), 8.48 (1H, d, *J* = 8.3 Hz, H4), 8.26 (1H, s, H7), 8.21 (1H, apparent t, br, *J* = 7.2 Hz, H3), 8.14 (1H, apparent t, br, *J* = 7.1 Hz, H2), 7.83 (1H, t, *J* = 7.2 Hz, H4''), 7.77 (2H, t, *J* = 6.8 Hz, H3'', H5''), 7.63 (2H, d, *J* = 6.9 Hz, H2'', H6''), 4.71 (2H, s, br, CH_2-1'), 2.00 - 1.89 (2H, m, CH_2-2'), 1.30 - 1.04 (6H, m, CH_2-3', CH_2-4', CH_2-5'), 0.75

(3H, t, J = 6.9 Hz, CH_2-6'). ^{13}C NMR (101 MHz, 0.75 mL MeOD + 0.05 mL CF_3CO_2D) δ 166.13 (C), 149.03 (C), 140.13 (C), 136.62 (C), 135.59 (CH), 133.53 (CH), 132.71 (CH), 131.58 (C), 131.45 (CH), 131.07 (CH), 130.03 (CH), 129.10 (CH), 127.37 (C), 127.35 (CH), 127.31 (C), 127.04 (CH), 122.28 (CH), 57.07 (CH_2), 31.94 (CH_2), 30.71 (CH_2), 27.20 (CH_2), 23.35 (CH_2), 14.17 (CH_3). ν_{Max}(ATR)cm^{-1}: 3067 (C_{Ar}-H), 2953 (C-H), 2926, (C-H), 2857 (C-H), 1620 (C=N), 1589 (C_{Ar}=C_{Ar}), 1537 (NO_2), 1341 (NO_2). MS (NSI^+): 385 [M^+ (phenanthridinium cation), 100%], 301 (M^+ – C_6H_{10}, 15). HRMS: 385.1916. $C_{25}H_{25}N_2O_2$ requires M^+ (phenanthridinium cation), 385.1911. MP: 190 - 192 °C.

3-Bromo-5-(hex-1'-yl)-6-phenyl-8-nitrophenanthridinium chloride **23c**

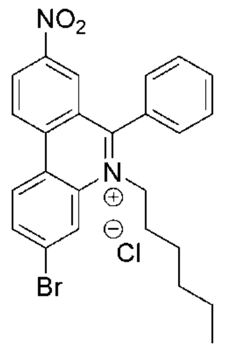

Benzophenone **21c** (530 mg, 1.32 mmol, 1.00 eq.) and dry hexylamine (1.75 mL, 13.2 mmol, 10.0 eq.) were combined stirring under argon at 0 °C in dry PhMe (5.0 mL) then 1 M titanium tetrachloride in dry PhMe (1.86 mL, 1.86 mmol, 1.41 eq.) was added. The reaction was allowed to warm to RT and allowed to stir for 15 h, at which point additional 1 M titanium tetrachloride in PhMe was added (0.50 mL, 0.50 mmol, 0.38 eq.). After stirring for an additional 20 h the mixture was quenched into 1 M $K_2CO_{3(aq)}$ then filtered and extracted into DCM (x 3). The combined organics were acidified (0.55 M $HCl_{(aq)}$). The acidic aqueous extracts were further extracted (DCM, x 2), then the organic extracts recombined and basified in 1 M $K_2CO_{3(aq)}$. The organic layers were dried ($MgSO_4$), then filtered and the solvent removed under reduced pressure. From the crude yield of 656 mg of extremely viscous brown oil, a portion (102 mg, 0.20 mmol, 1.00 eq.) was transferred into a microwave tube (10 mL) containing dry THF (5.0 mL). The tube was filled with argon and sealed then heated in a microwave to 100 °C for 2 h. After cooling the material

was transferred to a round bottom flask with $CHCl_3$ and the solvent removed under reduced pressure. The residue was dissolved in dry Et_2O then precipitated with 2 M ethereal HCl (1.00 mL, 2.00 mmol, 10.0 eq.). The precipitate was allowed to settle, then the bulk of the solvent syringed out and the remainder removed under reduced pressure, then the solid triturated with dry Et_2O, $CHCl_3$ and MeOH consecutively. The solvent was removed under reduced pressure, isolating the phenanthridinium chloride **23c** was isolated as an off-white solid (103 mg, 100%). ^{1}H NMR (400 MHz, MeOD) δ 9.25 (1H, d, *J* = 9.2 Hz, H10), 9.09 (1H, d, *J* = 8.9 Hz, H1), 8.93 (1H, dd, *J* = 9.2, 2.2 Hz, H9), 8.79 (1H, d, *J* = 1.4 Hz, H4), 8.33 – 8.27 (2H, m, H2, H7), 7.85 (1H, t, *J* = 7.5 Hz, H4''), 7.79 (2H, t, *J* = 7.3 Hz, H3'', H5''), 7.68 (2H, d, *J* = 7.4 Hz, H2'', H6''), 4.87 - 4.72 (2H, m, CH_2-1'), 2.00 - 1.86 (2H, m, CH_2-2'), 1.31 - 1.03 (6H, m, CH_2-3', CH_2-4', CH_2-5'), 0.81 - 0.72 (3H, t, *J* = 7.2 Hz, CH_2-6'). ^{13}C NMR (126 MHz, 0.75 mL MeOD + 0.05 mL CF_3CO_2D) δ 167.34 (C), 149.26 (C), 139.82 (C), 137.15 (C), 136.11 (CH), 133.68 (CH), 131.89 (CH), 131.25 (C), 131.06 (CH), 129.97 (C), 129.91 (CH), 129.29 (CH), 128.83 (CH), 127.30 (C), 127.02 (CH), 126.36 (C), 124.82 (CH), 57.10 (CH_2), 31.76 (CH_2), 30.55 (CH_2), 27.00 (CH_2), 23.15 (CH_2), 13.92 (CH_3). ν_{Max}(ATR)cm^{-1}: 2955 (C-H), 2930 (C-H), 2857 (C-H), 1618 (C=N), 1603 (C_{Ar}=C_{Ar}), 1589 (C_{Ar}=C_{Ar}), 1533 (NO_2), 1518 (C_{Ar}=C_{Ar}), 1341 (NO_2). MS (ESI$^+$): 465 [^{81}BrM$^+$ (phenanthridinium cation), 100%], 463 [^{79}BrM$^+$ (phenanthridinium cation), 98]. HRMS: 463.1011 and 465.0986. $C_{25}H_{24}{}^{79}BrN_2O_2$ requires M$^+$ (phenanthridinium cation), 463.1016 and $C_{25}H_{24}{}^{81}BrN_2O_2$ requires M$^+$ (phenanthridinium cation), 465.0996. MP: 160 - 165 ˚C.

5-(Hex-1'-yl)-6-phenylphenanthridinium chloride **23d**

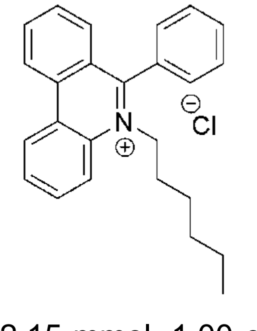

Benzophenone **21d** (594 mg, 2.15 mmol, 1.00 eq.) and dry hexylamine (2.00 mL, 15.1 mmol, 7.04 eq.) were combined stirring under argon at 0 ˚C in dry

PhMe (5.0 mL) then titanium tetrachloride (0.35 mL, 3.2 mmol, 1.5 eq.) was added over 5 min. The reaction was allowed to warm to RT and allowed to stir for 72 h. The mixture was quenched into brine, and extracted with diethyl ether (x 1), EtOAc (x 1) and DCM (x 5). The combined organics were dried ($MgSO_4$) then filtered and the solvent removed under reduced pressure. From the crude yield of 537 mg of brown oil a portion (100 mg, 0.28 mmol, 1.00 eq.) was transferred into a microwave tube (10 mL) containing dry MeCN (5.0 mL). The tube was filled with argon and sealed then heated in a microwave to 110 ˚C for 90 min. After cooling the material was transferred to a round bottom flask with DCM and the solvent removed under reduced pressure. The residue was dissolved in dry Et_2O then precipitated with 2 M ethereal HCl (1.00 mL, 2.00 mmol, 7.14 eq.) The precipitate was allowed to settle, then the bulk of the solvent syringed out and the remainder removed under reduced pressure, isolating the product **23d** as a white solid (102 mg, 68%). ^{1}H NMR (500 MHz, 0.75 mL CD_3CN + 0.05 mL CF_3CO_2D) δ 9.01 (1H, d, *J* = 7.6 Hz, H1), 8.94 (1H, d, *J* = 8.1 Hz, H10), 8.40 (1H, d, *J* = 8.0 Hz, H4), 8.21 (1H, apparent t, *J* = 7.3 Hz, H9), 8.12 - 8.00 (2H, m, H2, H3), 7.80 - 7.67 (4H, m, H8, H2'', H3'', H5''), 7.61 - 7.50 (3H, m, H7, H2'', H6''), 4.74 - 4.55 (2H, m, CH_2-1'), 2.00 - 1.85 (2H, m, CH_2-2'), 1.30 - 1.18 (2H, m, CH_2-3'), 1.16 - 1.00 (m, CH_2-4', CH_2-5'), 0.74 (3H, t, *J* = 7.0 Hz, CH_2-6'). ^{13}C NMR (101 MHz, 0.75 mL CD_3CN + 0.05 mL CF_3CO_2D) δ 164.01 (C), 137.53 (CH), 135.04 (C), 133.91 (C), 132.91 (CH), 132.38 (CH), 131.46 (CH), 130.79 (C), 130.48 (CH), 130.13 (CH), 129.43 (CH), 128.11 (CH), 126.37 (C), 125.74 (C), 124.68 (CH), 122.83 (CH), 120.39 (CH), 54.86 (CH_2), 30.31 (CH_2), 28.97 (CH_2), 25.64 (CH_2), 21.83 (CH_2), 12.85 (CH_3). ν_{Max}(ATR)cm^{-1}: 3051 (C_{Ar}-H), 2953 (C-H), 2926 (C-H), 2861 (C-H), 1609 (C=N), 1584 (C_{Ar}=C_{Ar}), 1574 (C_{Ar}=C_{Ar}). MS (NSI^+): 340 [M^+ (phenanthridinium cation), 100%], 256 (M^+ – C_6H_{12}, 52). HRMS: 340.2058. $C_{25}H_{26}N$ requires M^+ (phenanthridinium cation), 340.2060. MP: 194 - 195 ˚C.

<u>2- Methoxy-5-(hex-1'-yl)-6-phenyl-8-nitrophenanthridinium chloride **23e**</u>

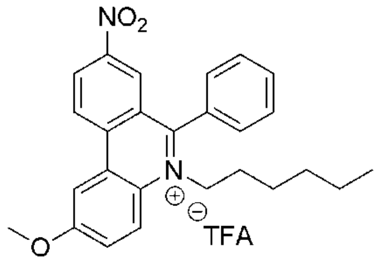

Benzophenone **21e** (102 mg, 0.29 mmol, 1.00 eq.) and dry hexylamine (0.27 mL, 2.0 mmol, 7.1 eq.) were combined stirring under argon at 0 ˚C in dry PhMe (1.0 mL) then titanium tetrachloride (0.42 mL, 0.4 mmol, 1.5 eq.) was added. The reaction was allowed to warm to RT and allowed to stir for 20 h. The mixture was quenched into H_2O and extracted into diethyl ether (x 2). The combined organics were dried ($MgSO_4$), then filtered and the solvent removed under reduced pressure. The crude yield of 114 mg was transferred in dry $CHCl_3$ into a kugelrohr bulb and the chloroform distilled out, then the oil heated at 100 ˚C for 2 h 45 min, then at 125 ˚C for 3 h. TFA was added then evaporated off under reduced pressure. The mixture was partitioned from a water/TFA mixture with DCM (x 3), and $CHCl_3$ (x 3). The organics were combined and the solvent removed under reduced pressure. Following column chromatography [SiO_2, loaded and washed in DCM then eluted with DCM/MeOH/NEt_3 (97:2:1)]. The solvent was removed under reduced pressure and replaced with $CHCl_3$ then the compound dried ($MgSO_4$). The mixture was filtered, then TFA was added then and the solvent removed under reduced pressure. The phenanthridinium trifluoroacetate **23e** was isolated as brown oil (70 mg, 47%). ^{1}H NMR (500 MHz, $CDCl_3$) δ 9.08 (1H, d, *J* = 9.1 Hz, H10), 8.77 (1H, dd, *J* = 9.1, 2.1 Hz, H9), 8.39 (1H, d, *J* = 9.6 Hz, H4), 8.34 (1H, d, *J* = 2.2 Hz, H7), 8.27 (1H, d, *J* = 2.5 Hz, H1), 7.86 – 7.82 (1H, m, H4”), 7.78 (2H, t, *J* = 7.8 Hz, H3”, H5”), 7.74 (1H, dd, *J* = 9.6, 2.6 Hz, H3), 7.65 (2H, d, *J* = 7.7 Hz, H2”, H6”), 4.94 – 4.79 (2H, m, CH_2-1’), 4.10 (3H, s, OCH_3), 1.93 (2H, quin, *J* = 7.9 Hz, CH_2-2’), 1.34 – 1.08 (6H, m, CH_2-3’, CH_2-4’, CH_2-5’), 0.81 (3H, t, *J* = 7.2 Hz, CH_2-6’). ^{13}C NMR (126 MHz, $CDCl_3$) δ 161.75 (C), 160.61 (C), 147.64 (C), 137.85 (C), 132.43 (CH), 130.11 (C), 129.73 (C), 129.57 (CH), 128.84 (CH), 128.05 (C), 127.74 (CH), 126.39 (CH), 125.70 (C), 124.60 (CH), 122.64 (CH), 106.64 (CH), 56.82 (CH_3), 56.09 (CH_2), 30.76 (CH_2), 30.05 (CH_2), 26.19 (CH_2), 22.26 (CH_2), 13.74 (CH_3).

ν_{Max}(ATR)cm^{-1}: 2958 (C-H), 2920 (C-H), 2850 (C-H), 1777 (TFA), 1731 (TFA), 1614 (C=N), 1541 (C_{Ar}=C_{Ar}), 1592 (C_{Ar}=C_{Ar}), 1348 (NO_2). MS (NSI^+): 415 [M^+ (phenanthridinium cation), 100%]. HRMS: 415.2005, $C_{26}H_{27}N_2O_3$ requires M^+ (phenanthridinium cation), 415.2016.

3,8-dinitro-5-(hex-1'-yl)-6-phenylphenanthridinium chloride **23f**

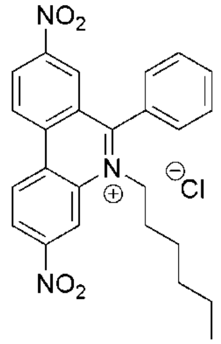

Benzophenone **21f** (390 mg, 1.06 mmol, 1.00 eq.) and dry hexylamine (1.00 mL, 7.57 mmol, 7.14 eq.) were combined stirring under argon at 0 ˚C in dry PhMe (5.5 mL) then a 1 M solution of titanium tetrachloride in dry PhMe (1.60 mL, 1.60 mmol, 1.51 eq.) was added over 5 min. The reaction was allowed to warm to RT and allowed to stir for 48 h. The mixture was quenched into H_2O, and the resulting precipitate washed with Et_2O, $CHCl_3$, DCM, MeCN and MeOH and the organics combined and washed with brine and extracted DCM (x 1), Et_2O (x 1), and $CHCl_3$ (x 4) . The combined organics were dried ($MgSO_4$) then filtered and the solvent removed under reduced pressure. From the crude yield of 504 mg of brown solid a portion (100 mg, 0.22 mmol, 1.00 eq.) was transferred to a round bottom flask with DCM and the solvent removed under reduced pressure. The residue was dissolved in dry Et_2O then precipitated with 2 M ethereal HCl (1.00 mL, 2.00 mmol, 9.09 eq.). The precipitate was allowed to settle, then the bulk of the solvent syringed out and the remainder removed under reduced pressure, isolating the phenanthridinium chloride **23f** as a powder (95 mg, 97%). ^{1}H NMR (500 MHz, MeOD) δ 9.58 (1H, d, *J* = 9.2 Hz, H1), 9.52 (1H, d, *J* = 9.1 Hz, H10), 9.44 (1H, d, *J* = 1.9 Hz, H4), 9.15 (1H, dd, *J* = 9.1, 2.3 Hz, H9), 9.01 (1H, dd, *J* = 9.1, 1.9 Hz, H2), 8.49 (1H, d, *J* = 2.2 Hz, H7), 8.01 (1H, t, *J* = 7.5 Hz, H4''), 7.96 (2H, t, *J* = 7.5 Hz, H3'', H5''), 7.86 (2H, d, *J* = 7.8 Hz, H2'', H6''), 5.10 - 4.99 (2H, m, CH_2-1'), 2.20 - 2.08 (2H, m, CH_2-2'), 1.49 - 1.40 (2H, m, CH_2-3'), 1.36

- 1.23 (4H, m, CH_2-4', CH_2-5'), 0.92 (3H, t, *J* = 7.1 Hz, CH_2-6'). ^{13}C NMR (126 MHz, MeOD) δ 170.05 (C), 152.37 (C), 150.81 (C), 139.67 (C), 137.15 (C), 134.72 (CH), 133.09 (CH), 132.01 (C), 131.97 (CH), 131.93 (C), 130.41 (CH), 130.32 (CH), 130.17 (CH), 129.44 (C), 128.82 (CH), 126.82 (CH), 118.76 (CH), 58.12 (CH_2), 32.68 (CH_2), 31.39 (CH_2), 27.95 (CH_2), 24.16 (CH_2), 15.01 (CH_3). ν_{Max}(ATR)cm^{-1}: 2955 (C-H), 2928 (C-H), 2859 (C-H), 1618 (C=N), 1591 (C_{Ar}=C_{Ar}), 1537 (C_{Ar}=C_{Ar}), 1518 (NO_2), 1343 (NO_2). MS (NSI$^+$): 430 [M$^+$ (phenanthridinium cation), 100%]. HRMS: 430.1754. $C_{25}H_{24}N_3O_4$ requires M$^+$ (phenanthridinium cation), 430.1761. MP: 189 ˚C.

<u>2-Methoxy-3,8-dinitro-5-(hex-1'-yl)-6-phenylphenanthridinium chloride **23g**</u>

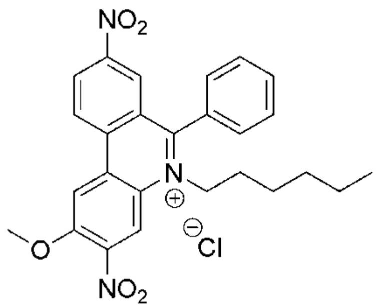

Benzophenone **21g** (501 mg, 1.26 mmol, 1.00 eq.) and dry hexylamine (1.17 mL, 8.86 mmol, 7.03 eq.) were combined stirring under argon at 0 ˚C in dry PhMe (5.0 mL) then a 1 M solution of titanium tetrachloride in dry PhMe (1.90 mL, 1.90 mmol, 1.51 eq.) was added over 5 min. The reaction was allowed to warm to RT and allowed to stir for 40 h. The mixture was quenched into H_2O, filtered through celite with DCM and partitioned between DCM and 0.5 M $HCl_{(aq)}$, then the aqueous layer was re-extracted (DCM x 2). The combined organics were dried ($MgSO_4$) then filtered and the solvent removed under reduced pressure. From the crude yield of 572 mg of brown solid a portion (100 mg, 0.21 mmol, 1.00 eq.) was was transferred into a microwave tube (10 mL) containing dry THF (5.0 mL). The tube was filled with argon and sealed then heated in a microwave to 100 ˚C for 2 h. The solvent was removed under reduced pressure, then the residue dissolved in dry Et_2O and precipitated with 2 M ethereal HCl (1.00 mL, 2.00 mmol, 9.52 eq.). The precipitate was allowed to settle, then the bulk of the solvent syringed out and the process repeated. After the remaining solvent was removed under reduced pressure the phenanthridinium chloride **23g** was isolated as a powder (80 mg, 74%). ^{1}H

NMR (400 MHz, 0.75 mL CD_3CN + 0.05 mL CF_3CO_2D) δ 9.31 (1H, d, *J* = 9.2 Hz, H10), 9.02 (1H, dd, *J* = 9.2, 2.2 Hz, H9), 8.90 (1H, s, H4), 8.64 (1H, s, H1), 8.36 (1H, d, *J* = 2.2 Hz, H7), 7.94 (1H, t, *J* = 7.5 Hz, H4''), 7.87 (2H, t, *J* = 7.3 Hz, H3'', H5''), 7.71 (2H, d, *J* = 7.1 Hz, H2'', H6''), 4.79 - 4.71 (2H, m, CH_2-1'), 4.36 (3H, s, OCH_3), 2.06 - 1.91 (2H, m, CH_2-2'), 1.39 - 1.10 (6H, m, CH_2-3', CH_2-4', CH_2-5'), 0.84 (t, *J* = 7.1 Hz, CH_2-6'). ^{13}C NMR (126 MHz, 0.75 mL DMSO + 0.05 mL CF_3CO_2D) δ 163.79 (C), 151.64 (C), 147.82 (C), 143.26 (C), 136.31 (C), 131.79 (CH), 129.70 (C), 129.63 (CH), 129.28 (CH), 129.06 (C), 128.44 (C), 128.36 (CH), 127.13 (CH), 127.06 (CH), 126.81 (C), 117.75 (CH), 109.21 (CH), 58.14 (CH_3), 55.44 (CH_2), 29.90 (CH_2), 28.53 (CH_2), 25.19 (CH_2), 21.47 (CH_2), 13.16 (CH_3). ν_{Max}(ATR)cm^{-1}: 3018 (C_{Ar}-H), 2954 (C-H), 2922 (C-H), 2917 (C-H), 2853 (C-H), 1621 (C=N), 1595 (C_{Ar}=C_{Ar}), 1537 (NO_2), 1521 (C_{Ar}=C_{Ar}), 1501 (C_{Ar}=C_{Ar}), 1350 (NO_2). MS (ESI^+): 460 [M^+ (phenanthridinium cation), 100%]. HRMS: 460.1846. $C_{26}H_{26}N_3O_5$ requires M^+ (phenanthridinium cation), 460.1867. MP: 195 - 198 ˚C.

3,8-Dinitro-5-(hex-1'-yl)-6-methylene-5-hydrophenanthridine **23h**

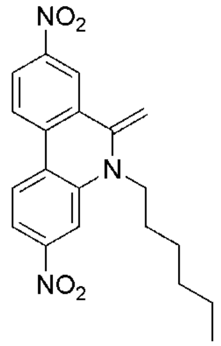

Benzophenone **21h** (426 mg, 1.40 mmol, 1.00 eq.) and dry hexylamine (1.45 mL, 11.0 mmol, 7.58 eq.) were combined stirring under argon at 0 ˚C in dry PhMe (5.0 mL) then a 1 M solution of titanium tetrachloride in dry PhMe (2.70 mL, 2.70 mmol, 1.93 eq.) was added over 5 min. The reaction was allowed to warm to RT and allowed to stir for 19 h. The mixture was quenched into H_2O, then filtered through celite with DCM and partitioned between DCM and 1 M $HCl_{(aq)}$, then the aqueous layer was re-extracted (DCM x 2). The combined organics were washed with 1 M $HCl_{(aq)}$ then $NaHCO_{3(aq)}$ and dried ($MgSO_4$) then filtered and the solvent removed under reduced pressure. The enamine was isolated as a purple solid **23h** (523 mg, quant.). ^1H NMR (400 MHz,

$CDCl_3$) δ 8.62 (1H, d, *J* = 2.3 Hz, H7), 8.22 (1H, dd, *J* = 8.9, 2.3 Hz, H9), 8.03 (1H, d, *J* = 8.9 Hz, H10), 7.96 (1H, d, *J* = 8.7 Hz, H1), 7.74 (1H, dd, *J* = 8.7, 2.2 Hz, H2), 7.69 (1H, d, *J* = 2.1 Hz, H4), 4.96 (1H, d, *J* = 3.1 Hz, $NC=CH_{trans}$), 4.39 (1H, d, *J* = 3.2 Hz, $NC=CH_{cis}$), 3.83 - 3.69 (2H, m, CH_2-1'), 1.83 (2H, quint., J = 7.8 Hz, CH_2-2'), 1.56 - 1.47 (2H, m, CH_2-3'), 1.47 - 1.36 (2H, m, CH_2-4', CH_2-5'), 0.95 (3H, t, *J* = 7.1 Hz, CH_2-6'). ^{13}C NMR (101 MHz, $CDCl_3$) δ 149.91 (C), 148.41 (C), 142.18 (C), 141.83 (C), 133.65 (C), 132.01 (C), 125.05 (CH), 124.37 (CH), 123.72 (C), 123.54 (CH), 120.68 (CH), 113.94 (CH), 108.01 (CH), 86.80 (CH_2), 48.23 (CH_2), 31.50 (CH_2), 26.69 (CH_2), 24.43 (CH_2), 22.75 (CH_2), 14.11 (CH_3). ν_{Max}(ATR)cm^{-1}: 2957 (C-H), 2926 (C-H), 2856 (C-H), 1603 ($C_{Ar}=C_{Ar}$), 1578 ($C_{Ar}=C_{Ar}$), 1520 (NO_2), 1339 (NO_2). MS ($APCI^+$): 368 $[(M+H)^+$, 100]. HRMS: 368.1603. $C_{20}H_{22}N_3O_4$ requires $(M+H)^+$, 368.1605. MP: 169 - 171 ˚C.

2,8-dinitro-5-(hex-1'-yl)-6-phenylphenanthridinium chloride **23i**

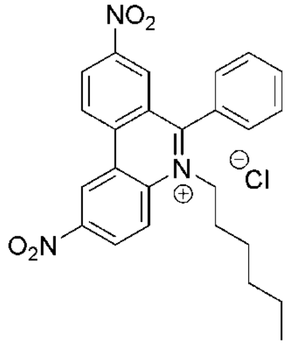

Benzophenone **21i** (300 mg, 0.78 mmol, 1.00 eq.) and dry hexylamine (0.60 mL, 4.6 mmol, 5.9 eq.) were combined stirring under argon at 0 ˚C in dry PhMe (3.0 mL) then 1 M titanium tetrachloride in dry PhMe (0.98 mL, 0.98 mmol, 1.3 eq.) was added over 5 min. The reaction was allowed to warm to RT and allowed to stir for 20 h. A sample (0.1 mL) was quenched into H_2O, partitioned between DCM and water and the organic separated and the solvent removed under reduced pressure. Proton NMR confirmed the reaction was complete. The remaining mixture was quenched into H_2O, then filtered through celite with DCM and partitioned between DCM and 1 M $HCl_{(aq),}$ then the aqueous layer was re-extracted (DCM x 2). The combined organics were washed with 1 M $HCl_{(aq)}$ then $NaHCO_{3(aq)}$ and dried ($MgSO_4$) then filtered and the solvent removed under reduced pressure. From the crude yield of 336 mg

of brown solid a portion (100 mg, 0.21 mmol, 1.00 eq.) was transferred into a microwave tube (10 mL) containing dry THF (5.0 mL). The tube was filled with argon and sealed then heated in a microwave to 100 ˚C for 30 min then cooled and transferred to a round bottomed flask in dry $CHCl_3$, then the solvent removed under reduced pressure. The residue was dissolved in dry Et_2O then precipitated with 2 M ethereal HCl (1.00 mL, 2.00 mmol, 9.35 eq.). The solvent was removed under reduced pressure. The solid was dissolved in DCM, dried ($MgSO_4$) and filtered, then the solvent removed under reduced pressure. The salt **23i** was isolated as a solid (91 mg, 86%). ^{1}H NMR [500 MHz, CD_3CN/CF_3CO_2D (10:1)] δ 9.82 (1H, d, *J* = 2.3 Hz, H1), 9.27 (1H, d, *J* = 9.1 Hz, H10), 8.95 (1H, dd, *J* = 9.1, 2.2 Hz, H9), 8.84 (1H, dd, *J* = 9.5, 2.2 Hz, H3), 8.66 (1H, d, *J* = 9.5 Hz, H4), 8.29 (1H, d, *J* = 2.2 Hz, H7), 7.86 (1H, t, *J* = 7.5 Hz, H4''), 7.79 (2H, t, *J* = 7.5 Hz, H3'', H5''), 7.63 (2H, d, *J* = 7.3 Hz, H2'', H6''), 4.79 - 4.66 (2H, m, CH_2-1'), 1.99 - 1.88 (2H, m, CH_2-2'), 1.30 - 1.03 (6H, m, CH_2-3', CH_2-4', CH_2-5'), 0.75 (3H, t, *J* = 7.2 Hz, CH_2-6'). ^{13}C NMR [126 MHz, CD_3CN/CF_3CO_2D (10:1)] δ 167.94 (C), 148.39 (C), 148.15 (C), 138.14 (C), 137.68 (C), 132.56 (CH), 131.23 (CH), 129.83 (CH), 129.38 (C), 128.56 (CH), 128.09 (CH), 127.42 (CH), 126.64 (C), 126.35 (C), 126.25 (CH), 123.34 (CH), 121.76 (CH), 56.45 (CH_2), 30.31 (CH_2), 28.88 (CH_2), 25.61 (CH_2), 21.89 (CH_2), 12.99 (CH_3). ν_{Max}(ATR)cm^{-1}: 3099 (C_{Ar}-H), 2957 (C-H), 2927 (C-H), 2857 (C-H), 1601 (C=N), 1578 (C_{Ar}=C_{Ar}), 1526 (NO_2), 1494 (NO_2), 1342 (NO_2), 1326 (NO_2). MS (NSI$^+$): 430 [M^+ (phenanthridinium cation), 100%]. HRMS: 430.1752. $C_{25}H_{24}N_3O_4$ requires M^+ (phenanthridinium cation), 430.1761. MP: 260 - 263 ˚C.

4-aza-5-(hex-1'-yl)-6-phenyl-8-nitrophenanthridinium chloride **23j**

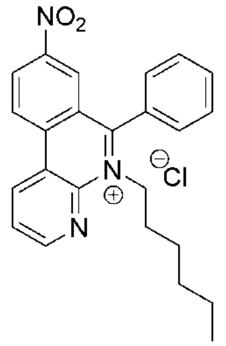

See paper for experimental, characterization data: ^{1}H NMR (400 MHz, MeOD) δ 9.71 (1H, dd, J = 8.5, 1.7 Hz, H1), 9.41 (1H, d, J = 9.1 Hz, H10), 9.38 (1H, dd, J = 4.4, 1.7 Hz, H3), 9.08 (1H, dd, J = 9.1, 2.3 Hz, H9), 8.49 (1H, d, J = 2.0 Hz, H7), 8.28 (1H, dd, J = 8.4, 4.4 Hz, H2), 7.97 (1H, t, J = 7.4 Hz, H4''), 7.91 (2H, t, J = 7.3 Hz, H3'', H5''), 7.85 - 7.79 (2H, d, J = 7.4 Hz, H2'', H6''), 5.12 - 5.02 (2H, m, CH_2-1'), 2.08 - 1.97 (2H, m, CH_2-2'), 1.40 - 1.16 (6H, m, CH_2-3', CH_2-4', CH_2-5'), 0.87 (3H, t, J = 7.1 Hz, CH_2-6'). ^{13}C NMR (126 MHz, $CDCl_3$) δ 167.29 (C), 153.69 (CH), 147.66 (C), 144.54 (C), 138.75 (C), 136.83 (CH), 132.35 (CH), 130.23 (CH), 129.73 (CH), 129.62 (C), 128.77 (CH), 128.02 (CH), 127.77 (CH), 126.80 (CH), 126.32 (C), 121.51 (C), 54.80 (CH_2), 30.65 (CH_2), 29.78 (CH_2), 26.47 (CH_2), 22.28 (CH_2), 13.86 (CH_3). ν_{Max}(ATR)cm^{-1}: 3064 (C_{Ar}-H), 2956 (C-H), 2931 (C-H), 2926 (C-H), 1623 (C=N), 1604 (C_{Ar}=C_{Ar}), 1592 (C_{Ar}=C_{Ar}), 1538 (NO_2), 1518 (C_{Ar}=C_{Ar}), 1343 (NO_2). MS (ESI^+): 418 [M^+ (phenanthridinium cation) + MeOH, 62%], 386 [M^+ (phenanthridinium cation), 100], 302 [(M^+ (phenanthridinium cation) – C_6H_{12}, 69). HRMS: 386.1857. $C_{24}H_{24}N_3O_2$ requires M^+ (phenanthridinium cation), 386.1863. MP: 188 - 189 ˚C.

<u>4- Methyl-5-(hex-1’-yl)-6-phenyl-8-nitrophenanthridinium chloride **23k**</u>

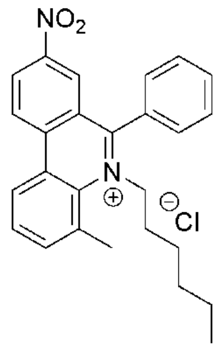

Benzophenone **21k** (526 mg, 1.57 mmol, 1.00 eq.) and dry hexylamine (1.45 mL, 11.0 mmol, 7.01 eq.) were combined stirring under argon at 0 ˚C in dry PhMe (5.0 mL) then titanium tetrachloride (0.26 mL, 2.4 mmol, 1.5 eq.) was added. The reaction was allowed to warm to RT and allowed to stir for 72 h. The mixture was quenched into brine then filtered and extracted into diethyl ether (x 1) and DCM (x 2). The combined organics were dried ($MgSO_4$), then filtered and the solvent removed under reduced pressure. From the crude yield of 695 mg of extremely viscous brown oil, a portion (100 mg, 0.24 mmol, 1.00 eq.) was transferred into a microwave tube (10 mL) containing dry MeCN (5.0 mL). The tube was filled with argon and sealed then heated in a microwave to 110 ˚C for 10 h. After cooling the material was transferred to a round bottom flask with DCM and the solvent removed under reduced pressure. The residue was dissolved in dry Et_2O then precipitated with 2 M ethereal HCl (1.00 mL, 2.00 mmol, 8.33 eq.). The precipitate was allowed to settle, then the bulk of the solvent syringed out and the remainder removed under reduced pressure, then the residue loaded onto a Strata WCX column in MeOH, then the column washed with additional MeOH before elution with NaOAc/MeOH (15 mg/mL). The solvent was removed under reduced pressure and replaced with $CHCl_3$ then the compound dried ($MgSO_4$) and the solution filtered then the solvent removed under reduced pressure. The phenanthridinium chloride **23k** was isolated as brown oil (77 mg, 79%). ^1H NMR (500 MHz, MeOD) δ 9.31 (1H, d, J = 9.2 Hz, H10), 9.10 (1H, dd, J = 7.6, 1.9 Hz, H1), 9.00 (1H, dd, J = 9.2, 2.3 Hz, H9), 8.48 (1H, d, J = 2.3 Hz, H7), 8.14 - 8.06 (2H, m, H2, H3), 8.00 - 7.94 (1H, m, H4’’), 7.93 - 7.85 (4H, m, H2’’, H3’’, H5’’, H6’’), 5.05 (2H, t, J = 6.9 Hz, CH_2-1’), 3.07 (3H, s, $ArCH_3$), 1.54 (2H, quintet, J = 7.7 Hz, CH_2-2’), 1.08 - 1.00 (2H, m, CH_2-3’), 0.99 - 0.84 (4H, m,

CH_2-4’, CH_2-5’), 0.71 (3H, t, J = 7.2 Hz, CH_2-6’). ^{13}C NMR (126 MHz, MeOD) δ 169.05 (C), 148.88 (C), 140.64 (C), 139.63 (CH), 137.15 (C), 134.20 (CH), 133.25 (C), 132.45 (C), 132.11 (CH), 131.72 (CH), 131.43 (CH), 131.07 (CH), 129.26 (CH), 128.59 (C), 126.88 (CH), 126.82 (C), 125.11 (CH), 61.15 (CH_2), 31.75 (CH_2), 31.02 (CH_2), 26.66 (CH_2), 24.53 (CH_3), 23.02 (CH_2), 14.00 (CH_3). ν_{Max}(ATR)cm^{-1} (TFA salt): 2961 (C-H), 2930 (C-H), 2862 (C-H), 1778 (TFA), 1732 (TFA), 1622 (C=N), 1586 (C_{Ar}=C_{Ar}), 1539 (C_{Ar}=C_{Ar}), 1348 (NO_2). MS (ESI^+): 399 [M^+ (phenanthridinium cation), 100%]. HRMS: 399.2050. $C_{26}H_{27}N_2O_2$ requires M^+ (phenanthridinium cation), 399.2067.

3-*tert*-Butyl-5-(cyclohexylmethylamino)-6-phenyl-8-nitrophenanthridinium chloride **24a**

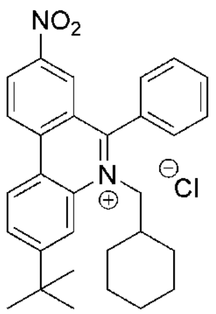

Benzophenone **21a** (360 mg, 0.95 mmol, 1.00 eq.) and cyclohexylmethylamine (0.90 mL, 6.9 mmol, 7.3 eq.) were combined stirring under argon at 0 ˚C in dry PhMe (5.0 mL) then a 1 M solution of titanium tetrachloride in dry PhMe (1.50 mL, 1.50 mmol, 1.50 eq.) was added over 5 min. The reaction was allowed to warm to RT and allowed to stir for 20 h. The mixture was quenched into H_2O, then filtered through celite with DCM and partitioned between DCM and 0.5 M $HCl_{(aq)}$, then the aqueous layer was re-extracted (DCM x 2). The combined organics were washed with 1 M $HCl_{(aq)}$ then $NaHCO_{3(aq)}$ and dried ($MgSO_4$) then filtered and the solvent removed under reduced pressure. From the crude yield of 445 mg of brown solid four portions (total of 80 mg, 0.16 mmol, 1.00 eq.) were transferred into microwave tubes (10 mL) containing dry MeCN (1.0 mL). The tubes were filled with argon and sealed then heated in a microwave to 110 ˚C for 90 min then cooled and the solvent removed under reduced pressure. Following column chromatography of the combined residues [SiO_2, loaded in and

washed with DCM and eluted with DCM/NEt_3 (200:1)] the combined organics were concentrated under reduced pressure then washed (1 M $HCl_{(aq)}$ x 2), and dried ($MgSO_4$). After filtration 2 M ethereal HCl (1.00 mL, 2.00 mmol, 12.5 eq.) was added to the filtrate and the solvent removed under reduced pressure, to give phenanthridinium salt **24a** as a brown solid (63 mg, 75%). R_f = 0.21 in DCM/NEt_3 (100:1). ^{1}H NMR (400 MHz, MeOD, 50˚C) δ 9.32 (1H, d, *J* = 9.2 Hz, H10), 9.20 (1H, d, *J* = 8.8 Hz, H1), 8.99 (1H, dd, *J* = 9.2, 2.2 Hz, H9), 8.49 - 8.43 (2H, m, H4, H7), 8.37 (1H, dd, *J* = 8.8, 1.4 Hz, H2), 7.94 (1H, t, *J* = 7.4 Hz, H4''), 7.88 (2H, t, *J* = 7.3 Hz, H3'', H5''), 7.79 (2H, d, *J* = 7.0 Hz, H2'', H6''), 5.00 (2H, s, br, CH_2-N), 2.08 (1H, m, CH_2-1'), 1.73 - 1.49 (2H, m, CH_2-2'$_A$), 1.59 [9H, s, C(CH_3)], 1.40 - 0.82 (8H, m, CH_2-2'$_B$, CH_2-4', CH_2-5'). ^{13}C NMR (126 MHz, DMSO, 70 ˚C) δ 164.57 (C), 157.00 (C), 146.73 (C), 137.58 (C), 135.08 (C), 131.65 (CH), 129.94 (C), 129.49 (CH), 129.31 (CH), 128.89 (CH), 128.82 (CH), 127.30 (CH), 125.55 (C), 125.31 (CH), 125.03 (CH), 123.00 (C), 117.19 (CH), 59.46 (CH_2), 38.29 (CH), 35.65 (C), 30.32 (CH_3), 29.88 (CH_2), 24.82 (CH_2). ν_{Max}(ATR)cm^{-1}: 2926 (C-H), 2853 (C-H), 1621 (C=N), 1587 (C_{Ar}=C_{Ar}), 1540 (C_{Ar}=C_{Ar}), 1343 (NO_2). MS (ESI$^+$): 453 [M$^+$ (phenanthridinium cation), 100]. HRMS: 453.2523. $C_{30}H_{33}N_2O_2$ requires M$^+$ (phenanthridinium cation), 453.2537.

5-(6'-Azidohex-1'-yl)-6-phenylphenanthridinium chloride **25d**

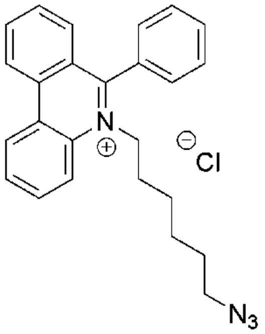

Benzophenone **21d** (414 mg, 1.49 mmol, 1.00 eq.) and 6-azidohex-1-ylamine (1.00 g, 7.86 mmol, 5.28 eq.) were combined stirring under argon at 0 ˚C in dry PhMe (7.0 mL) then 1 M titanium tetrachloride in dry PhMe (1.70 mL, 1.70 mmol, 1.14 eq.) was added over 5 min. After 15 h a sample (0.1 mL) was quenched into H_2O, partitioned between DCM and water and the organic separated and the solvent removed under reduced pressure. This process

was repeated at 21 h, at which point the starting material was shown to be consumed by proton NMR. The mixture was quenched into water then filtered through celite with DCM, then partitioned between DCM and 1 M $HCl_{(aq)}$. The aqueous was extracted with DCM (x 2) then the collected organics washed with 1 M $HCl_{(aq)}$, followed by $NaHCO_{3(aq)}$. The organic layers were dried ($MgSO_4$), then filtered and the solvent removed under reduced pressure. From the crude yield of 571 mg of brown oil a portion (100 mg, 0.25 mmol, 1.00 eq.) was transferred into a microwave tube (10 mL) containing dry MeCN (5.0 mL). The tube was filled with argon and sealed then heated in a microwave to 110 ˚C for 90 min. After cooling the residue was dissolved in MeOH and loaded onto a Strata SCX ion exchange column and washed (MeOH) then eluted (15 mg/mL NaOAc in MeOH). The solution was partitioned between $CHCl_3$ and 1 M $HCl_{(aq)}$, then the organic layer washed (1 M $HCl_{(aq)}$ x 2). The organic layer was dried ($MgSO_4$), filtered and ethereal HCl (1.00 mL, 2.00 mmol, 7.75 eq.) was added, then the solvent removed under reduced pressure. The phenanthridinium chloride **25d** was isolated as a brown oil (79 mg, 78%). ^{1}H NMR (400 MHz, 0.75 mL $CDCl_3$ + 0.05 mL CF_3CO_2D) δ 8.99 (1H, dd, *J* = 8.1, 1.7 Hz, H1), 8.93 (1H, d, *J* = 8.5 Hz, H10), 8.40 (1H, d, *J* = 8.3 Hz, H4), 8.30 (1H, ddd, *J* = 8.5, 7.2, 1.5 Hz, H9), 8.20 - 8.11 (2H, m, H2, H3), 7.81 - 7.67 (4H, m, H8, H3'', H4'', H5''), 7.68 (1H, d, *J* = 7.7 Hz, H7), 7.60 - 7.53 (2H, m, H2'', H6''), 4.89 - 4.79 (2H, m, CH_2-1'), 3.26 (2H, t, *J* = 6.7 Hz, CH_2-6'), 2.01 (2H, s, br, CH_2-2'), 1.58 - 1.49 (2H, m, CH_2-3'), 1.42 - 1.23 (4H, m, CH_2-4', CH_2-5'). ^{13}C NMR (101 MHz, 0.75 mL $CDCl_3$ + 0.05 mL CF_3CO_2D) δ 163.82 (C), 138.43 (CH), 135.27 (C), 133.35 (C), 133.21 (CH), 133.09 (CH), 132.37 (CH), 131.21 (CH), 130.71 (CH), 130.05 (CH), 127.62 (CH), 126.38 (C), 125.10 (C), 124.83 (CH), 122.75 (CH), 119.35 (CH), 54.47 (CH_2), 51.02 (CH_2), 29.75 (CH_2), 28.06 (CH_2), 25.84 (CH_2), 25.62 (CH_2). ν_{Max}(ATR)cm^{-1} (as TFA salt): 2928 (C-H), 2856 (C-H), 1779 (TFA), 1738 (TFA), 1622 (C=N), 1588 (C_{Ar}=C_{Ar}), 1538(C_{Ar}=C_{Ar}). MS (NSI^+): 381 [M^+ (phenanthridinium cation), 38%], 256 (M^+ – C_6H_{12}, 100). HRMS: 381.2073. $C_{25}H_{25}N_4$ requires M^+ (phenanthridinium cation), 381.2074.

3-Bromo-5-(hex-1'-yl)-6-phenyl-8-aminophenanthridinium chloride **26c**

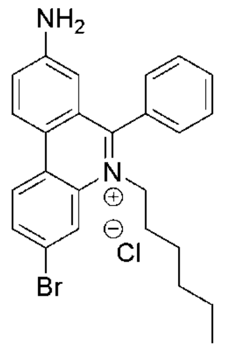

Nitrophenanthridinium **23c** (250 mg, 0.50 mmol, 1.00 eq.) was dissolved in a 1:1 mixture of AcOH and EtOH (15.0 mL) then iron powder (346 mg, 6.20 mmol, 10.7 eq.) added and the mixture heated stirring to 40 ˚C for 90 min. The mixture was then poured into 1 M $HCl_{(aq)}$, and extracted with DCM (x 2). The combined organics were then washed with 1 M $HCl_{(aq)}$ and $NaHCO_{3(aq)}$ (x 2) sequentially, before drying ($MgSO_4$), filtering and removing the solvent under reduced pressure. The amine **26c** was isolated as a red powder (200 mg, 85%). ^{1}H NMR (500 MHz, $CDCl_3$) δ 8.57 (1H, d, *J* = 8.9 Hz, H1), 8.48 (1H, d, *J* = 8.6 Hz, H10), 8.24 (1H, s, H4), 7.98 (1H, d, *J* = 8.7 Hz, H2), 7.83 - 7.68 (4H, m, H9, H3", H4", H5"), 7.49 (2H, d, *J* = 6.8 Hz, H2", H6"), 6.52 (1H, s, H7), 4.95 (2H, s, br, NH_2), 4.63 (2H, t, *J* = 8.4 Hz, CH_2-1'), 1.95 - 1.83 (2H, m, CH_2-2'), 1.36 - 1.11 (6H, m, CH_2-3', CH_2-4', CH_2-5'), 0.84 (3H, t, *J* = 7.2 Hz, CH_2-6'). ^{13}C NMR (126 MHz, $CDCl_3$) δ 162.20 (C), 151.19 (C), 133.51 (CH), 132.28 (C), 131.91 (CH), 131.01 (C), 130.11 (CH), 129.92 (CH), 128.22 (CH), 127.55 (C), 126.08 (C), 125.78 (C), 125.45 (CH), 123.85 (C), 123.55 (CH), 122.14 (CH), 111.20 (CH), 54.75 (CH_2), 30.74 (CH_2), 29.67 (CH_2), 26.26 (CH_2), 22.34 (CH_2), 13.91 (CH_3). ν_{Max}(ATR)cm^{-1}: 3277 (N-H), 3133 (N-H), 2952 (C-H), 2929 (C-H), 2859 (C-H), 1609 (C=N), 1579 (C_{Ar}=C_{Ar}). MS (ESI$^+$): 435 [^{81}BrM$^+$ (phenanthridinium cation), 100%], 433 [^{79}BrM$^+$ (phenanthridinium cation], 100). HRMS: 435.1242 and 433.1255. $C_{25}H_{26}{}^{81}BrN_2$ requires M$^+$ (phenanthridinium cation), 435.1253 and $C_{25}H_{26}{}^{79}BrN_2$ requires M$^+$ (phenanthridinium cation), 433.1274. MP: 153 - 157 ˚C.

References

1. a) D. W. Kuykendall, C. A. Anderson, S. C. Zimmerman, *Org. Lett.* **2009**, *11*, 61-64; b) J. W. Lee, S. I. Jun, K. Kim, *Tetrahedron Lett.* **2001**, *42*, 2709-2711.

2. I. Sapountzis, W. Lin, C. C. Kofink, C. Despotopoulou, P. Knochel, *Angew. Chem. Int. Ed.* **2005**, *44*, 1654-1657.

3. I. Sapountzis, H. Dube, R. Lewis, N. Gommermann, P. Knochel, *J. Org. Chem.* **2005**, *70*, 2445-2454.

4. H. A. Wegner, H. Reisch, K. Rauch, A. Demeter, K. A. Zachariasse, A. de Meijere, L. T. Scott, *J. Org. Chem.* **2006**, *71*, 9080-7.

5. B. D. Bath, S. J. Pasaribu, L. R. Williams, *Org. Magn. Reson.* **1977**, *9*, 210-212.

Expanding the palette of phenanthridinium cations

Andrew G. Cairns, Hans Martin Senn, Michael P. Murphy, and Richard C. Hartley

Supplementary Information 2

Supplementary Computational Details and Results

1 Diffuse functions in the def2-TZVP+ basis set

The def2-TZVP+ basis set was derived from the standard def2-TZVP[1] by augmenting it with one uncontracted diffuse function per valence angular momentum on all atoms (corresponding to a "++" set in Pople-style notation). The exponents of the diffuse Gaussians were determined according to a geometric progression from the exponents of the two outermost uncontracted functions of each valence angular momentum (even-tempered set).

2 Input preparation and visualization

Initial structures were prepared with Avogadro.[2-3] Output was visualized with Avogadro[2-3] and Jmol.[4]

3 Experimental thermodynamic conditions

The reference reaction was performed in MeCN solvent in a capped vial under microwave heating. The temperature was controlled at T = 383 K, however, the pressure was neither specified nor measured. The reaction temperature lies above the boiling point of MeCN, which is $T_b°$ = 355 K at the standard pressure of 1 bar.[5] The reaction pressure was estimated as follows.

At any temperature, the total pressure in the vial is the sum of the partial pressures of solvent and air. At 298 K,

$$p_{tot}(298\ \mathrm{K}) = p_{MeCN}(298\ \mathrm{K}) + p_{air}(298\ \mathrm{K}) = 1\ \mathrm{bar}.$$

The saturation vapour pressure of MeCN at 298 K is $p_{MeCN}(298\ \mathrm{K})$ = 0.117 bar (using the Antoine equation[5]). Therefore, the partial pressure of air is $p_{air}(298\ \mathrm{K})$ = 0.883 bar.

Because the vial is closed, and assuming that the liquid phase is incompressible and that the decrease in liquid volume due to evaporation is negligible, the vapour-phase volume stays constant when the reaction mixture is heated. At 383 K, the air partial pressure therefore increases to

$$p_{air}(383\ \mathrm{K}) = p_{air}(298\ \mathrm{K}) \times 383\ \mathrm{K}/298\ \mathrm{K} = 1.135\ \mathrm{bar}.$$

The partial pressure of MeCN at 383 K can be calculated from the Clausius–Clapeyron equation,

$$\ln\frac{p_2}{p_1} = \frac{\Delta_{vap}H}{R}\left(\frac{1}{T_1} - \frac{1}{T_2}\right),$$

where the points (p_1, T_1) and (p_2, T_2) lie on the liquid–vapour coexistence curve. The equation as written assumes that $\Delta_{vap}H$ is temperature-independent. For $p_1 = p°$ = 1 bar, $T_1 = T_b°$ = 355 K, and

$\Delta_{vap}H$(355 K) = 29.75 kJ mol^{-1},[5] the saturation vapour pressure at T_2 = 383 K is $p_2 = p_{MeCN}$(383 K) = 2.089 bar.

The reaction pressure at 383 K is therefore

$$p_{tot}(383\ \text{K}) = 1.135\ \text{bar} + 2.089\ \text{bar} = 3.224\ \text{bar} \approx 320\ \text{kPa} = 3.158\ \text{atm}.$$

4 Calibration of the solvent model

4.1 General remarks

We calculate the solvation free energy as

$$\Delta_s G(p, T) = G_{sln}(p, T) - G_g(p, T),$$

where the Gibb free energies of the two states ("g" – gas phase, "sln" – solution) are obtained from a full optimization and frequency calculation for the respective state. The usual ideal gas/rigid rotor/harmonic oscillator approximation is applied to calculate the Gibbs energies.

Within this approximation, G is pressure-dependent only through the translational entropy contribution, which contains a term $\ln V = \ln(kT/p)$. (The enthalpic volume work $pV = kT$ has no net pressure-dependence.) As the ideal-gas approximation discards all molecular detail (such as changes in structure between different phases), the pressure-dependent contributions at given temperature (and constant number of particles) cancel out when calculating entropy or free-energy *differences*. $\Delta_s G$ therefore depends on temperature, but not on pressure.

To calculate the free energy in solution, G_{sln}, we use a polarizable continuum model (PCM). The contribution of the solvent model is included in the electronic (SCF) energy. The only parameter characterizing the solvent is the relative permittivity, ε_r, which depends on temperature, but not pressure (see below). In the presence of a PCM, the SCF energy becomes implicitly temperature-dependent through $\varepsilon_r(T)$.

In the case of *monoatomic* solutes, which have no internal degrees of freedom, the only finite-temperature contributions to G are translational. As these are independent of the state (g or sln), they cancel out when calculating energy differences such as $\Delta_s G$. Hence, for single atoms or ions, $\Delta_s G(T)$ is temperature-dependent *only* through $\varepsilon_r(T)$.

The calibration of the atomic solvation radii, which are the main parameters in a PCM, against experimental solvation energies is therefore valid at any pressure for given T.

The solvation contribution to reaction energies is dominated by the formation/disappearance of ions, which are far more strongly solvated than neutral species. Especially small and/or highly charged ions contribute the most. In the system at hand, solvation is thus dominated by the free halide anions. It is therefore justified to recalibrate only the atomic solvation radii of the halides fluoride and chloride.

4.2 Relative permittivity of MeCN under reaction conditions

The relative permittivity (dielectric constant) of a liquid is almost independent of pressure, but depends strongly on temperature.[6] (This is predominantly a density effect; liquids are nearly incompressible, so the pressure-dependence is very small.) The temperature-dependence of ε_r is well described by a linear fit (R^2 = 0.921, see Figure S1). Using the straight-line equation, we can extrapolate

$$\varepsilon_r(383\ \text{K}) = 22.5.$$

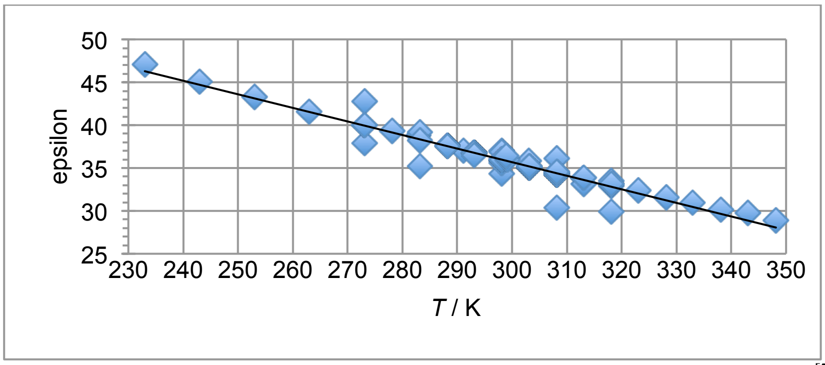

Figure S1. Temperature-dependence of ε_r(MeCN). 82 data points taken from ref.[7] The straight-line equation is $\varepsilon_r = -0.1584\ T/\mathrm{K} + 83.20$.

4.3 Experimental solvation free energies of halide anions in MeCN

4.3.1 Caveats and notes on standard states

The reliable experimental determination of solvation parameters, particularly of single ions, is fraught with a number of experimental, methodical, and conceptual difficulties, compounded by a lack of definitional and terminological clarity.[8] For non-aqueous solutions, data are even scarcer and difficult to validate.

Our approach to experimental solvation free energies in MeCN is to use the best available hydration free energies and to combine them with free energies of transfer from water to MeCN,

$$\Delta_s G(\mathrm{MeCN}) = \Delta_{\mathrm{hyd}} G + \Delta_t G(\mathrm{H_2O} \rightarrow \mathrm{MeCN}).$$

Whenever possible, we have taken the hydration data from the excellent monograph by Hünenberger and Reif,[8] who provide rigorously consistent and validated data.

Following Ben-Naim, we consider the solvation process as the transfer of a solute from a fixed point in the gas phase to a fixed point in solution under standard conditions. The associated solvation free energy is designated $\Delta_s G^*$. In the language of Hünenberger/Reif, Ben-Naim's $\Delta_s G^*$ is the "semi-standard point-to-point solvation free energy" using $bMMe_T$ standard states: Reference pressure $p° = 10^5$ Pa (b); standard concentration for both gas and solution $c^{\triangledown} = 1$ mol dm^{-3} (MM); warm electrons (e_T); the standard temperature is $T^{-} = 298.15$ K.

Hünenberger/Reif use the symbol $\Delta_s G^{\ominus}$ to designate the point-to-point solvation free energy, and they prefer the $bbme_T$ standard (reference pressure $p° = 10^5$ Pa [b]; standard pressure of the gas $p°$ = 10^5 Pa [b]; standard concentration of the solution $b° = 1$ mol kg^{-1} [m]; warm electrons [e_T]; standard temperature $T^{-} = 298.15$ K). However, $\Delta_s G^{\ominus}$ is the same for either standard state, hence $\Delta_s G^* = \Delta_s G^{\ominus}$. (Coincidentally, for water at T^{-}, $\Delta_s G^{\ominus}$ is also the intrinsic solvation free energy in the $bMMe_T$ standard, $\Delta_s G^{\triangledown}$.)

In keeping with the work of Cramer and Truhlar (among others), we calibrate our solvation model against experimental $\Delta_s G^*$ ($\Delta_s G^{\ominus}$ in Hünenberger/Reif) values, corrected for the reaction temperature.

4.3.2 Hydration free energies of halide anions at non-standard temperature

In order to calculate $\Delta_{\mathrm{hyd}} G^*$ of the halides under reaction conditions from the standard values in Table S1, we assume that (i) $\Delta_{\mathrm{hyd}} G^*$ is independent of pressure (see above); (ii) $\Delta_{\mathrm{hyd}} C_p^*$ is independent of temperature. Therefore, we can correct $\Delta_{\mathrm{hyd}} H^*$ and $\Delta_{\mathrm{hyd}} S^*$ for a non-standard temperature using the standard $\Delta_{\mathrm{hyd}} C_p^* = \Delta_{\mathrm{hyd}} C_p^*(T^{-})$.

Table S1. Semi-standard point-to-point hydration parameters for fluoride and chloride (from ref.[8]).

X^-	$\Delta_{hyd}G^{\ominus}$ / (kJ mol^{-1})	$\Delta_{hyd}H^{\ominus}$ / (kJ mol^{-1})	$\Delta_{hyd}S^{\ominus}$ / (J mol^{-1} K^{-1})	$\Delta_{hyd}C_p^{\ominus}$ / (J mol^{-1} K^{-1})
F^-	–441.13	–471.86	–103.09	–71.03
Cl^-	–316.55	–328.80	–41.09	–81.90

Using

$$\mathrm{d}H = C_p\,\mathrm{d}T \quad \text{and} \quad \mathrm{d}S = (C_p/T)\,\mathrm{d}T$$

and integrating between the standard temperature T^{-} and the target temperature T, we obtain the Gibbs energy at temperature T,

$$\Delta_s G^*(T) = \Delta_s H^*(T^-) + (T - T^-)\Delta_s C_p^* - T\left(\Delta_s S^*(T^-) + \Delta_s C_p^* \ln\frac{T}{T^-}\right)$$

Table 2 lists the required halide hydration parameters at $T = 383$ K.

Table S2. Point-to-point hydration parameters for fluoride and chloride at $T = 383$ K.

X^-	$\Delta_{hyd}G^*$(383 K) / (kJ mol^{-1})	$\Delta_{hyd}H^*$(383 K) / (kJ mol^{-1})	$\Delta_{hyd}S^*$(383 K) / (J mol^{-1} K^{-1})
F^-	–431.59	–477.89	–120.88
Cl^-	–312.16	–335.75	–61.60

4.3.3 Free energies of transfer from water to MeCN

We have relied primarily on the 2007 compilation by Marcus[9] of transfer free energies of anions from water into organic solvents. The values therein are reported for $T = 298.15$ and a standard concentration of 1 mol dm^{-3}. However, single-ion transfer free energies are subject to considerable uncertainty and deserve further scrutiny.

The situation is especially precarious for F^-; essentially, there are "higher" and "lower" values proposed in the literature. Marcus[9] offers lower values of 45 and 49.3 kJ mol^{-1}, without expressing a preference. The value of 45 kJ mol^{-1} was derived[10] from the solubility of NaF in MeCN/H_2O mixtures. However, the same author in a review that appeared shortly thereafter[11] proposed the higher value of 70 kJ mol^{-1} as "best value", derived from solubility data for KF, RbF, and CsF, arguing that the results for the lighter alkali fluoride salts (LiF, NaF) are unreliable. That higher value was again presented in subsequent work.[12] In good agreement with this, a value of 71 kJ mol^{-1} was reported independently[13] from potentiometric measurements on NaF solutions using a fluoride-selective electrode.

The other lower value of 49.3 kJ mol^{-1} listed in ref.[9] also stems from fluoride-selective electrode measurements.[14] Unfortunately, Marcus[9] does not comment on why the lower values should be preferable; indeed, the higher values are not mentioned at all. An indication might be taken from the finding[15] that lower values are consistent with a number of experimental solvent acidity and polarity parameters, whereas higher values are not.

Recently, a value of 65 kJ mol^{-1} was reported,[16] based on voltammetric measurements.

The situation is clearly not satisfactory. Accepting the choice made by Marcus,[9] we decided to use $\Delta_t G°(F^-, H_2O \rightarrow \text{MeCN}) = 49$ kJ mol^{-1}. A better justified decision would require a detailed analysis of the primary measurements and careful consideration of the origins and reliability of the various auxiliary quantities used to derive $\Delta_t G°$, which is clearly beyond the scope of the present exercise.

Enthalpies, entropies, and heat capacities of transfer, required to calculate $\Delta_t G$(383 K), are even more elusive. For F^-, the only values available[11] are $\Delta_t H°(F^-, H_2O \rightarrow \text{MeCN}) = 30$ kJ mol^{-1} and

$\Delta_t S°(F^-, H_2O \rightarrow MeCN) = -134$ J mol^{-1} K^{-1} (which are consistent with $\Delta_t G° = 70$ kJ mol^{-1}, at variance with our choice of 49 kJ mol^{-1}).

However, despite the considerable uncertainty about the exact values, it is safe to assume that $\Delta_t S°(X^-, H_2O \rightarrow MeCN)$ will be (strongly) negative. We can further assume that $\Delta_t G°$ is temperature-dependent only through $-T\Delta_t S°$ (*i.e.*, $\Delta_t H°$ and $\Delta_t S°$ are not themselves dependent on temperature). Given the transfer free energy at a standard temperature $T°$, $\Delta_t G°(T°)$, we can therefore calculate the free energy of transfer at a temperature T from

$$\Delta_t G(T) = \Delta_t G°(T°) - (T - T°)\Delta_t S°.$$

Transfer parameters for Cl^- are somewhat easier to obtain. Marcus[9] tentatively recommends $\Delta_t G°(Cl^-, H_2O \rightarrow MeCN) = 42.1$ kJ mol^{-1}, which is the average of several sources and will be used here. The more recent[16] value 45 kJ mol^{-1} is in good agreement with this. $\Delta_t S°(Cl^-, H_2O \rightarrow MeCN)$ is taken from the compilation in ref.[17].

Table S3 summarizes the transfer parameters for (X^-, $H_2O \rightarrow$ MeCN) that will be used here.

Table S3. Free energies and entropies of transfer from water to MeCN for fluoride and chloride.

X^-	$\Delta_t G°$(298 K) / (kJ mol^{-1})	$\Delta_t G°$(383 K) / (kJ mol^{-1})	$\Delta_t S°$ / (J mol^{-1} K^{-1})
F^-	49	60	–134
Cl^-	42.1	49.5	–87

4.3.4 "Best estimates" for experimental halide solvation energies

We can finally combine the hydration (Tables S1, S2) and transfer (Table S3) free energies to obtain the solvation free energies in MeCN at temperature T,

$$\Delta_s G_T^*(X^-, \text{MeCN}) = \Delta_{hyd} G_T^*(X^-) + \Delta_t G_T^*(X^-, H_2O \rightarrow \text{MeCN}).$$

Our "best estimates" for these quantities are given in Table S4.

Table S4. Free energies of solvation in MeCN for fluoride and chloride.

X^-	$\Delta_s G^*(X^-$, MeCN) / (kJ mol^{-1})	
	298 K	383 K
F^-	–392	–372
Cl^-	–274	–263

5 NBO analyses

NBO analyses were performed with the NBO 6.0 program,[18-20] using the NBO archive file produced by Gaussian (with `$nbo archive $end`). Because of the extensive conjugation in the systems investigated, the selection of the best localized (Lewis) structure is somewhat ambiguous, especially for the TS. Indeed, NBO chose different Lewis structures for differently substituted TSs (see Figure S2), preventing direct comparison of NBO results. We therefore manually prescribed the Lewis structure to be **B** in all cases (`$choose`). The quantitative differences between **A** and **B** are negligible; for instance, for **TSa**, **A** has 4.82e in non-Lewis orbitals, while **B** has 4.84e. The program would therefore choose **A** in this instance by default.

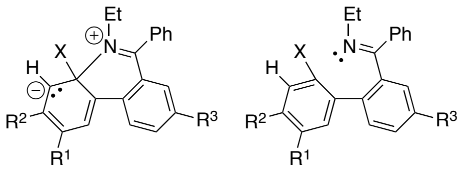

A **B**

Figure S2. Alternative localized Lewis structures for NBO analyses of the TS; structure **B** was used in all cases.

The partially formed bond between the imine lone pair and the X-bearing aryl carbon in the TS appears in the NBO picture as a 3-center-4-electron "hyperbond", that is, a resonance between limiting structures [C–C(X) :N] and [C: C(X)–N], corresponding to the Lewis structures **A** and **B**. The C–C(X) and C(X)–N bonds contribute almost equally (54% and 46% in **TSa**), and the hyperbond is occupied with 3.58e.

NBOs were visualized in Jmol,[4] using a script by Marcel Patek.[21]

6 Potential energies *vs.* free energies

Table S5 shows the potential-energy differences for the reaction, in addition to the Gibbs free energies, which are discussed in the main paper. Finite-temperature effects on barriers are small (<5 kJ mol^{-1}), similarly for the ion pairs **III**. Entropy is obviously a major factor in the complete separation of ions ($\mathbf{III}^+ + X^-$), which energetically is always unfavourable.

Table S5. Relative Gibbs free energies (383 K, 320 kPa) and potential (electronic) energies.[a]

	ΔG / (kJ mol^{-1})				ΔE / (kJ mol^{-1})			
	**TS**	**III**	$\mathbf{III}^+ + X^-$	**IV**	**TS**	**III**	$\mathbf{III}^+ + X^-$	**IV**
**a**	103	–65	–81	–79	99	–69	–53	–94
**b**	98	–57	–74	–84	95	–59	–39	–96
**c**	93	–60	–72	–93	89	–60	–41	–100
**d**	72	–52	–71	–89	66	–58	–43	–105
**e**	115	–70	–90	–78	112	–78	–63	–92
**f**	90	–51	–59	–88	87	–50	–27	–101
**g**	112	–61	–77	–76	106	–68	–50	–95
**h**	120	–126	–133	[b]	120	–131	–108	[b]
$\mathbf{a}_{\mathbf{vac}}$[c]	112	80[d]	440	–80	107	73[d]	471	107

[a] Calculated at M06-2X/def2-TZVP+ level in polarizable continuum MeCN (ε_r = 22.5). [b] The 6-chlorophenanthridine **IVh** is not a stable minimum, but dissociates into the ion pair **IIIh** during structure optimization. [c] Calculated in vacuum. [d] Energy of $\mathbf{IIa}_{\mathbf{vac}}$. In vacuum, the ion pair $\mathbf{IIIa}_{\mathbf{vac}}$ is not stable, but collapses to $\mathbf{IIa}_{\mathbf{vac}}$.

7 References

[1] F. Weigend, R. Ahlrichs, *Phys. Chem. Chem. Phys.* **2005**, *7*, 3297–3305.
[2] *Avogadro*, an open-source molecular builder and visualization tool, V. 1.1.0, 2012, http://avogadro.openmolecules.net/.
[3] M. D. Hanwell, D. E. Curtis, D. C. Lonie, T. Vandermeersch, E. Zurek, G. R. Hutchison, *J. Cheminf.* **2012**, *4*, 17.
[4] *JMol*, an open-source Java viewer for chemical structures in 3D, V. 13.0, 2013, http://www.jmol.org/.
[5] *NIST Chemistry WebBook* (Eds.: P. J. Linstrom, W. G. Mallard), NIST Standard Reference Database, Number 69, National Institute of Standards and Technology, Gaithersburg, MD, 2013, http://webbook.nist.gov/.
[6] J. F. Skinner, E. L. Cussler, R. M. Fuoss, *J. Phys. Chem.* **1968**, *72*, 1057–1064.

[7] C. Wohlfarth, in *Static Dielectric Constants of Pure Liquids and Binary Liquid Mixtures* (Ed.: M. D. Lechner), Landolt-Börnstein, IV/17, Springer, Berlin, **2008**, pp. 117–121.
[8] P. Hünenberger, M. Reif, *Single-Ion Solvation: Experimental and Theoretical Approaches to Elusive Thermodynamic Quantities*, Royal Society of Chemistry, London, **2011**.
[9] Y. Marcus, *Chem. Rev.* **2007**, *107*, 3880–3897.
[10] G. T. Hefter, P. J. McLay, *J. Solution Chem.* **1988**, *17*, 535–546.
[11] G. T. Hefter, *Rev. Inorg. Chem.* **1989**, *10*, 185–223.
[12] G. T. Hefter, *Pure Appl. Chem.* **1991**, *63*, 1749–1758.
[13] J. F. Coetzee, M. W. Martin, *Anal. Chem.* **1980**, *52*, 2412–2416.
[14] J. Bessière, F. Bazine, *J. Fluorine Chem.* **1989**, *44*, 45–58.
[15] D. C. Luehrs, K. A. Godbole, *J. Solution Chem.* **1990**, *19*, 187–192.
[16] A. Doménech, I. O. Koshevoy, N. Montoya, A. J. Karttunen, T. A. Pakkanen, *J. Chem. Eng. Data* **2011**, *56*, 4577–4586.
[17] G. Hefter, Y. Marcus, W. E. Waghorne, *Chem. Rev.* **2002**, *102*, 2773–2835.
[18] E. D. Glendening, J. K. Badenhoop, A. E. Reed, J. E. Carpenter, J. A. Bohmann, C. M. Morales, C. R. Landis, F. Weinhold, *NBO*, V. 6.0, Madison, WI, Theoretical Chemistry Institute, University of Wisconsin, 2013, http://nbo6.chem.wisc.edu/.
[19] E. D. Glendening, C. R. Landis, F. Weinhold, *J. Comput. Chem.* **2013**, *34*, 1429–1437.
[20] E. D. Glendening, C. R. Landis, F. Weinhold, *J. Comput. Chem.* **2013**, *34*, 2134–2134.
[21] M. Patek, *Visualizing NBOs in Jmol*, http://chemgplus.blogspot.co.uk/2012/09/visualizing-nbos-in-jmol.html, 2012, accessed 07/2013.

8 Cartesian coordinates of optimized structures

```
41
Ia
C  2.810409  0.273156 -1.047608
C  3.392255 -0.816002 -0.413503
C  2.608626 -1.667549  0.354102
C  1.258486 -1.409046  0.457794
C  0.632007 -0.339478 -0.167853
C  1.447749  0.506443 -0.919038
C -0.819961 -0.064092 -0.009209
C -1.824131 -1.015600 -0.254436
C -1.520770 -2.378051 -0.798562
N -0.664906 -2.460928 -1.731118
C -1.191180  1.207922  0.427531
C -2.522296  1.544507  0.624396
C -3.511420  0.599042  0.395783
C -3.156718 -0.671401 -0.031650
H  3.415858  0.941403 -1.646255
H  4.453498 -1.005660 -0.509748
H  3.028124 -2.519310  0.873866
H  0.993257  1.350481 -1.424052
H -0.416373  1.937117  0.632246
H -2.782119  2.538714  0.965788
H -4.554060  0.847082  0.549367
H -3.928454 -1.409947 -0.214930
C -2.282026 -3.512949 -0.184660
C -2.341123 -3.638946  1.203138
H -1.835273 -2.913150  1.828510
C -3.041666 -4.685903  1.784938
H -3.068783 -4.780451  2.863371
C -3.713975 -5.603401  0.986792
H -4.266961 -6.416348  1.440836
C -3.684707 -5.467386 -0.395237
H -4.220416 -6.169466 -1.022043
C -2.969979 -4.429751 -0.977924
H -2.956845 -4.323285 -2.055765
C -0.219061 -3.723959 -2.289749
C -0.608748 -3.820061 -3.757830
H -0.581653 -4.593979 -1.737299
H  0.872757 -3.718994 -2.218392
H -1.693715 -3.817324 -3.876040
H -0.216667 -4.740405 -4.192676
H -0.204142 -2.974155 -4.315688
F  0.516471 -2.239584  1.221367

43
Ib
C  4.299783 -1.049417 -0.093220
C  3.925104 -1.927733  0.914208
C  2.715093 -1.751019  1.572548
C  1.905481 -0.701256  1.195668
C  2.235518  0.193195  0.185841
```

```
C  3.464387  0.001172 -0.445337
C  1.362563  1.334686 -0.186790
C -0.009322  1.204207 -0.468042
C -0.705780 -0.123083 -0.523856
N -0.093543 -1.074329 -1.093504
C  1.951144  2.600116 -0.245427
C  1.217930  3.727374 -0.566601
C -0.131995  3.569978 -0.819494
C -0.754363  2.337872 -0.768260
H  5.242197 -1.182521 -0.608276
H  4.571873 -2.748990  1.194840
H  2.393897 -2.408843  2.369777
H  3.753076  0.680594 -1.238265
H  3.004282  2.702370 -0.017433
H  1.678558  4.703745 -0.607505
H -1.812342  2.258070 -0.977335
C -2.076346 -0.165895  0.076880
C -2.290516  0.325692  1.364075
H -1.459122  0.726731  1.931281
C -3.561223  0.301283  1.921916
H -3.714228  0.673307  2.927298
C -4.634930 -0.188764  1.188697
H -5.628357 -0.198580  1.619721
C -4.433433 -0.654702 -0.104152
H -5.269175 -1.022356 -0.686316
C -3.159919 -0.645897 -0.656683
H -3.007745 -0.999202 -1.669159
C -0.612713 -2.428262 -1.155099
C -0.852946 -2.838418 -2.600535
H -1.516316 -2.573581 -0.558520
H  0.168986 -3.068431 -0.735405
H -1.630809 -2.224946 -3.058251
H -1.165859 -3.882267 -2.646986
H  0.060019 -2.724510 -3.186934
F  0.740584 -0.533424  1.855035
N -0.935203  4.752425 -1.155561
O -2.109897  4.591152 -1.407906
O -0.380913  5.830717 -1.164122

43
Ic
C  4.296691 -1.062437 -0.179411
C  3.924379 -1.916015  0.841449
C  2.743129 -1.762899  1.548729
C  1.928735 -0.716474  1.186369
C  2.233607  0.172169  0.158264
C  3.444115 -0.021189 -0.506420
C  1.361253  1.322740 -0.186082
C -0.011930  1.205659 -0.456963
C -0.707340 -0.119015 -0.515893
N -0.090152 -1.089332 -1.050851
C  1.954919  2.584464 -0.226342
C  1.214223  3.718331 -0.523617
C -0.144543  3.604441 -0.778371
C -0.747388  2.356666 -0.734961
H  5.227027 -1.209558 -0.707701
H  2.466365 -2.426885  2.355134
H  3.710404  0.653067 -1.310298
H  3.011338  2.675438 -0.003604
H  1.698094  4.686594 -0.546159
H -1.807859  2.266540 -0.937866
C -2.090187 -0.156586  0.057163
C -2.321154  0.300966  1.353421
H -1.494173  0.673347  1.946363
C -3.603034  0.281400  1.885382
H -3.770761  0.628047  2.897492
C -4.669417 -0.169805  1.117164
H -5.671111 -0.175748  1.528568
C -4.449152 -0.605207 -0.183510
H -5.278607 -0.946095 -0.790475
C -3.164472 -0.602405 -0.709886
H -2.992542 -0.936608 -1.726140
C -0.625693 -2.438043 -1.088771
C -0.838681 -2.885410 -2.527276
H -1.545739 -2.553968 -0.510839
H  0.135050 -3.077694 -0.630757
H -1.603915 -2.279659 -3.015793
H -1.157438 -3.928219 -2.553887
H  0.086671 -2.791594 -3.097835
F  0.788838 -0.545622  1.875349
N  4.811152 -3.027416  1.203269
H -0.734658  4.482412 -1.008469
O  5.827271 -3.175887  0.559322
O  4.481475 -3.740630  2.126750

43
Id
C -3.645166  0.614785  0.470432
```

```
C -3.714275 -0.396446 -0.471819
C -2.586762 -0.657988 -1.228979
C -1.447328  0.090360 -1.009272
C -1.355830  1.102266 -0.057948
C -2.499366  1.363317  0.684982
C -0.127781  1.918199  0.126352
C  1.141292  1.363977  0.359157
C  1.353924 -0.106979  0.547252
N  0.493981 -0.744299  1.227799
C -0.258464  3.304149  0.046539
C  0.839971  4.138989  0.189457
C  2.095889  3.591204  0.404733
C  2.239424  2.213963  0.478432
H -2.580956 -1.426062 -1.990423
H -2.488715  2.137155  1.439934
H -1.236266  3.729172 -0.146909
H  0.713264  5.212034  0.121873
H  3.219808  1.785980  0.650802
C  2.590076 -0.675868 -0.078423
C  2.853135 -0.453201 -1.429357
H  2.156214  0.126424 -2.022889
C  3.999768 -0.971376 -2.014822
H  4.189005 -0.804946 -3.067937
C  4.907728 -1.693652 -1.249941
H  5.807015 -2.089221 -1.705541
C  4.664001 -1.897498  0.102385
H  5.374977 -2.446739  0.706973
C  3.508211 -1.394918  0.684178
H  3.321457 -1.549628  1.739866
C  0.548451 -2.181528  1.423593
C  0.729975 -2.509130  2.898765
H  1.320198 -2.671156  0.824983
H -0.420702 -2.568651  1.095221
H  1.684348 -2.129887  3.268317
H  0.707615 -3.589276  3.049046
H -0.067941 -2.061719  3.493397
F -0.377253 -0.171917 -1.771433
N -4.833084  0.905351  1.279721
H  2.961762  4.231674  0.515189
O -4.755444  1.780022  2.116140
O -5.835544  0.255202  1.071622
H -4.624321 -0.960386 -0.614066

45
Ie
C  4.323037 -1.021020  0.059996
C  3.946361 -1.883521  1.089102
C  2.739194 -1.695936  1.736353
C  1.919227 -0.655665  1.345266
C  2.252552  0.215420  0.324056
C  3.482106  0.022202 -0.312601
C  1.384497  1.357622 -0.067976
C  0.029951  1.224993 -0.414743
C -0.649998 -0.106805 -0.507293
N -0.014003 -1.062013 -1.047037
C  1.958694  2.629538 -0.073884
C  1.220944  3.754131 -0.411742
C -0.119683  3.622969 -0.742577
C -0.705841  2.366506 -0.732493
O  5.521171 -1.271921 -0.522962
H  2.428754 -2.343872  2.546056
H  3.752075  0.692927 -1.116155
H  2.999623  2.734500  0.208135
H  1.691315  4.729357 -0.405845
H -1.752563  2.261838 -0.992912
C -2.046032 -0.165447  0.033900
C -2.314594  0.281659  1.326509
H -1.506547  0.657531  1.942471
C -3.608718  0.244655  1.826746
H -3.805213  0.581218  2.837086
C -4.651482 -0.213230  1.030413
H -5.662545 -0.231544  1.417911
C -4.394554 -0.637685 -0.267055
H -5.204424 -0.982581 -0.897839
C -3.097286 -0.617739 -0.761307
H -2.897402 -0.942741 -1.775327
C -0.531550 -2.416299 -1.114558
C -0.710924 -2.847799 -2.562672
H -1.461060 -2.553367 -0.556573
H  0.229402 -3.051551 -0.651130
H -1.471321 -2.242603 -3.059556
H -1.020105 -3.892988 -2.607829
H  0.225058 -2.740628 -3.113151
F  0.749148 -0.485992  2.003848
H  4.609087 -2.690335  1.373946
C  5.937509 -0.412801 -1.570301
H  6.910950 -0.775336 -1.891701
H  6.033007  0.618012 -1.219981
```

```
H  5.240298 -0.449950 -2.411132
H -0.708204  4.493040 -1.004735

45
If
C  4.268223 -1.078092 -0.128003
C  3.876292 -1.909381  0.903833
C  2.695317 -1.728303  1.603368
C  1.898817 -0.675834  1.219660
C  2.225030  0.189555  0.179403
C  3.434890 -0.027937 -0.476663
C  1.359592  1.337754 -0.189322
C -0.012765  1.208827 -0.463746
C -0.701805 -0.121491 -0.520161
N -0.069763 -1.073751 -1.066732
C  1.955542  2.598621 -0.241293
C  1.221607  3.730224 -0.547234
C -0.129549  3.576497 -0.793657
C -0.756303  2.346365 -0.752143
H  5.198678 -1.249795 -0.648522
N  4.743003 -3.032724  1.284141
H  2.402173 -2.375763  2.417225
H  3.715264  0.629767 -1.289458
H  3.010414  2.695768 -0.019118
H  1.683889  4.705969 -0.581286
H -1.814816  2.271374 -0.959445
C -2.078666 -0.167973  0.063547
C -2.304010  0.330449  1.346260
H -1.477031  0.735871  1.917033
C -3.578870  0.307508  1.893429
H -3.741474  0.685834  2.894886
C -4.645299 -0.188194  1.153162
H -5.642667 -0.195919  1.575018
C -4.431716 -0.663803 -0.134291
H -5.262097 -1.037533 -0.720125
C -3.153743 -0.656970 -0.676309
H -2.991052 -1.018783 -1.684171
C -0.578154 -2.431766 -1.125240
C -0.796844 -2.851306 -2.571471
H -1.488606 -2.578262 -0.539477
H  0.202496 -3.063297 -0.690551
H -1.571447 -2.243215 -3.041814
H -1.104652 -3.896669 -2.616103
H  0.123341 -2.736543 -3.146178
F  0.757211 -0.475639  1.895257
N -0.933360  4.765852 -1.111555
O -2.109953  4.609060 -1.355165
O -0.375780  5.841839 -1.113063
O  4.374743 -3.753351  2.186340
O  5.779597 -3.180750  0.674484

47
Ig
C  4.320966 -1.009652  0.071715
C  3.925947 -1.882509  1.085677
C  2.703428 -1.709955  1.706469
C  1.884398 -0.673559  1.303841
C  2.237666  0.206940  0.297559
C  3.482684  0.030955 -0.312909
C  1.373928  1.347744 -0.100889
C  0.017028  1.213176 -0.443916
C -0.669399 -0.117972 -0.532182
N -0.032295 -1.062511 -1.085750
C  1.956500  2.617359 -0.113292
C  1.231194  3.745611 -0.448736
C -0.104691  3.583694 -0.765323
C -0.720901  2.347606 -0.760075
O  5.533532 -1.248308 -0.483442
H  2.379146 -2.365690  2.504264
H  3.767226  0.711042 -1.103627
H  2.996606  2.721406  0.167603
H  1.686856  4.725139 -0.450755
H -1.767698  2.265713 -1.019057
C -2.053373 -0.171660  0.035732
C -2.298239  0.338410  1.310301
H -1.482395  0.760235  1.884879
C -3.578206  0.308651  1.845039
H -3.754080  0.696858  2.840517
C -4.632084 -0.206705  1.100165
H -5.633081 -0.219778  1.513246
C -4.400401 -0.692556 -0.180169
H -5.220497 -1.081310 -0.770882
C -3.116961 -0.677923 -0.709579
H -2.942849 -1.046112 -1.713223
C -0.537542 -2.421072 -1.160252
C -0.755462 -2.829105 -2.609822
H -1.447270 -2.575771 -0.575588
H  0.243974 -3.055012 -0.731252
```

```
H -1.532516 -2.220322 -3.075303
H -1.060142 -3.875065 -2.663718
H  0.163509 -2.706871 -3.185026
F  0.699885 -0.514079  1.935620
N -0.901923  4.766816 -1.114788
O -2.072041  4.606094 -1.387782
O -0.347640  5.844988 -1.111676
H  4.589129 -2.685409  1.380461
C  5.986466 -0.356330 -1.487482
H  6.969914 -0.711354 -1.785705
H  6.069845  0.661893 -1.099215
H  5.320061 -0.365600 -2.353475

41
Ih
C  4.241311 -1.079541 -0.443267
C  4.073300 -1.938499  0.633514
C  3.007945 -1.751894  1.502018
C  2.115249 -0.714125  1.278897
C  2.248679  0.154979  0.197520
C  3.340320 -0.046348 -0.648408
C  1.352533  1.313410 -0.074954
C -0.015972  1.209200 -0.376771
C -0.721396 -0.110408 -0.444860
N -0.082648 -1.103975 -0.907791
C  1.944737  2.576506 -0.059751
C  1.215758  3.722834 -0.339141
C -0.133540  3.619519 -0.641292
C -0.738169  2.371891 -0.648775
H  5.070861 -1.213220 -1.125565
H  4.769261 -2.749469  0.805678
H  2.869002 -2.402399  2.355612
H  3.464716  0.619313 -1.494018
H  2.997716  2.655461  0.182925
H  1.702145  4.690034 -0.319720
H -0.714515  4.503891 -0.870253
H -1.791819  2.292578 -0.887107
C -2.139874 -0.120021  0.040763
C -2.464994  0.452271  1.270123
H -1.685172  0.897177  1.876503
C -3.778554  0.462318  1.717093
H -4.016196  0.902178  2.677657
C -4.786805 -0.077715  0.927781
H -5.813596 -0.061095  1.271250
C -4.475166 -0.627319 -0.309086
H -5.258402 -1.036223 -0.935208
C -3.158427 -0.650163 -0.749285
H -2.920150 -1.069680 -1.719360
C -0.604965 -2.457114 -0.906809
C -0.721393 -2.984978 -2.329139
H -1.557244 -2.557148 -0.380255
H  0.133676 -3.060297 -0.369320
H -1.457120 -2.414164 -2.898681
H -1.029309 -4.031409 -2.319997
H  0.239174 -2.911593 -2.841500
Cl  0.808384 -0.512464  2.411039

41
Ia_vac
C  2.789112  0.250301 -1.113704
C  3.379399 -0.815949 -0.451805
C  2.605381 -1.645208  0.347761
C  1.255326 -1.389741  0.459305
C  0.623582 -0.339596 -0.193351
C  1.427819  0.482334 -0.978730
C -0.824422 -0.061534 -0.018704
C -1.827247 -1.019894 -0.232277
C -1.519943 -2.380489 -0.773733
N -0.648492 -2.463265 -1.688881
C -1.193055  1.212452  0.408675
C -2.521438  1.545230  0.626857
C -3.508394  0.591672  0.433737
C -3.155186 -0.681956  0.016194
H  3.386378  0.899376 -1.739953
H  4.439984 -1.004487 -0.552001
H  3.029689 -2.479983  0.889071
H  0.961920  1.303777 -1.509045
H -0.415845  1.944210  0.592729
H -2.780449  2.541899  0.960160
H -4.548352  0.835910  0.607122
H -3.922604 -1.431315 -0.136092
C -2.295811 -3.515274 -0.178671
C -2.334874 -3.678939  1.204275
H -1.796564 -2.982483  1.834965
C -3.049807 -4.725363  1.766865
H -3.062115 -4.850987  2.841880
C -3.753346 -5.605352  0.955047
H -4.316720 -6.418167  1.395004
```

C -3.739402 -5.434598 -0.422041
H -4.297172 -6.109108 -1.058868
C -3.011913 -4.395912 -0.985516
H -3.004936 -4.257965 -2.059915
C -0.197345 -3.724814 -2.237671
C -0.529519 -3.800447 -3.722148
H -0.588134 -4.598488 -1.708444
H 0.890709 -3.728436 -2.122321
H -1.608833 -3.812328 -3.881411
H -0.107301 -4.703857 -4.163093
H -0.123877 -2.932470 -4.241304
F 0.525168 -2.199446 1.248589

41
IIa_vac
C 2.407433 0.558675 -1.790960
C 2.835734 -0.798481 -1.878380
C 2.156863 -1.802096 -1.290065
C 0.985431 -1.539555 -0.419885
C 0.431557 -0.173224 -0.570865
C 1.235924 0.833890 -1.162175
C -0.852723 0.076405 -0.073865
C -1.720956 -1.021612 0.217871
C -1.293064 -2.317420 -0.094067
N -0.039997 -2.572953 -0.480670
C -1.384155 1.389900 0.122314
C -2.642923 1.581234 0.595935
C -3.493788 0.483282 0.897090
C -3.042363 -0.781686 0.703112
H 2.981748 1.342158 -2.264086
H 3.729008 -1.035445 -2.443944
H 0.870896 1.851545 -1.160608
H -0.759303 2.249318 -0.070618
H -3.002422 2.590014 0.757433
H -4.490285 0.659643 1.278062
H -3.682028 -1.626068 0.923180
C -2.271645 -3.441145 -0.041442
C -2.407390 -4.192329 1.122912
H -1.778558 -3.967480 1.976475
C -3.343940 -5.213467 1.186741
H -3.447695 -5.790980 2.096144
C -4.149565 -5.489026 0.089544
H -4.880335 -6.285729 0.140407
C -4.022649 -4.735511 -1.068697
H -4.655279 -4.940651 -1.922528
C -3.089613 -3.709568 -1.132789
H -3.000286 -3.104511 -2.027088
C 0.379421 -3.950420 -0.794990
C 0.283916 -4.274281 -2.277562
H -0.247883 -4.629679 -0.226444
H 1.389766 -4.076808 -0.415478
H -0.751325 -4.210344 -2.613230
H 0.637477 -5.290795 -2.455160
H 0.882170 -3.583888 -2.869823
F 1.469251 -1.678793 0.963627
H 2.537105 -2.808748 -1.362270

41
TSa
C 3.688625 -1.327536 -1.471071
C 3.490170 -2.264000 -0.450420
C 2.677551 -1.996766 0.628872
C 1.982853 -0.761438 0.709418
C 2.213725 0.208727 -0.305817
C 3.051930 -0.099918 -1.373197
C 1.412558 1.427251 -0.241064
C 0.068325 1.349661 0.182786
C -0.567374 0.036726 0.395681
N 0.143809 -0.987964 0.688931
C 1.921655 2.671392 -0.630283
C 1.147381 3.812962 -0.551109
C -0.167281 3.741594 -0.090048
C -0.704458 2.515989 0.250267
H 4.331816 -1.552244 -2.310684
H 4.003776 -3.218166 -0.489121
H 2.568354 -2.705504 1.439755
H 3.178242 0.640967 -2.155554
H 2.949096 2.734702 -0.968487
H 1.569409 4.769015 -0.835994
H -0.768402 4.637857 -0.009053
H -1.734966 2.453424 0.577159
C -2.043167 -0.058744 0.179832
C -2.890669 -0.367437 1.240305
H -2.475393 -0.547389 2.225121
C -4.262461 -0.434690 1.035141
H -4.918756 -0.668439 1.863964
C -4.790167 -0.202846 -0.228445
H -5.859839 -0.259862 -0.386887

```
C -3.945487  0.107186 -1.287761
H -4.353691  0.292013 -2.273447
C -2.575737  0.190590 -1.082512
H -1.914512  0.446633 -1.902234
C -0.347668 -2.359080  0.679932
C -0.371276 -2.918269 -0.736421
H -1.337329 -2.406951  1.129661
H  0.330134 -2.943538  1.302908
H -1.088343 -2.374269 -1.353237
H -0.661264 -3.969356 -0.714172
H  0.614526 -2.839417 -1.197357
F  1.913985 -0.250865  2.014293

43
TSb
C  3.855006 -0.830809 -1.322603
C  3.695336 -1.877321 -0.402832
C  2.800751 -1.795486  0.637811
C  1.979822 -0.646416  0.777966
C  2.162803  0.432588 -0.131343
C  3.095252  0.312594 -1.163247
C  1.238144  1.546290 -0.024339
C -0.114117  1.295411  0.310584
C -0.632450 -0.086179  0.359566
N  0.160149 -1.060604  0.598603
C  1.635563  2.864629 -0.296367
C  0.755698  3.914610 -0.177355
C -0.555512  3.643268  0.205510
C -1.005948  2.360345  0.425823
H  4.565366 -0.913676 -2.133386
H  4.308464 -2.766833 -0.492317
H  2.720414 -2.582381  1.376600
H  3.192411  1.132693 -1.866387
H  2.665159  3.057681 -0.568748
H  1.070918  4.932507 -0.356943
N -1.493689  4.754000  0.356445
H -2.043002  2.189866  0.678466
C -2.079430 -0.291440  0.048710
C -2.937009 -0.781361  1.029337
H -2.549313 -1.017987  2.013157
C -4.285061 -0.956939  0.747330
H -4.949426 -1.331941  1.515579
C -4.777443 -0.652375 -0.515251
H -5.827988 -0.794659 -0.735653
C -3.923124 -0.159735 -1.494124
H -4.305382  0.082438 -2.477690
C -2.577949  0.031832 -1.211217
H -1.911459  0.429026 -1.968128
C -0.186815 -2.464192  0.427496
C -0.077118 -2.870269 -1.036272
H -1.189652 -2.653468  0.805415
H  0.513931 -3.041351  1.031725
H -0.810931 -2.332888 -1.639098
H -0.261774 -3.940347 -1.136508
H  0.919237 -2.650305 -1.423209
F  1.764135 -0.274205  2.104275
O -1.104608  5.868910  0.074575
O -2.614800  4.510479  0.753469

43
TSc
C  3.814647 -0.828608 -1.349847
C  3.645051 -1.835362 -0.409385
C  2.776385 -1.765770  0.655788
C  1.959478 -0.618488  0.784910
C  2.137359  0.445943 -0.144380
C  3.045913  0.315691 -1.183384
C  1.214125  1.575085 -0.018541
C -0.137189  1.322535  0.295794
C -0.646942 -0.063445  0.352328
N  0.147574 -1.031193  0.613603
C  1.624113  2.891125 -0.238952
C  0.733359  3.941188 -0.104806
C -0.593380  3.695554  0.241257
C -1.026766  2.394483  0.416918
H  4.510449 -0.937266 -2.166207
N  4.461480 -3.056034 -0.541625
H  2.724717 -2.551071  1.394540
H  3.131618  1.123999 -1.900147
H  2.660137  3.083385 -0.490638
H  1.073913  4.957543 -0.259002
H -2.064648  2.202665  0.658175
C -2.089606 -0.284017  0.034283
C -2.952460 -0.773246  1.010679
H -2.570744 -1.002696  1.998638
C -4.297611 -0.956009  0.719729
H -4.966170 -1.328951  1.485308
C -4.781969 -0.660318 -0.548047
```

```
H -5.830258 -0.808427 -0.775111
C -3.921591 -0.170512 -1.523411
H -4.296578  0.064138 -2.511582
C -2.579675  0.028454 -1.231325
H -1.909041  0.423606 -1.985519
C -0.183448 -2.440338  0.460159
C -0.037473 -2.866774 -0.995205
H -1.191642 -2.635616  0.819927
H  0.510393 -3.001223  1.087930
H -0.754640 -2.335974 -1.623547
H -0.219820 -3.938043 -1.086265
H  0.968416 -2.652899 -1.360697
F  1.760847 -0.229833  2.106254
O  5.309388 -3.084420 -1.408379
O  4.245786 -3.975751  0.219396
H -1.286676  4.517510  0.364289

43
TSd
C  3.393822 -0.228822 -0.271770
C  3.266842 -1.165370  0.756889
C  2.209872 -1.088203  1.625998
C  1.230793 -0.081473  1.454893
C  1.384271  0.885045  0.421286
C  2.456115  0.792963 -0.431858
C  0.280670  1.834249  0.215819
C -1.046715  1.364217  0.240917
C -1.341607 -0.082937  0.385013
N -0.487910 -0.860208  0.924813
C  0.523521  3.178885 -0.054804
C -0.525365  4.060237 -0.261273
C -1.838142  3.605242 -0.208820
C -2.092909  2.263967  0.021443
N  4.507831 -0.310419 -1.169441
H  2.118358 -1.769056  2.461249
H  2.557753  1.497531 -1.246497
H  1.547376  3.532152 -0.082989
H -0.320018  5.105340 -0.456862
H -3.115479  1.908349  0.033958
C -2.641017 -0.583264 -0.160543
C -3.637804 -1.025549  0.703958
H -3.466036 -1.009658  1.773806
C -4.847277 -1.477886  0.193468
H -5.622585 -1.816408  0.869205
C -5.060057 -1.497035 -1.178961
H -6.001660 -1.855852 -1.575505
C -4.065732 -1.053151 -2.042476
H -4.230117 -1.065035 -3.112522
C -2.860859 -0.587100 -1.535222
H -2.087678 -0.228715 -2.205013
C -0.590936 -2.309166  0.966227
C -0.086603 -2.917101 -0.336058
H -1.617104 -2.616477  1.164708
H  0.027292 -2.646707  1.800166
H -0.727016 -2.624478 -1.169525
H -0.087452 -4.005045 -0.261028
H  0.931353 -2.584594 -0.546394
F  0.662271  0.362091  2.617483
O  4.616645  0.532900 -2.047339
O  5.310362 -1.221033 -1.027185
H -2.660235  4.293273 -0.357695
H  4.022470 -1.928094  0.883338

43
TSe
C  3.393822 -0.228822 -0.271770
C  3.266842 -1.165370  0.756889
C  2.209872 -1.088203  1.625998
C  1.230793 -0.081473  1.454893
C  1.384271  0.885045  0.421286
C  2.456115  0.792963 -0.431858
C  0.280670  1.834249  0.215819
C -1.046715  1.364217  0.240917
C -1.341607 -0.082937  0.385013
N -0.487910 -0.860208  0.924813
C  0.523521  3.178885 -0.054804
C -0.525365  4.060237 -0.261273
C -1.838142  3.605242 -0.208820
C -2.092909  2.263967  0.021443
N  4.507831 -0.310419 -1.169441
H  2.118358 -1.769056  2.461249
H  2.557753  1.497531 -1.246497
H  1.547376  3.532152 -0.082989
H -0.320018  5.105340 -0.456862
H -3.115479  1.908349  0.033958
C -2.641017 -0.583264 -0.160543
C -3.637804 -1.025549  0.703958
H -3.466036 -1.009658  1.773806
```

```
C -4.847277 -1.477886  0.193468
H -5.622585 -1.816408  0.869205
C -5.060057 -1.497035 -1.178961
H -6.001660 -1.855852 -1.575505
C -4.065732 -1.053151 -2.042476
H -4.230117 -1.065035 -3.112522
C -2.860859 -0.587100 -1.535222
H -2.087678 -0.228715 -2.205013
C -0.590936 -2.309166  0.966227
C -0.086603 -2.917101 -0.336058
H -1.617104 -2.616477  1.164708
H  0.027292 -2.646707  1.800166
H -0.727016 -2.624478 -1.169525
H -0.087452 -4.005045 -0.261028
H  0.931353 -2.584594 -0.546394
F  0.662271  0.362091  2.617483
O  4.616645  0.532900 -2.047339
O  5.310362 -1.221033 -1.027185
H -2.660235  4.293273 -0.357695
H  4.022470 -1.928094  0.883338

45
TSf
C  3.841898 -0.802330 -1.332981
C  3.663303 -1.827573 -0.411609
C  2.789693 -1.779025  0.647367
C  1.974185 -0.632231  0.793696
C  2.156008  0.446351 -0.116494
C  3.075318  0.337069 -1.152394
C  1.228668  1.566208  0.000400
C -0.127097  1.308156  0.300410
C -0.644508 -0.078153  0.349209
N  0.148278 -1.046462  0.602196
C  1.641026  2.882765 -0.232727
C  0.758300  3.934405 -0.120770
C -0.560359  3.656576  0.217104
C -1.020997  2.371428  0.406009
H  4.542298 -0.897780 -2.146946
N  4.484993 -3.045695 -0.555818
H  2.734312 -2.578659  1.370605
H  3.165488  1.157866 -1.854200
H  2.678088  3.076108 -0.474523
H  1.080137  4.954343 -0.275016
N -1.503303  4.769808  0.359531
H -2.064884  2.200284  0.627976
C -2.089321 -0.287434  0.034358
C -2.953242 -0.759664  1.017890
H -2.571694 -0.981387  2.007593
C -4.299836 -0.935541  0.729523
H -4.971568 -1.294827  1.498848
C -4.782762 -0.650017 -0.541075
H -5.832330 -0.793178 -0.765578
C -3.920780 -0.176229 -1.522859
H -4.295808  0.049956 -2.512934
C -2.577389  0.016894 -1.234147
H -1.902967  0.397737 -1.992518
C -0.192495 -2.454484  0.456811
C -0.089472 -2.881342 -1.001433
H -1.190870 -2.642959  0.846646
H  0.516772 -3.017787  1.064886
H -0.825415 -2.352581 -1.608995
H -0.272696 -3.952847 -1.085355
H  0.904934 -2.666739 -1.396758
F  1.758593 -0.270235  2.113931
O -1.106032  5.885903  0.099292
O -2.631476  4.521132  0.728594
O  5.337542 -3.057227 -1.417453
O  4.266648 -3.975321  0.191018

47
TSg
C  3.965520 -0.822374 -1.140658
C  3.750036 -1.898626 -0.259962
C  2.805588 -1.838508  0.728862
C  1.982534 -0.687055  0.866490
C  2.211654  0.403015 -0.008241
C  3.207396  0.318195 -1.000549
C  1.289198  1.513065  0.072692
C -0.077972  1.264704  0.357552
C -0.606449 -0.109777  0.377967
N  0.168144 -1.093237  0.644928
C  1.694677  2.835211 -0.187029
C  0.816599  3.885950 -0.087196
C -0.509387  3.616379  0.251304
C -0.969746  2.333534  0.443851
O  4.937452 -1.010263 -2.083045
H  2.689480 -2.648565  1.436787
H  3.328586  1.156933 -1.673477
```

```
H  2.732754  3.027638 -0.425781
H  1.140594  4.903946 -0.249933
N -1.447598  4.726519  0.382105
H -2.016777  2.164907  0.653248
C -2.042640 -0.299921  0.010712
C -2.953852 -0.726632  0.972679
H -2.613070 -0.932982  1.980570
C -4.294236 -0.873384  0.640707
H -5.001952 -1.197628  1.393251
C -4.724950 -0.604384 -0.651932
H -5.770157 -0.720834 -0.910131
C -3.815715 -0.178874 -1.613292
H -4.149301  0.033982 -2.621119
C -2.478444 -0.015902 -1.281118
H -1.768297  0.330128 -2.023318
C -0.191519 -2.492803  0.463257
C -0.054022 -2.910065 -0.994771
H -1.205483 -2.665198  0.821083
H  0.485061 -3.078338  1.087037
H -0.774045 -2.378609 -1.618629
H -0.237325 -3.980916 -1.089769
H  0.950118 -2.695544 -1.364173
F  1.731359 -0.346269  2.199451
O -1.039361  5.846997  0.150993
O -2.590095  4.480751  0.713373
H  4.373047 -2.779789 -0.359972
C  5.229363  0.084303 -2.926335
H  6.048886 -0.229615 -3.569096
H  5.539209  0.959002 -2.346604
H  4.368098  0.350963 -3.546349

41
TSh
C  3.865304 -0.780445 -1.392733
C  3.702051 -1.861446 -0.524943
C  2.814853 -1.818082  0.531237
C  2.006986 -0.672106  0.739073
C  2.178318  0.435932 -0.135074
C  3.098610  0.354685 -1.175159
C  1.251705  1.560110 -0.006798
C -0.106162  1.306955  0.273393
C -0.616199 -0.078135  0.319258
N  0.172282 -1.049406  0.579015
C  1.662184  2.881402 -0.203325
C  0.768485  3.929567 -0.079659
C -0.566747  3.680734  0.231717
C -1.000649  2.377490  0.384916
H  4.572241 -0.823691 -2.209692
H  2.755293 -2.641083  1.230401
H  3.180454  1.202469 -1.847119
H  2.703453  3.079484 -0.427972
H  1.112800  4.947671 -0.214026
H -2.043924  2.181579  0.598524
C -2.063795 -0.294891  0.016304
C -2.929204 -0.721763  1.019064
H -2.546032 -0.906810  2.015984
C -4.279083 -0.894730  0.743188
H -4.950386 -1.218112  1.528719
C -4.765269 -0.650717 -0.534788
H -5.817405 -0.789232 -0.750280
C -3.901453 -0.224353 -1.536682
H -4.278236 -0.032241 -2.533501
C -2.554666 -0.035598 -1.260518
H -1.880417  0.310516 -2.035298
C -0.179765 -2.457514  0.494889
C -0.113615 -2.957176 -0.942072
H -1.170631 -2.621020  0.915266
H  0.537383 -2.997829  1.115188
H -0.867273 -2.462804 -1.556773
H -0.298049 -4.031802 -0.966353
H  0.869793 -2.763055 -1.372851
Cl  1.833470 -0.266015  2.553948
H -1.264638  4.500138  0.344528
H  4.302414 -2.754835 -0.656414

41
TSa_vac
C  3.672419 -1.361316 -1.418561
C  3.462398 -2.282027 -0.381983
C  2.637689 -2.004212  0.678083
C  1.937185 -0.761250  0.745028
C  2.191983  0.193645 -0.288052
C  3.044024 -0.133854 -1.341357
C  1.405070  1.409760 -0.243111
C  0.065171  1.350187  0.204174
C -0.563169  0.047138  0.420354
N  0.147445 -0.988698  0.700219
C  1.904248  2.645984 -0.677860
```

```
C  1.134434  3.786629 -0.613722
C -0.174928  3.732529 -0.127602
C -0.706289  2.521048  0.253515
H  4.322324 -1.601520 -2.247234
H  3.984513 -3.231674 -0.397274
H  2.541090 -2.689632  1.509566
H  3.181227  0.594944 -2.132586
H  2.928451  2.699274 -1.025139
H  1.553238  4.734614 -0.927533
H -0.770176  4.632960 -0.058818
H -1.731301  2.465954  0.598127
C -2.038536 -0.055393  0.207330
C -2.878172 -0.378359  1.269479
H -2.452065 -0.560350  2.249185
C -4.250315 -0.452395  1.075969
H -4.898664 -0.695904  1.907540
C -4.789137 -0.211604 -0.180122
H -5.859098 -0.273167 -0.330526
C -3.955168  0.115804 -1.241178
H -4.372766  0.310349 -2.220414
C -2.585035  0.205083 -1.045890
H -1.930228  0.480769 -1.863926
C -0.340060 -2.357354  0.606101
C -0.303287 -2.842416 -0.838800
H -1.347136 -2.427025  1.012654
H  0.316239 -2.971113  1.223163
H -1.002180 -2.269032 -1.449191
H -0.583594 -3.894799 -0.889106
H  0.700521 -2.725926 -1.249166
F  1.881314 -0.235647  2.028317

41
IIIa
C  2.562625  0.628317 -1.558069
C  2.936598 -0.692104 -1.821308
C  2.070314 -1.726855 -1.563122
C  0.798794 -1.463143 -1.027614
C  0.409819 -0.139736 -0.759727
C  1.320659  0.894692 -1.038784
C -0.904139  0.115656 -0.214111
C -1.757371 -0.980359  0.010135
C -1.298242 -2.304925 -0.279707
N -0.093215 -2.517600 -0.761001
C -1.371068  1.403834  0.095422
C -2.636466  1.584356  0.600564
C -3.486835  0.491632  0.818415
C -3.054680 -0.776156  0.527259
H  3.248673  1.439399 -1.764195
H  3.912957 -0.910097 -2.233913
H  1.041877  1.920016 -0.844397
H -0.740682  2.266911 -0.057212
H -2.979723  2.584745  0.832431
H -4.480178  0.648753  1.217320
H -3.705708 -1.622563  0.694876
C -2.227664 -3.448075 -0.065435
C -2.300188 -4.068847  1.178158
H -1.629352 -3.741959  1.964578
C -3.211605 -5.097519  1.372313
H -3.268054 -5.586432  2.336769
C -4.048576 -5.498379  0.337759
H -4.758905 -6.300266  0.495742
C -3.978327 -4.867724 -0.897390
H -4.632650 -5.172796 -1.704180
C -3.069319 -3.837704 -1.102052
H -3.019620 -3.331696 -2.058776
C  0.371614 -3.906634 -1.003648
C  0.245117 -4.298168 -2.463973
H -0.225246 -4.559626 -0.378310
H  1.392645 -3.970903 -0.638882
H -0.799993 -4.279159 -2.773934
H  0.622846 -5.313325 -2.587683
H  0.812367 -3.637707 -3.119141
F  0.305309 -2.622325  2.182982
H  2.389121 -2.732158 -1.784999

43
IIIb
C  4.253646  2.268461 -0.132055
C  4.705211  0.946892 -0.095906
C  3.812436 -0.097851 -0.060871
C  2.433053  0.158751 -0.062218
C  1.965509  1.484279 -0.101124
C  2.906279  2.528425 -0.134657
C  0.543016  1.730600 -0.109397
C -0.322011  0.621924 -0.093308
C  0.215904 -0.706616 -0.035602
N  1.512589 -0.906218 -0.021266
C -0.011169  3.024025 -0.135784
```

```
C -1.369164  3.205621 -0.147469
C -2.201127  2.083287 -0.138810
C -1.715341  0.806778 -0.113868
H  4.962884  3.085027 -0.157611
H  5.766133  0.734479 -0.094844
H  2.568889  3.553809 -0.161533
H  0.626111  3.894808 -0.143325
H -1.795788  4.197975 -0.163861
N -3.658125  2.282330 -0.154904
H -2.391540 -0.035026 -0.105595
C -0.721710 -1.861263 -0.029228
C -1.275019 -2.296239  1.171756
H -0.972369 -1.807379  2.090528
C -2.179688 -3.348551  1.159856
H -2.607903 -3.695783  2.091487
C -2.536443 -3.953233 -0.039643
H -3.244207 -4.772597 -0.042908
C -1.991966 -3.503316 -1.235316
H -2.275070 -3.966379 -2.171846
C -1.083458 -2.452939 -1.235305
H -0.663821 -2.090334 -2.165574
C  2.048839 -2.287998  0.090883
C  2.500024 -2.830730 -1.252079
H  1.262823 -2.904005  0.511128
H  2.851145 -2.256429  0.822119
H  1.651390 -2.933935 -1.928020
H  2.939741 -3.816201 -1.098025
H  3.244528 -2.192338 -1.726219
F  0.661116 -0.470724  2.807003
H  4.197852 -1.103751 -0.037741
O -4.068611  3.421865 -0.156640
O -4.367262  1.300926 -0.166352

43
IIIc
C  3.689492  1.134599 -0.120740
C  3.543293 -0.248864 -0.105962
C  2.325869 -0.874664 -0.087948
C  1.169096 -0.086040 -0.081386
C  1.272553  1.315508 -0.102339
C  2.552353  1.898555 -0.121474
C  0.065429  2.111753 -0.103680
C -1.171517  1.438883 -0.100120
C -1.201412  0.006690 -0.067176
N -0.093958 -0.698731 -0.054017
C  0.069951  3.514591 -0.118614
C -1.115055  4.213053 -0.134431
C -2.344629  3.542447 -0.139293
C -2.375713  2.171120 -0.122447
H  4.670373  1.585627 -0.133539
N  4.752248 -1.089744 -0.107729
H  2.652850  2.972683 -0.136171
H  1.000351  4.062612 -0.116649
H -1.094856  5.295328 -0.145330
H -3.322759  1.650673 -0.127134
C -2.517821 -0.685012 -0.058294
C -3.203720 -0.845321  1.141999
H -2.760306 -0.493532  2.065110
C -4.445724 -1.463770  1.140834
H -4.977622 -1.596511  2.074122
C -5.004512 -1.907765 -0.051578
H -5.974567 -2.388284 -0.048174
C -4.323487 -1.728984 -1.248462
H -4.760780 -2.064760 -2.179895
C -3.077863 -1.115117 -1.256503
H -2.546761 -0.963060 -2.187959
C -0.149877 -2.180495  0.035842
C  0.039681 -2.834147 -1.320156
H -1.112108 -2.439788  0.460942
H  0.601830 -2.482248  0.758613
H -0.784353 -2.581870 -1.987145
H  0.055926 -3.915512 -1.184763
H  0.972421 -2.533242 -1.796351
F -0.089032 -0.574830  2.762905
H  2.298320 -1.950928 -0.079684
O  5.823444 -0.528652 -0.049220
O  4.612147 -2.291106 -0.167463
H -3.268338  4.105167 -0.155984

43
IIId
C  3.681182 -0.476927 -0.046466
C  3.179551 -1.773408  0.016224
C  1.820144 -1.948863  0.027945
C  0.957537 -0.839838 -0.020381
C  1.480660  0.463212 -0.087040
C  2.873240  0.624951 -0.098556
C  0.581924  1.594055 -0.149681
```

```
C -0.801323  1.334815 -0.158860
C -1.269184 -0.014314 -0.065180
N -0.432389 -1.028012 -0.000369
C  1.019510  2.925354 -0.207151
C  0.108111  3.952716 -0.282694
C -1.268543  3.693487 -0.306336
C -1.720983  2.400602 -0.244956
N  5.140122 -0.280852 -0.059659
H  3.320625  1.604872 -0.148735
H  2.073156  3.161796 -0.190345
H  0.461602  4.974970 -0.325657
H -2.782098  2.196844 -0.260298
C -2.735652 -0.264000 -0.070909
C -3.458157 -0.165089  1.114762
H -2.925440  0.037315  2.035991
C -4.833440 -0.347609  1.089845
H -5.398497 -0.276954  2.010505
C -5.483337 -0.616482 -0.108983
H -6.556944 -0.755437 -0.123219
C -4.757714 -0.702240 -1.289784
H -5.261988 -0.903643 -2.226201
C -3.380265 -0.524556 -1.275499
H -2.810459 -0.578191 -2.195015
C -0.952105 -2.409569  0.171290
C -0.985987 -3.172154 -1.139680
H -1.945875 -2.325881  0.594643
H -0.331657 -2.891077  0.921316
H -1.682102 -2.705608 -1.836456
H -1.325939 -4.188497 -0.940861
H -0.006406 -3.224698 -1.613603
F -0.749818 -0.308291  2.750259
H  1.439997 -2.955758  0.070932
O  5.562093  0.851998 -0.129717
O  5.839586 -1.267485  0.000286
H -1.972811  4.512050 -0.370542
H  3.845340 -2.622796  0.053685

45
IIIe
C  3.886118 -0.783302  0.037445
C  3.254333 -2.034283  0.154679
C  1.891648 -2.126016  0.158219
C  1.098159 -0.967494  0.045328
C  1.719454  0.284996 -0.073532
C  3.124619  0.358186 -0.076395
C  0.894859  1.466829 -0.193258
C -0.504282  1.306815 -0.208005
C -1.070642 -0.000795 -0.078888
N -0.301640 -1.059439  0.048397
C  1.421567  2.765515 -0.302249
C  0.587594  3.850090 -0.427929
C -0.805039  3.687345 -0.451076
C -1.345414  2.433052 -0.342234
O  5.230740 -0.807534  0.044198
H  3.865117 -2.923374  0.241077
H  3.606639  1.317359 -0.169120
H  2.488806  2.927870 -0.286003
H  1.012627  4.842301 -0.510114
H -2.418170  2.302329 -0.357639
C -2.551377 -0.151186 -0.106635
C -3.283722  0.003283  1.067215
H -2.752609  0.171386  1.997102
C -4.667751 -0.088546  1.024154
H -5.239876  0.025865  1.936093
C -5.317248 -0.324732 -0.181831
H -6.397375 -0.393814 -0.210421
C -4.582062 -0.467472 -1.351266
H -5.085278 -0.644849 -2.293157
C -3.196243 -0.378084 -1.317963
H -2.618368 -0.476256 -2.228916
C -0.912703 -2.400157  0.230887
C -0.950159 -3.183107 -1.068167
H -1.913139 -2.247393  0.617536
H -0.348966 -2.904978  1.009532
H -1.585848 -2.681435 -1.797865
H -1.367027 -4.170405 -0.868823
H  0.040994 -3.309945 -1.502393
F -0.772298 -0.452023  2.960721
H  1.449529 -3.105285  0.242477
C  5.921568  0.428902 -0.062501
H  6.980090  0.184914 -0.033647
H  5.674936  1.086890  0.773539
H  5.687331  0.927143 -1.005710
H -1.450074  4.549836 -0.552552

45
IIIf
C -3.664988 -1.986309 -0.165689
```

```
C -3.991843 -0.636049 -0.197636
C -3.057865  0.365398 -0.170113
C -1.705201  0.013787 -0.108563
C -1.331845 -1.341060 -0.083222
C -2.337050 -2.322578 -0.110182
C  0.073665 -1.682108 -0.038467
C  1.005280 -0.630410 -0.023820
C  0.553438  0.733871 -0.017074
N -0.724089  1.020435 -0.068970
C  0.539748 -3.006634 -0.014212
C  1.885084 -3.275204  0.017931
C  2.784383 -2.209596  0.021236
C  2.381992 -0.902954 -0.000920
H -4.435842 -2.742066 -0.185720
N -5.413864 -0.252336 -0.264097
H -2.073194 -3.368722 -0.085432
H -0.148827 -3.837118 -0.018220
H  2.246980 -4.292892  0.038771
N  4.227198 -2.502328  0.047236
H  3.110666 -0.106145  0.001188
C  1.566357  1.821231 -0.012974
C  2.149442  2.225059  1.184531
H  1.811784  1.770101  2.107225
C  3.132226  3.204606  1.162884
H  3.585729  3.526825  2.091519
C  3.536344  3.766742 -0.042212
H  4.305814  4.528366 -0.052885
C  2.959293  3.348568 -1.234135
H  3.278039  3.778196 -2.175087
C  1.972624  2.371631 -1.224593
H  1.527921  2.031495 -2.151763
C -1.173765  2.435820  0.002987
C -1.538312  2.987559 -1.362339
H -0.364797  3.003619  0.447437
H -2.002418  2.467666  0.705014
H -0.659858  3.028720 -2.005805
H -1.921562  3.999923 -1.235225
H -2.302506  2.390984 -1.859983
F -0.002697  0.515726  2.626624
H -3.395527  1.387176 -0.198938
O  4.561898 -3.665808  0.051765
O  4.996709 -1.568266  0.061690
O -6.232994 -1.143032 -0.260314
O -5.683430  0.926475 -0.320121

47
IIIg
C  4.310650 -0.158791 -0.069909
C  4.059078 -1.542034 -0.074055
C  2.777970 -2.017313 -0.068701
C  1.687761 -1.127027 -0.060014
C  1.929715  0.255870 -0.066764
C  3.254841  0.725820 -0.068688
C  0.803354  1.160189 -0.078056
C -0.491369  0.609334 -0.086684
C -0.667505 -0.813122 -0.047500
N  0.371347 -1.612949 -0.038771
C  0.943176  2.561437 -0.088035
C -0.155844  3.378544 -0.113604
C -1.427093  2.799526 -0.133625
C -1.620051  1.447824 -0.119880
O  5.605438  0.199857 -0.069885
H  4.897668 -2.226106 -0.079176
H  3.445398  1.786207 -0.067204
H  1.920144  3.019133 -0.075469
H -0.048482  4.453363 -0.121134
N -2.604937  3.678896 -0.172932
H -2.619753  1.040212 -0.133488
C -2.048240 -1.366389 -0.036187
C -2.732968 -1.476229  1.171097
H -2.220723 -1.208381  2.087383
C -4.039887 -1.942745  1.172089
H -4.574096 -2.035132  2.109122
C -4.661961 -2.287020 -0.022210
H -5.683009 -2.646890 -0.016495
C -3.977158 -2.164862 -1.224089
H -4.461941 -2.424796 -2.156380
C -2.667702 -1.701905 -1.235766
H -2.133080 -1.592551 -2.171460
C  0.169631 -3.081835  0.058411
C  0.284839 -3.756957 -1.295673
H -0.812206 -3.241959  0.487806
H  0.893473 -3.455114  0.776630
H -0.504198 -3.412414 -1.964015
H  0.175455 -4.832708 -1.158218
H  1.247192 -3.566339 -1.769784
F -0.037031 -1.029581  2.760288
H  2.635044 -3.085418 -0.074074
```

```
O -2.412420  4.874709 -0.158720
O -3.699678  3.163458 -0.219440
C  5.909243  1.587961 -0.051170
H  6.993245  1.659283 -0.049314
H  5.509029  2.060909  0.848189
H  5.510902  2.085009 -0.938367

41
IIIh
C  4.408389 -0.770767 -0.375016
C  3.785790 -2.021901 -0.348465
C  2.415939 -2.118161 -0.305818
C  1.628853 -0.955088 -0.287923
C  2.241682  0.309035 -0.320707
C  3.646392  0.370423 -0.362429
C  1.417297  1.495866 -0.309581
C  0.019252  1.335703 -0.280881
C -0.540195  0.019735 -0.235826
N  0.227420 -1.047605 -0.236250
C  1.942101  2.798989 -0.329363
C  1.104658  3.888859 -0.323528
C -0.287790  3.726617 -0.301864
C -0.825773  2.466240 -0.282192
H  4.379666 -2.926289 -0.361244
H  4.139430  1.331096 -0.383707
H  3.009586  2.959450 -0.347265
H  1.527563  4.885330 -0.336649
H -1.898503  2.335410 -0.265042
C -2.021397 -0.130405 -0.212625
C -2.706744 -0.071615  0.997621
H -2.143732  0.030953  1.918337
C -4.091400 -0.165653  1.001625
H -4.627031 -0.125182  1.941589
C -4.788064 -0.307836 -0.192640
H -5.868435 -0.378088 -0.184118
C -4.099522 -0.354097 -1.397651
H -4.639059 -0.457142 -2.330428
C -2.713590 -0.263392 -1.411810
H -2.172022 -0.287241 -2.349631
C -0.380941 -2.396726 -0.123143
C -0.492788 -3.083508 -1.471727
H -1.358473 -2.271946  0.326982
H  0.220562 -2.958109  0.585162
H -1.162760 -2.529378 -2.129087
H -0.905042 -4.081465 -1.321846
H  0.472891 -3.181468 -1.966514
Cl  0.290418 -0.485921  3.001259
H  1.969442 -3.098711 -0.290657
H -0.934690  4.593667 -0.299746
H  5.487694 -0.701081 -0.406165

40
IIIa+
C  2.596822  0.552827 -1.444269
C  2.953012 -0.769889 -1.722193
C  2.064697 -1.793596 -1.498306
C  0.788592 -1.515333 -0.981379
C  0.417785 -0.189670 -0.696119
C  1.350916  0.833286 -0.942993
C -0.898881  0.080294 -0.165696
C -1.779486 -1.002858  0.020044
C -1.342136 -2.328786 -0.286663
N -0.127181 -2.558748 -0.744615
C -1.343256  1.370376  0.167766
C -2.614559  1.566107  0.651457
C -3.494916  0.488056  0.822252
C -3.084850 -0.781792  0.511973
H  3.300072  1.354737 -1.626559
H  3.932348 -0.998074 -2.121996
H  1.085384  1.860424 -0.740134
H -0.691103  2.222584  0.050331
H -2.940432  2.567855  0.901623
H -4.494266  0.658988  1.199776
H -3.759330 -1.616418  0.641387
C -2.280980 -3.463510 -0.073741
C -2.405674 -4.019592  1.195893
H -1.798920 -3.645217  2.011734
C -3.307240 -5.053053  1.405643
H -3.401477 -5.488738  2.392090
C -4.088462 -5.521174  0.356142
H -4.793583 -6.325478  0.523573
C -3.971328 -4.953081 -0.905307
H -4.585237 -5.309222 -1.722771
C -3.068627 -3.920864 -1.124652
H -2.981127 -3.468323 -2.104766
C  0.305627 -3.949869 -1.044053
C  0.200742 -4.262510 -2.524683
H -0.324876 -4.616003 -0.467194
```

```
H  1.316879 -4.060877 -0.663768
H -0.836453 -4.201492 -2.853842
H  0.556680 -5.279245 -2.691687
H  0.797131 -3.584376 -3.133702
H  2.370173 -2.799797 -1.734765

42
IIIb+
C  4.346039 -0.712127 -0.197094
C  3.744887 -1.970863 -0.119428
C  2.377403 -2.091241 -0.054283
C  1.572494 -0.942338 -0.065257
C  2.164636  0.331082 -0.143869
C  3.566369  0.417032 -0.208376
C  1.321044  1.502418 -0.156295
C -0.072945  1.317870 -0.089675
C -0.618590 -0.005101 -0.016103
N  0.168957 -1.057992 -0.000018
C  1.828885  2.812559 -0.233600
C  0.985390  3.892059 -0.243246
C -0.393376  3.675331 -0.173279
C -0.939481  2.426507 -0.098562
H  5.423473 -0.626407 -0.248670
H  4.354412 -2.864941 -0.111591
H  4.043091  1.384291 -0.266484
H  2.892300  2.988319 -0.285739
H  1.374390  4.898322 -0.304048
N -1.293305  4.839819 -0.180790
H -2.010855  2.299074 -0.048712
C -2.094899 -0.174584  0.035733
C -2.744834 -0.154831  1.266104
H -2.170692 -0.048325  2.178694
C -4.125828 -0.277534  1.310310
H -4.633949 -0.268195  2.266075
C -4.852381 -0.409264  0.133312
H -5.930368 -0.503311  0.171671
C -4.199295 -0.414480 -1.092157
H -4.764918 -0.507373 -2.010460
C -2.817090 -0.294559 -1.146496
H -2.302511 -0.286519 -2.099441
C -0.417180 -2.426233  0.051009
C -0.447093 -3.062954 -1.325401
H -1.419132 -2.334748  0.452582
H  0.164295 -2.998622  0.767468
H -1.101836 -2.498648 -1.989863
H -0.839583 -4.075316 -1.230081
H  0.543420 -3.117068 -1.775206
H  1.950021 -3.078877 -0.001043
O -0.788611  5.936573 -0.271986
O -2.483950  4.639126 -0.094936

42
IIIc+
C  4.334095 -0.673858 -0.325365
C  3.709574 -1.913944 -0.242064
C  2.352498 -2.065155 -0.145271
C  1.553481 -0.915629 -0.122696
C  2.143725  0.357044 -0.207950
C  3.543501  0.445105 -0.309839
C  1.299214  1.531027 -0.186907
C -0.095136  1.344489 -0.107840
C -0.631680  0.019662 -0.030677
N  0.154966 -1.036964 -0.021166
C  1.801317  2.838996 -0.247512
C  0.942390  3.913715 -0.231140
C -0.443457  3.727013 -0.155877
C -0.960098  2.457881 -0.094865
H  5.408731 -0.601718 -0.404759
N  4.541108 -3.130084 -0.258358
H  4.017526  1.412119 -0.379253
H  2.864375  3.019884 -0.306027
H  1.345866  4.917226 -0.278337
H -2.029116  2.308925 -0.035774
C -2.107135 -0.159216  0.029469
C -2.758192 -0.157019  1.259426
H -2.186066 -0.057322  2.174047
C -4.138568 -0.287223  1.301140
H -4.646992 -0.289637  2.256804
C -4.864225 -0.409186  0.122534
H -5.941687 -0.508623  0.159291
C -4.210343 -0.397769 -1.102301
H -4.774514 -0.485255 -2.022047
C -2.828892 -0.269598 -1.153945
H -2.314328 -0.248632 -2.106811
C -0.424600 -2.406202  0.056834
C -0.510494 -3.049724 -1.314216
H -1.408493 -2.315702  0.501046
H  0.186866 -2.975050  0.751459
```

H -1.206683 -2.500395 -1.947967
H -0.878158 -4.068859 -1.196432
H 0.457515 -3.086601 -1.812700
H 1.945836 -3.060778 -0.092632
O 5.742472 -2.984136 -0.259934
O 3.980984 -4.203582 -0.268443
H -1.105231 4.582727 -0.144830

42
IIId+
C 3.687216 1.017791 -0.074732
C 3.592265 -0.368274 0.005259
C 2.346052 -0.938289 0.031662
C 1.194752 -0.133706 -0.015498
C 1.307456 1.265820 -0.093289
C 2.590219 1.831692 -0.125557
C 0.113892 2.080338 -0.140150
C -1.132626 1.423081 -0.138430
C -1.181670 -0.002378 -0.060925
N -0.079658 -0.725444 0.012836
C 0.135652 3.481506 -0.188751
C -1.040925 4.191875 -0.244337
C -2.280090 3.537065 -0.257133
C -2.328908 2.168614 -0.204789
N 5.024083 1.636256 -0.106063
H 2.727496 2.899558 -0.193021
H 1.071780 4.020198 -0.180394
H -1.006620 5.273379 -0.279989
H -3.281753 1.658569 -0.212589
C -2.506361 -0.679150 -0.057076
C -3.192023 -0.839172 1.143347
H -2.746115 -0.499141 2.070343
C -4.442393 -1.439767 1.140655
H -4.975577 -1.570050 2.073663
C -5.008234 -1.868225 -0.054018
H -5.985120 -2.334786 -0.052697
C -4.325566 -1.693142 -1.250242
H -4.768334 -2.017896 -2.183150
C -3.071770 -1.096081 -1.257126
H -2.537561 -0.948645 -2.187860
C -0.165667 -2.209852 0.105330
C 0.049526 -2.867261 -1.244205
H -1.147274 -2.449998 0.495668
H 0.558533 -2.528151 0.849582
H -0.729164 -2.563357 -1.943909
H -0.005208 -3.948176 -1.115124
H 1.019178 -2.621121 -1.675228
H 2.282020 -2.012272 0.082075
O 5.091134 2.839860 -0.216276
O 5.982708 0.902474 -0.017857
H -3.195680 4.111164 -0.306683
H 4.479936 -0.982530 0.040399

44
IIIe+
C 4.327201 -0.687844 -0.534582
C 3.727912 -1.956766 -0.437966
C 2.374023 -2.077303 -0.306092
C 1.557127 -0.930289 -0.262413
C 2.145007 0.339971 -0.365998
C 3.540782 0.442398 -0.504540
C 1.297109 1.510404 -0.328462
C -0.094367 1.325762 -0.210588
C -0.627144 0.003910 -0.098825
N 0.165812 -1.049192 -0.113555
C 1.794229 2.822376 -0.409297
C 0.937864 3.896470 -0.387312
C -0.448167 3.709774 -0.284166
C -0.959739 2.442089 -0.195487
O 5.665016 -0.684749 -0.655344
H 4.356619 -2.837008 -0.472831
H 3.995963 1.415205 -0.592810
H 2.855583 3.003947 -0.487064
H 1.339868 4.899630 -0.452601
H -2.027295 2.292703 -0.115583
C -2.097305 -0.174286 0.047684
C -2.677253 -0.058289 1.307624
H -2.053948 0.132204 2.173188
C -4.051393 -0.190762 1.445516
H -4.501645 -0.105184 2.426212
C -4.844742 -0.426694 0.329304
H -5.917155 -0.526966 0.439199
C -4.264114 -0.526261 -0.927976
H -4.880459 -0.700987 -1.800676
C -2.888910 -0.398360 -1.073691
H -2.433123 -0.466434 -2.053905
C -0.408636 -2.415184 0.014355
C -0.553667 -3.090262 -1.336238

```
H -1.373240 -2.316171  0.497901
H  0.231550 -2.971579  0.692741
H -1.246926 -2.534240 -1.967170
H -0.954145 -4.092536 -1.183205
H  0.397806 -3.175894 -1.859732
H  1.958032 -3.069789 -0.243341
C  6.327625  0.570267 -0.727557
H  7.387645  0.345386 -0.809548
H  6.145983  1.157412  0.175400
H  6.005012  1.132885 -1.606162
H -1.111025  4.564772 -0.272548

44
IIIf+
C  4.336081 -0.679531 -0.300244
C  3.709612 -1.917973 -0.222403
C  2.352177 -2.071020 -0.131280
C  1.553006 -0.922653 -0.119023
C  2.146786  0.349265 -0.198032
C  3.546360  0.440420 -0.286805
C  1.301339  1.522440 -0.186445
C -0.089828  1.333516 -0.094857
C -0.634177  0.008162 -0.023676
N  0.152144 -1.045230 -0.029532
C  1.807401  2.830021 -0.263430
C  0.960879  3.909557 -0.249212
C -0.413651  3.689584 -0.156169
C -0.958263  2.439129 -0.080887
H  5.411583 -0.607920 -0.369919
N  4.543042 -3.135302 -0.234476
H  4.024350  1.406503 -0.343736
H  2.868321  3.011318 -0.336329
H  1.347397  4.916888 -0.309438
N -1.317499  4.853265 -0.142625
H -2.028734  2.310361 -0.012888
C -2.108136 -0.162523  0.044746
C -2.743343 -0.176845  1.282477
H -2.159810 -0.097567  2.191619
C -4.123929 -0.299846  1.336906
H -4.622293 -0.316910  2.297612
C -4.862843 -0.398274  0.164181
H -5.940640 -0.491729  0.211522
C -4.223009 -0.370859 -1.067905
H -4.797865 -0.438755 -1.982551
C -2.841597 -0.250105 -1.133434
H -2.335904 -0.217116 -2.090616
C -0.431899 -2.415979  0.016320
C -0.487758 -3.034737 -1.367344
H -1.425509 -2.326802  0.438643
H  0.162646 -2.995667  0.716665
H -1.149593 -2.458622 -2.014248
H -0.883987 -4.045944 -1.277049
H  0.495118 -3.091222 -1.834618
H  1.947985 -3.067398 -0.075209
O -0.814076  5.951974 -0.202536
O -2.507461  4.645726 -0.073082
O  5.742283 -2.987450 -0.289456
O  3.983981 -4.207661 -0.188550

46
IIIg+
C  4.334864 -0.693857 -0.507934
C  3.733518 -1.961691 -0.411777
C  2.379247 -2.083215 -0.281501
C  1.563563 -0.936937 -0.239214
C  2.154815  0.334329 -0.338102
C  3.549904  0.438054 -0.472282
C  1.306118  1.503126 -0.304918
C -0.083946  1.313185 -0.184588
C -0.623140 -0.008889 -0.075959
N  0.170702 -1.057389 -0.098029
C  1.803997  2.817347 -0.391839
C  0.956882  3.893636 -0.370266
C -0.418247  3.669923 -0.263367
C -0.954805  2.418865 -0.169381
O  5.670378 -0.689998 -0.633486
H  4.362120 -2.842044 -0.446084
H  4.008870  1.409740 -0.550293
H  2.863608  3.002008 -0.477854
H  1.338961  4.902028 -0.438453
N -1.325518  4.828326 -0.253149
H -2.023774  2.288572 -0.088374
C -2.093312 -0.180496  0.070224
C -2.665182 -0.126192  1.338212
H -2.034397  0.006536  2.209005
C -4.040290 -0.247928  1.474631
H -4.485875 -0.211203  2.460478
C -4.840516 -0.410828  0.350414
```

```
H -5.913791 -0.501000  0.459642
C -4.266545 -0.449982 -0.913321
H -4.889739 -0.566988 -1.790649
C -2.890663 -0.333018 -1.058804
H -2.438790 -0.352393 -2.042992
C -0.410659 -2.425136  0.000695
C -0.554260 -3.065541 -1.366577
H -1.375533 -2.329067  0.484032
H  0.225257 -2.999503  0.667126
H -1.235820 -2.486797 -1.990128
H -0.968702 -4.065462 -1.237059
H  0.401454 -3.152496 -1.882489
H  1.964745 -3.076155 -0.220111
O -0.825800  5.930448 -0.293289
O -2.516688  4.617197 -0.206326
C  6.323168  0.567365 -0.756283
H  7.383706  0.348765 -0.843632
H  6.147132  1.183405  0.128345
H  5.981981  1.095090 -1.649556

40
IIIh+
C  4.348342 -0.703581 -0.208252
C  3.750600 -1.965385 -0.143066
C  2.383637 -2.087764 -0.082731
C  1.575316 -0.938979 -0.081182
C  2.163415  0.336290 -0.145024
C  3.565623  0.423167 -0.211264
C  1.318220  1.508249 -0.143584
C -0.077636  1.325440 -0.106860
C -0.614772  0.002384 -0.040147
N  0.173278 -1.054529 -0.015268
C  1.821907  2.818885 -0.182521
C  0.966190  3.894060 -0.197630
C -0.423738  3.709674 -0.175439
C -0.941937  2.442262 -0.129798
H  4.361497 -2.858590 -0.144892
H  4.039735  1.391893 -0.270126
H  2.886482  2.997123 -0.198711
H  1.372442  4.897167 -0.229460
H -2.012489  2.294264 -0.112822
C -2.091629 -0.173026  0.009746
C -2.752830 -0.064957  1.229533
H -2.187834  0.115055  2.136329
C -4.133761 -0.191185  1.273787
H -4.648050 -0.111445  2.222961
C -4.851933 -0.412652  0.104993
H -5.929794 -0.507364  0.142140
C -4.189279 -0.504724 -1.111610
H -4.746810 -0.667849 -2.025129
C -2.806904 -0.383353 -1.164186
H -2.286994 -0.444748 -2.112343
C -0.412411 -2.419263  0.066365
C -0.472518 -3.085118 -1.295261
H -1.405807 -2.320256  0.487546
H  0.181170 -2.981686  0.781147
H -1.126487 -2.525493 -1.963835
H -0.880059 -4.088888 -1.174052
H  0.509407 -3.165791 -1.759764
H  1.957583 -3.077055 -0.044605
H  5.425519 -0.614551 -0.259396
H -1.084422  4.566210 -0.192418

40
IIIa_vac+
C  2.615169  0.543658 -1.407546
C  2.970092 -0.779221 -1.684135
C  2.076454 -1.799455 -1.469219
C  0.795031 -1.518884 -0.966484
C  0.421540 -0.191193 -0.692108
C  1.364033  0.826090 -0.923418
C -0.903619  0.084612 -0.187347
C -1.783053 -0.999404  0.013588
C -1.345361 -2.325544 -0.276330
N -0.125407 -2.559412 -0.730484
C -1.361388  1.378010  0.109023
C -2.635678  1.576507  0.582824
C -3.508570  0.497105  0.782563
C -3.090785 -0.775643  0.501571
H  3.323896  1.342590 -1.577155
H  3.953174 -1.008588 -2.072441
H  1.102433  1.852771 -0.717252
H -0.718853  2.233475 -0.032118
H -2.970224  2.581850  0.803913
H -4.507771  0.671415  1.157400
H -3.756513 -1.613950  0.649992
C -2.280960 -3.461775 -0.061664
C -2.338649 -4.081187  1.183588
```

```
H -1.688911 -3.751321  1.985522
C -3.233386 -5.120567  1.390868
H -3.277662 -5.604560  2.357403
C -4.072515 -5.533286  0.364149
H -4.771022 -6.342480  0.530283
C -4.023620 -4.903183 -0.871262
H -4.685117 -5.214900 -1.668469
C -3.129459 -3.863435 -1.087751
H -3.103577 -3.355866 -2.044620
C  0.298193 -3.950461 -1.042860
C  0.187882 -4.249050 -2.528226
H -0.334982 -4.618958 -0.471441
H  1.309948 -4.070081 -0.665812
H -0.853240 -4.207504 -2.846415
H  0.560025 -5.255177 -2.717898
H  0.762657 -3.548974 -3.132505
H  2.382274 -2.806074 -1.701897

41
IVa
C  4.358473 -0.691034 -0.667709
C  3.787533 -1.951387 -0.542684
C  2.437677 -2.090314 -0.273531
C  1.612223 -0.964945 -0.123408
C  2.183080  0.314025 -0.274667
C  3.551217  0.422968 -0.532108
C  1.319605  1.492174 -0.151132
C  0.010813  1.329297  0.303720
C -0.507392 -0.037530  0.683498
N  0.248124 -1.105888  0.134789
C  1.754947  2.784045 -0.480306
C  0.925715  3.876445 -0.321729
C -0.367436  3.708503  0.167235
C -0.820118  2.438288  0.468242
H  4.396461 -2.840959 -0.647144
H  3.997850  1.404119 -0.619429
H  2.750313  2.937506 -0.873297
H  1.283777  4.864281 -0.583755
H -1.829677  2.302800  0.835380
C -1.988482 -0.180233  0.355911
C -2.961205 -0.254331  1.343329
H -2.676536 -0.237669  2.386295
C -4.303730 -0.353437  0.991784
H -5.056028 -0.414606  1.768334
C -4.678492 -0.372286 -0.343726
H -5.723967 -0.447818 -0.615373
C -3.704682 -0.290496 -1.333255
H -3.988550 -0.300571 -2.378195
C -2.366336 -0.193274 -0.983999
H -1.606238 -0.124852 -1.754279
C -0.284176 -2.459170  0.349425
C -0.564065 -3.198506 -0.949328
H -1.203638 -2.368919  0.922364
H  0.407464 -3.019786  0.980177
H -1.376917 -2.717833 -1.494985
H -0.856394 -4.227370 -0.732659
H  0.311433 -3.223638 -1.598891
F -0.394745 -0.105981  2.118767
H  2.034734 -3.086546 -0.180151
H -1.018766  4.562783  0.299542
H  5.416564 -0.578686 -0.864785

43
IVb
C -4.156972 -2.168491 -0.571622
C -4.606200 -0.857222 -0.457316
C -3.719912  0.177653 -0.226266
C -2.342667 -0.066658 -0.101115
C -1.881827 -1.391812 -0.243458
C -2.804280 -2.418823 -0.466014
C -0.446657 -1.649620 -0.162201
C  0.404546 -0.616964  0.241513
C -0.149323  0.733225  0.634673
N -1.449023  0.978579  0.121445
C  0.114385 -2.895684 -0.487714
C  1.467717 -3.119912 -0.385236
C  2.278679 -2.078568  0.046674
C  1.771975 -0.834985  0.354417
H -4.852709 -2.979897 -0.738511
H -5.662377 -0.632973 -0.541277
H -2.455842 -3.438934 -0.547695
H -0.513161 -3.703356 -0.834514
H  1.894859 -4.079925 -0.636383
N  3.720941 -2.301168  0.166605
H  2.437795 -0.048516  0.680583
C  0.817146  1.854308  0.276671
C  1.492290  2.582514  1.246459
H  1.317410  2.382581  2.294562
```

```
C  2.393317  3.572671  0.868756
H  2.914564  4.138939  1.630494
C  2.626087  3.832838 -0.473780
H  3.330087  4.602686 -0.764025
C  1.954661  3.098374 -1.445120
H  2.133110  3.293347 -2.495186
C  1.054945  2.112153 -1.070511
H  0.533396  1.535474 -1.826300
C -1.990750  2.329633  0.336443
C -2.325660  3.045784 -0.961787
H -1.248637  2.901340  0.888041
H -2.862566  2.262708  0.988696
H -1.416286  3.263155 -1.523164
H -2.828122  3.989523 -0.743542
H -2.981530  2.449288 -1.596754
H -4.113381  1.178341 -0.142309
O  4.154857 -3.390252 -0.146170
O  4.410330 -1.388662  0.571598
F -0.235666  0.700462  2.068008

43
IVc
C  3.596911  1.125811 -0.369920
C  3.478845 -0.241601 -0.203186
C  2.273932 -0.878159  0.009295
C  1.096798 -0.122476  0.055394
C  1.181640  1.272262 -0.149694
C  2.431176  1.864095 -0.339728
C -0.056039  2.056978 -0.158349
C -1.249091  1.443002  0.223878
C -1.266083  0.003606  0.682185
N -0.128522 -0.740286  0.264786
C -0.091905  3.401451 -0.554573
C -1.273004  4.115774 -0.532589
C -2.453130  3.503949 -0.118534
C -2.437450  2.172010  0.249221
H  4.559166  1.590777 -0.520790
N  4.695399 -1.065139 -0.246648
H  2.503011  2.935382 -0.460429
H  0.807877  3.895085 -0.893389
H -1.277761  5.153052 -0.843256
H -3.355728  1.689302  0.558527
C -2.558050 -0.695342  0.278482
C -3.520820 -1.062228  1.208507
H -3.355015 -0.879048  2.261348
C -4.700480 -1.666182  0.785236
H -5.446059 -1.953334  1.516218
C -4.923103 -1.899321 -0.564097
H -5.843092 -2.367373 -0.891349
C -3.960684 -1.526224 -1.496068
H -4.127611 -1.702764 -2.551363
C -2.784186 -0.925447 -1.075626
H -2.034429 -0.630535 -1.801262
C -0.142722 -2.178674  0.575357
C -0.062930 -3.055256 -0.663400
H -1.062052 -2.390433  1.115647
H  0.669106 -2.398172  1.270790
H -0.966055 -2.948032 -1.264967
H  0.031461 -4.101410 -0.368034
H  0.792293 -2.800809 -1.290628
H  2.274475 -1.947526  0.134405
O  5.759377 -0.497486 -0.375725
O  4.579253 -2.269122 -0.154285
H -3.380051  4.062227 -0.094280
F -1.252339  0.056775  2.115830

43
IVd
C -3.693196  1.024203  0.266537
C -3.629047 -0.347771  0.070876
C -2.403510 -0.932984 -0.142699
C -1.220369 -0.165966 -0.167929
C -1.308736  1.229476  0.057621
C -2.553637  1.805064  0.260620
C -0.079324  2.028486  0.062793
C  1.122601  1.420631 -0.292841
C  1.176324 -0.031393 -0.700696
N -0.003739 -0.769679 -0.380626
C -0.061990  3.384815  0.418326
C  1.110342  4.112025  0.383678
C  2.301929  3.503657 -0.004722
C  2.303763  2.162168 -0.332019
N -4.984228  1.656219  0.477219
H -2.653631  2.869343  0.409824
H -0.971131  3.879506  0.730816
H  1.100303  5.158003  0.663036
H  3.228596  1.678661 -0.621566
C  2.420129 -0.716383 -0.146084
```

```
C  3.445666 -1.163574 -0.967554
H  3.369316 -1.054562 -2.040980
C  4.574074 -1.755634 -0.408867
H  5.370088 -2.104081 -1.055119
C  4.682785 -1.895945  0.966851
H  5.563290 -2.354493  1.399465
C  3.657268 -1.442717  1.789767
H  3.734823 -1.547420  2.864788
C  2.531577 -0.854247  1.234576
H  1.730522 -0.501550  1.875146
C  0.043414 -2.212206 -0.673250
C -0.046799 -3.078411  0.572905
H  0.977568 -2.409377 -1.193432
H -0.750536 -2.451427 -1.381133
H  0.843997 -2.958179  1.189642
H -0.125363 -4.127710  0.284082
H -0.916387 -2.827090  1.180913
H -2.372957 -1.999213 -0.295284
O -5.020543  2.861925  0.633581
O -5.974066  0.949672  0.487429
H  3.222004  4.072603 -0.039846
F  1.298686 -0.033581 -2.122790
H -4.528356 -0.946422  0.079506

45
IVe
C  4.297704 -0.689307 -0.700748
C  3.720765 -1.949509 -0.551101
C  2.373076 -2.075715 -0.290509
C  1.543329 -0.948728 -0.172859
C  2.120925  0.316744 -0.343913
C  3.495096  0.431457 -0.595068
C  1.261079  1.500834 -0.252876
C -0.051416  1.351236  0.199358
C -0.569642 -0.005436  0.616012
N  0.168869 -1.082669  0.070999
C  1.700652  2.783459 -0.610296
C  0.872320  3.881192 -0.485121
C -0.424233  3.727438 -0.001677
C -0.880691  2.465741  0.329420
O  5.635588 -0.658399 -0.940126
H  4.345112 -2.830348 -0.634898
H  3.930321  1.413489 -0.695343
H  2.698411  2.927288 -1.000784
H  1.234527  4.861544 -0.768786
H -1.892573  2.342281  0.694142
C -2.058022 -0.147461  0.325475
C -3.010015 -0.180182  1.335244
H -2.703009 -0.129844  2.370728
C -4.360498 -0.280974  1.015929
H -5.096254 -0.309290  1.809996
C -4.764272 -0.343460 -0.309745
H -5.815847 -0.420933 -0.556118
C -3.811408 -0.302959 -1.321932
H -4.117771 -0.347051 -2.359627
C -2.465232 -0.203334 -1.004755
H -1.721647 -0.166646 -1.792861
C -0.359889 -2.431609  0.315100
C -0.627167 -3.204199 -0.966792
H -1.284604 -2.331839  0.877811
H  0.327277 -2.975822  0.965538
H -1.432697 -2.736216 -1.534288
H -0.925169 -4.226224 -0.726488
H  0.255352 -3.249616 -1.605798
H  1.973336 -3.071710 -0.179896
C  6.236650  0.610885 -1.119045
H  7.292100  0.425241 -1.303756
H  6.129213  1.228500 -0.223401
H  5.804110  1.134467 -1.976039
H -1.075059  4.585664  0.105029
F -0.426836 -0.041409  2.054724

45
IVf
C -3.574163 -1.984426 -0.294030
C -3.923291 -0.650488 -0.183471
C -3.008154  0.364588 -0.011523
C -1.643628  0.054942  0.054817
C -1.255525 -1.294355 -0.093140
C -2.228856 -2.283284 -0.248877
C  0.171207 -1.615705 -0.088359
C  1.083099 -0.621213  0.271670
C  0.608323  0.750563  0.693958
N -0.700577  1.056606  0.228746
C  0.655764 -2.881288 -0.452096
C  2.002744 -3.163424 -0.421465
C  2.876577 -2.161318 -0.025235
C  2.444200 -0.897944  0.312524
```

```
H -4.322662 -2.752477 -0.414432
N -5.349574 -0.289029 -0.239325
H -1.931784 -3.319017 -0.327620
H -0.022970 -3.656676 -0.775081
H  2.374980 -4.137849 -0.702407
N  4.314890 -2.449301  0.022136
H  3.159806 -0.141444  0.602292
C  1.606356  1.829965  0.298758
C  2.357238  2.511868  1.245968
H  2.223695  2.301476  2.298161
C  3.281482  3.469053  0.839894
H  3.861953  4.000589  1.583507
C  3.460433  3.741083 -0.508552
H  4.181027  4.485535 -0.823271
C  2.712798  3.051887 -1.457062
H  2.849328  3.256817 -2.511512
C  1.790406  2.097782 -1.054702
H  1.209851  1.556578 -1.793358
C -1.171761  2.434447  0.448158
C -1.532478  3.146706 -0.845011
H -0.379914  2.976453  0.958639
H -2.014892  2.416587  1.140889
H -0.643880  3.298397 -1.458487
H -1.965415  4.122248 -0.618519
H -2.257446  2.582786 -1.433185
H -3.372718  1.373925  0.071509
O  4.681235 -3.557837 -0.305090
O  5.062585 -1.566969  0.385539
O -6.157503 -1.189674 -0.310192
O -5.645788  0.886245 -0.211981
F  0.570221  0.713842  2.122658

47
IVg
C  4.221417 -0.015986 -0.286397
C  4.025853 -1.397418 -0.244483
C  2.761615 -1.925730 -0.112086
C  1.630908 -1.096010 -0.016198
C  1.826842  0.291029 -0.090898
C  3.122877  0.816277 -0.214224
C  0.657088  1.164914 -0.052976
C -0.588293  0.613363  0.265432
C -0.710048 -0.849229  0.628373
N  0.346645 -1.637698  0.113066
C  0.731461  2.537126 -0.343449
C -0.383299  3.342061 -0.287855
C -1.597418  2.764489  0.057029
C -1.718857  1.418236  0.326337
O  5.506491  0.408441 -0.394453
H  4.885866 -2.052371 -0.308806
H  3.259515  1.885948 -0.236754
H  1.671929  2.986556 -0.626712
H -0.321517  4.397221 -0.511548
N -2.791763  3.608430  0.130529
H -2.684817  1.007108  0.583346
C -2.072197 -1.408884  0.244421
C -3.031207 -1.724238  1.197259
H -2.809766 -1.604223  2.248754
C -4.277960 -2.195416  0.798475
H -5.020682 -2.440510  1.547406
C -4.570802 -2.347173 -0.548983
H -5.542371 -2.712577 -0.857275
C -3.612138 -2.024784 -1.503381
H -3.834481 -2.137020 -2.557241
C -2.368565 -1.555574 -1.107870
H -1.621472 -1.299958 -1.850570
C  0.223608 -3.093538  0.281362
C  0.271809 -3.844966 -1.038910
H -0.721109 -3.293136  0.780917
H  1.001165 -3.442898  0.962718
H -0.609035 -3.616156 -1.639863
H  0.291443 -4.920026 -0.853102
H  1.155562 -3.583832 -1.621998
H  2.666134 -2.999801 -0.083661
O -2.672553  4.785032 -0.140959
O -3.839308  3.092562  0.459846
C  5.730172  1.805503 -0.450371
H  6.806334  1.939543 -0.528629
H  5.369017  2.299055  0.455934
H  5.244911  2.248397 -1.324345
F -0.637844 -0.881029  2.067075

41
IVa_vac
C  4.371175 -0.691191 -0.601839
C  3.794187 -1.949911 -0.520083
C  2.437996 -2.088922 -0.288513
C  1.612143 -0.966276 -0.136113
```

```
C  2.188391  0.312339 -0.255743
C  3.561900  0.420700 -0.469284
C  1.321812  1.489011 -0.150488
C  0.009278  1.328143  0.290999
C -0.504384 -0.039156  0.681102
N  0.241659 -1.103943  0.095209
C  1.755176  2.776647 -0.491567
C  0.923119  3.868814 -0.354214
C -0.373167  3.703037  0.122518
C -0.825261  2.435376  0.432763
H  4.403491 -2.838454 -0.625559
H  4.012412  1.402174 -0.518849
H  2.751304  2.924886 -0.883241
H  1.281033  4.853790 -0.625215
H -1.838488  2.297799  0.787112
C -1.987939 -0.179394  0.361445
C -2.945797 -0.253501  1.362352
H -2.636629 -0.235727  2.398044
C -4.292644 -0.351924  1.030549
H -5.034206 -0.412924  1.816711
C -4.686256 -0.370873 -0.298355
H -5.735036 -0.445880 -0.554803
C -3.727565 -0.288862 -1.301432
H -4.027217 -0.296839 -2.341486
C -2.385353 -0.191420 -0.971984
H -1.632879 -0.119123 -1.748763
C -0.287780 -2.453552  0.313191
C -0.564493 -3.192028 -0.988881
H -1.209500 -2.363039  0.882405
H  0.400985 -3.012637  0.949762
H -1.389867 -2.718018 -1.519994
H -0.836884 -4.228631 -0.785040
H  0.305412 -3.187585 -1.645365
F -0.376685 -0.126894  2.092112
H  2.030464 -3.085173 -0.225575
H -1.027945  4.556542  0.237581
H  5.434469 -0.578211 -0.763198
```
